# Mercury Ion Sensing Using Mercaptosuccinic Acid-Derived Carbon Quantum Dots

**DOI:** 10.1021/acsomega.5c12026

**Published:** 2026-03-11

**Authors:** Haven I. Blair, Rayna E. Nemcek, Hallie G. McKinnie, Sarah Saleh, Madison L. Walker, Justin M. Miller, Deon T. Miles

**Affiliations:** † Department of Chemistry, 7303 The University of the South, 735 University Avenue, Sewanee, Tennessee 37383, United States; ‡ Department of Chemistry, 5718 Vanderbilt University, 7330 Stevenson Center, Station B 351822, Nashville, Tennessee 37235, United States; § Department of Chemistry, 171745 College of Basic and Applied Sciences, Middle Tennessee State University, 1301 East Main Street, Murfreesboro, Tennessee 37132, United States

## Abstract

Carbon quantum dots, prepared from mercaptosuccinic acid (MSA), were used to sense metal ions in aqueous solutions. Two fractions of nanoparticles were obtained after several purification steps, designated blue and green based on their color under UV illumination. We monitored the photoluminescence of the carbon nanoparticles upon the addition of metal ions. Stern–Volmer plots were made to determine whether photoluminescence quenching of the nanoparticles in solution occurred. Photoluminescence quenching was observed with adding Hg^2+^, Fe^3+^, Cr^3+^, Co^2+^, Ag^+^, Fe^2+^, Cu^2+^, and Ni^2+^. Most of these metal ions have partially filled d orbitals, contributing to the transfer of electrons from the photoexcited nanoparticles to the available empty orbitals of the metal ions. The detection limits for sensing the metal ions were calculated. The lowest detection limits observed were for Hg^2+^, with values of 4.1 and 1.4 ppm using the blue and green MSA-CQDs, respectively. Changes in the excitation wavelength and pH had a moderate impact on the spectral properties of the nanoparticles.

## Graphical abstract

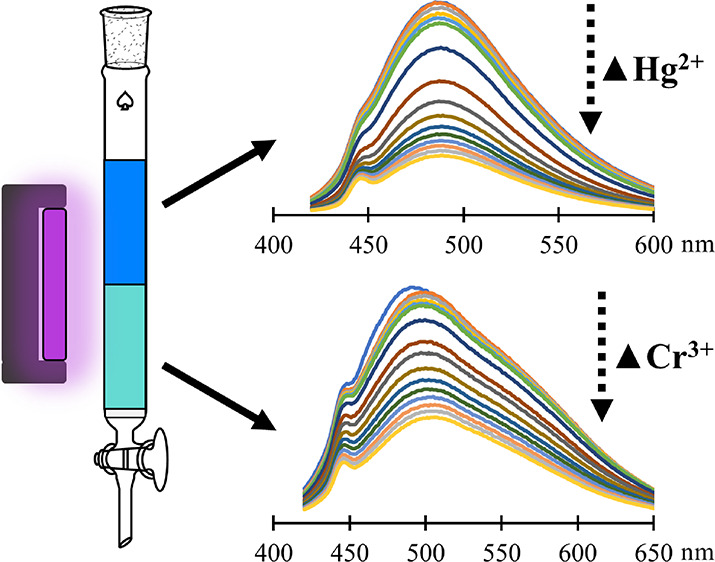

## 1 Introduction

Carbon quantum dots (CQDs) are zero-dimensional (<10 nm), carbon-based nanoparticles that have tunable photoluminescent properties. They have a core composed of both sp^2^ and sp^3^ carbon atoms.^1^ Since their accidental discovery in 2004, they have been considered a viable alternative to semiconductor-based quantum dots.^2^ These carbon nanoparticles have useful characteristics, including great stability, bright photoluminescence, low toxicity, water solubility, and biocompatibility. Because of these properties, carbon quantum dots have been studied thoroughly over the past two decades. Applications that use CQDs include sensors,^3–8^ bioimaging,^9–13^ photocatalysis,^14–16^ and optoelectronic devices.^17–19^ Over two years (2022 and 2023), over 7000 articles with the term “carbon dots” in either the title or abstract have been published.^20^ Despite this significant amount of research in the area of carbon nanoparticles, there are still many questions to be answered about their photoluminescent properties. Several factors impact these properties, including the chemical precursors, synthesis method, surface composition, and doping elements.

Carbon-based nanoparticles are prepared by two general synthetic categories: “top-down” methods or “bottom-up” processes. Top-down syntheses involve using larger carbon structures, such as graphene, graphite, or carbon nanotubes, and breaking them down into CQDs via laser ablation, arc discharge, hydrothermal/solvothermal synthesis, oxidative cutting, or electrochemical methods. Bottom-up syntheses involve smaller precursors, typically organic molecules and polymers, that are treated using hydrothermal/solvothermal pyrolysis, ultrasonication, and microwave-based pyrolysis.^21^ In this work, we use microwave-based pyrolysis for the CQD synthesis.^22^ Some advantages of the bottom-up methods include the ability to modify the surface easily and make various structures and functionalities. A disadvantage of the bottom-up techniques is that side products can form that require additional purification steps.^21^

The motivation to study heavy metal ion sensing stems from the tragic situations with contaminated water supplies, punctuated by the 2014 Flint, Michigan water crisis. While Flint was over a decade ago, more recent events, such as the 2023 East Palestine, Ohio train derailment, can harm a community’s water supply. Unplanned releases of hazardous waste, even when not directed to lakes, rivers, or streams, may reach groundwater sources as it percolates through the soil. East Palestine, for example, has numerous well water sources in their community.^23^ There is a significant risk of contaminated well water supplies in the eastern and southeastern United States.^24^ Being able to test these water supplies in an effective and cost-efficient manner is crucial to help keep communities safe. While it is ideal that there are no harmful metal ions in drinking water supplies, the reality is that there are accepted amounts allowed, which are called maximum containment levels (MCLs). For example, considering mercury as a contaminant, the United States Environmental Protection Agency (U.S. EPA) sets an MCL at 0.002 ppm.^25^ Therefore, any type of effective analytical technique to screen drinking water needs to aim for a detection limit at or below these MCLs.

One of the characteristic properties of the CQDs is their ability to photoluminesce. These nanoparticles exhibit bright photoluminescence (PL) upon UV irradiation (Figure 1). The PL of CQDs can occur from (1) the conjugation effect, associated with the carbon core, and (2) local surface states.^26^ The conjugation effect is related to size and refers to the sp^2^ (graphene) domain of the nanoparticle’s carbon core.^27^ At the nanoscale, quasi-continuous electronic levels near the Fermi level transform into discrete energy levels. A size increase of the core’s graphene domain results in more delocalization, which decreases the energy band gap between the highest occupied molecular orbital (HOMO) and the lowest unoccupied molecular orbital (LUMO). Local surface states refer to attached sites on the nanoparticle surface that can produce changes in electronic energy levels. There are two categories under consideration here: surface configurations and doping atoms. Surface configurations are associated with a surface structural type (e.g., defects and edge sites). Doping atoms are heteroatoms, such as nitrogen, boron, sulfur, selenium, tellurium, silicon, phosphorus, and halogens (F/Cl/Br/I),^28,29^ on the nanoparticle surface that can have an impact on the PL. Incorporating nitrogen as a dopant into CQDs has become a standard part of the synthesis process, with numerous examples prepared.^30,31^

**Figure 1:**
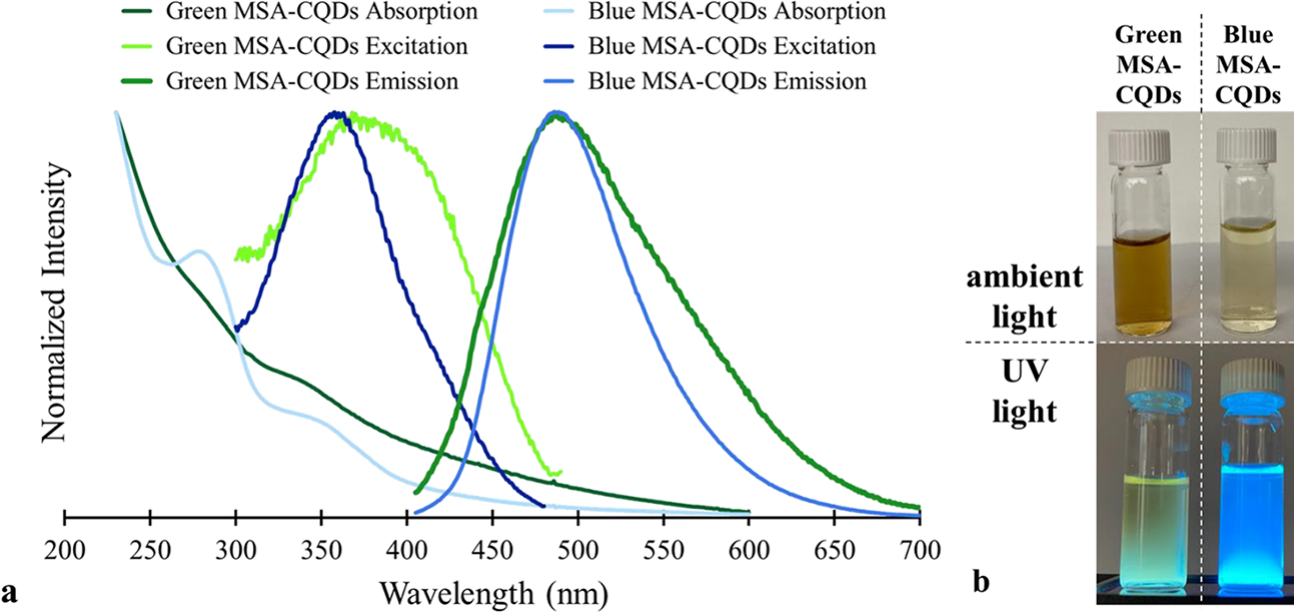
(a) Absorption, excitation, and emission spectra of the two MSA-CQD fractions (green and blue). For excitation spectra, λ_EM_ = 490 and 500 nm for green and blue fractions, respectively. For emission spectra, λ_EXC_ = 347 nm. (b) Digital images of the two MSA-CQD fractions under ambient light and UV light.

The mechanism of PL quenching can be divided into two categories: dynamic or static quenching. Dynamic quenching, or collisional quenching, depends upon the diffusion of a PL emitter (i.e., CQDs) or a quencher (i.e., metal ions) through a medium. When the emitter is excited, it collides with the quencher and returns the emitter to the ground state without the emission of a photon. Static quenching involves the interaction of the emitter and the quencher, forming a nonemissive species. The emission intensity is decreased because of the reduction in the number of available emitters.^32^ Some of the metal ions in this study have partially filled d orbitals. The electrons of the emitting CQDs can transition to the unfilled shells of the metal ions, resulting in PL quenching. Additionally, there may be the formation of complexes in the ground state between the CQDs and the metal ions, due to structural changes or changes in the number of surface states and traps.^33^

Metal ions in solution with the CQDs will likely interact through simple collisions. In some cases, the photoluminescence intensity of the CQDs will be reduced, or quenched, by these interactions with the metal ions. To quantify this, a Stern–Volmer plot is used to examine the interaction dynamics between a fluorescent emitter, CQDs for this experiment, and a quencher (e.g., metal ions). The plot shows the ratio of the photoluminescence intensity as a function of the quencher’s concentration (*Q*), as shown by eq 1

$$\frac{I_{0}}{I}=1+K_{\mathrm{SV}}[Q]$$ 1

where *I*_0_ and *I* are the photoluminescence intensity in the absence and presence of a quencher, respectively, and *K*_SV_ is the Stern–Volmer constant. A linear relationship supports dynamic quenching, where the quenching is mainly due to collisions between the fluorescent CQDs and quenching metal ions. A positive, nonlinear relationship supports the potential for dynamic and static quenching in the system. Static quenching can be verified by observing a change in the absorption spectrum. A negative deviation in the linearity of the plot indicates the presence of two emitters with different Stern–Volmer constants.^32^

While citric acid has been used extensively as a chemical precursor of carbon quantum dots, we used a starting material that has multiple carboxylic acid groups (like citric acid), but other functional groups to observe the impact on the resulting CQDs. In this work, we focused on mercaptosuccinic acid (MSA) as the primary carbon source because of its thiol group, along with its multiple carboxylic acid functional groups. The carboxylic acid groups should readily form amide bonds with urea, the added nitrogen source used, increasing the stability of the resulting CQDs. We investigated using carbon nanoparticles as sensors for metal ions in aqueous solutions. The thiol group of MSA should interact strongly with some of the metal ions used in this study. Additionally, doping sulfur into CQDs will make them more negatively charged, increasing the likelihood of interactions with metal ions.^34^ The metal ions selected for the study included four heavy metal ions (Hg^2+^, Cr^3+^, Pb^2+^, Cd^2+^) and various ions of biological and environmental significance (Na^+^, K^+^, Mg^2+^, Ca^2+^, Al^3+^, Fe^3+^, Mn^2+^, Co^2+^, Ag^+^, Fe^2+^, Cu^2+^, Zn^2+^, and Ni^2+^). The impact of varying the excitation wavelength and the pH on the spectral properties of the carbon quantum dots was investigated.

## 2 Experimental Methods

### 2.1 Materials

Mercaptosuccinic acid (Acros), urea (Fisher), quinine sulfate dihydrate (Thermo), sodium dihydrogen phosphate (Alfa Aesar), sodium hydrogen phosphate (Alfa Aesar), and silica gel (60 Å, 230–400 mesh, 40–63 μm) were used as received. All metal salts, lead­(II) nitrate (Acros), cadmium­(II) nitrate tetrahydrate (Aldrich), mercury­(II) nitrate monohydrate (Sigma-Aldrich), chromium­(III) nitrate nonahydrate (Aldrich), sodium nitrate (Baker), potassium nitrate (Fisher), silver nitrate (Sigma), magnesium nitrate hexahydrate (Acros), calcium nitrate tetrahydrate (Fisher), nickel­(II) nitrate hexahydrate (Lancaster), copper­(II) nitrate trihydrate (Lancaster), cobalt­(II) chloride hexahydrate (Fisher), manganese­(II) chloride tetrahydrate (Fisher), zinc nitrate hexahydrate (Alfa Aesar), iron­(II) sulfate heptahydrate (Fisher), iron­(III) nitrate nonahydrate (Sigma-Aldrich), and aluminum nitrate nonahydrate (Fisher), were used as received. Water was purified with a Millipore Elix 3 system.

### 2.2 Synthesis and Purification

For example, mercaptosuccinic acid (0.42 g, 2.8 mmol) and urea (0.50 g, 8.3 mmol) were combined in 10 mL purified water and stirred in a 100 mL round-bottom flask until the solution is transparent (5 min) (Scheme S1).^35^ After removing the magnetic stir bar, the flask was placed in a large beaker and covered with a watch glass. The mixture was heated in a conventional microwave oven (Hamilton Beach Model# P70B20AP-G5B) for 5 min at 700 W.^36^ After cooling, the resulting brown solid was dissolved in 10 mL purified water and sonicated (Fisher Scientific FS20 ultrasonic cleaner) for 5 min. The solution mixture was centrifuged (IEC Clinical centrifuge) for 15 min at 2300 rpm. The supernatant was collected and ran through a flash chromatography column (300 mm length, 40 mm O.D.) using silica gel and purified water as the mobile phase. The chromatography column was prepared from a slurry of 60 g silica gel and 200 mL purified water. To observe any separation of the supernatant into fractions, a UV lamp (254/365 nm, 4 W, Analytik Jena) was used alongside the column (fluorescence flash chromatography).^35,36^ During the chromatography step, the supernatant was separated into two fractions: the earlier eluting fraction that was green and the later eluting fraction that was blue. Sample fractions were collected and purified using dialysis. Dialysis tubing (Spectra/Por Biotech Cellulose Ester dialysis membranes) with molecular weight cutoff (MWCO) of 0.5–1 and 3.5–5 kDa was used. Samples were dialyzed for at least 48 h, with the purified water changed every 12–24 h. Dialysis was continued until the dialysate (the part of the mixture that passes through the membrane) no longer exhibited fluorescence under UV light.^37^ CQD samples were stored at 4 °C after dialysis until ready for use.

### 2.3 Characterization

A Jobin Yvon Fluoromax-P fluorimeter and a Horiba Duetta fluorescence and absorbance spectrometer were used to obtain fluorescence spectra of CQDs and make quantum yield measurements. Most emission measurements were made with an excitation wavelength of 350 nm, and an emission wavelength range of 360 to 900 nm. The excitation wavelength was adjusted (in 10 nm increments) between 300 and 420 nm for the excitation wavelength study. The corresponding emission wavelength range was λ_EXC_ + 10–900 nm. For example, when λ_EXC_ = 300 nm, the emission wavelength range was 310–900 nm. Excitation spectra were obtained in the 250–480 nm range for all samples, with the emission wavelength (λ_EM_) set at 490 nm. An upcycled OLIS 8453 UV–visible spectrophotometer was used to acquire absorption spectra of all samples. Samples for analysis were prepared in a 1 cm quartz cuvette (Fisher). Spectra were obtained in the 200–900 nm range for all samples. A Bruker Tensor 27 FTIR spectrometer equipped with an attenuated total reflectance (ATR) attachment was used to obtain IR spectra. Spectra were obtained in the 4000–520 cm^–1^ range, with 512 scans for each acquisition. A Zetasizer Nano ZS instrument and a Malvern Zetasizer Problue (Malvern Panalytical, Westborough, MA) were used for dynamic light scattering measurements. The instrument has a size detection range of 0.3 nm to 10 μm where 500 μL of sample was added to a disposable cell. High-resolution transmission electron microscopy (TEM) was performed using a FEI Tecnai Osiris operated at 200 keV. CQDs were deposited onto Lacey carbon-coated copper grids (Ted Pella) and allowed to air-dry overnight. Before sample deposition, the grids were plasma-cleaned for 2 min to enhance hydrophilicity and improve adhesion of the CQDs. This preparation ensured uniform distribution of the CQDs on the grids for high-resolution imaging of their morphology and size. TEM images were analyzed with ImageJ and Origin software.

### 2.4 Metal Ion Sensing

Metal ions were added to CQD solutions using the standard addition method. Metal ion solutions at 0.10 M were prepared from their corresponding metal salts. The two exceptions for the metal ions solutions prepared were Hg^2+^ (0.0010 M) and Fe^3+^ (0.010 M). CQD solutions were prepared by diluting 1.0 mL of the CQD sample to 100 mL using purified water. The diluted CQD solution was stirred at a low speed to encourage thorough mixing with the metal ion solutions. Metal ion solutions were added in the following increments (of total volume added): 0.10, 0.20, 0.30, 0.40, 0.50, 1.00, 2.00, 3.00, 4.00, 5.00, 6.00, 7.00, 8.00, 9.00, and 10.00 mL. After each addition of metal ions, the solution was allowed to stir for at least 1 min. Fluorescent spectra were collected after each addition of metal ion solution. To maintain the CQD concentration during the metal ion sensing experiment, the removed CQD + metal ion aliquots were returned to the diluted CQD solution after each spectral acquisition. Control experiments with additions of purified water at the same total volumes were conducted.

### 2.5 Fluorescence Quantum Yield

Determination of the fluorescent quantum yield was performed using a standard method.^38,39^ Quinine sulfate (in 0.1 M H_2_SO_4_, Φ = 0.54) was used as the standard for the analysis. The absorbance value of a CQD sample at 347 or 317 nm was adjusted from 0.10 to 0.02 absorbance units in regular increments. The integrated fluorescence area was measured for each of the CQD dilutions.

### 2.6 pH Study

A 0.10 M phosphate buffer at pH 7 was prepared by dissolving 8.2 g sodium dihydrogen phosphate and 5.1 g sodium hydrogen phosphate in 1 L purified water. HCl and NaOH solutions were added to ∼80 mL portions of the buffer to make solutions that varied by 1 pH unit in a pH range of 2–12. For each solution prepared for the pH study, 0.75 mL of the CQD solution was diluted to 10 mL using the specified pH-adjusted buffer. The corresponding fluorescence spectra were collected, and the wavelength maximum (λ_MAX_) and fluorescent intensity were recorded.

## 3 Results and Discussion

### 3.1 Absorption and Emission Spectroscopy

Two fractions of MSA-CQDs (designated green and blue based on their appearance under UV light) were isolated after fluorescent flash chromatography. The corresponding absorption spectra for the CQDs display strong absorbance in the UV region below 250 nm, with a tail extending into the visible region (Figure 1). The blue fraction has a clearly defined absorption feature around 280 nm. This absorption corresponds to the π–π* transition of the −C=C– of the sp^2^ carbons in the CQDs.^40^ The green fraction has no well-defined feature in the same wavelength range. The blue fraction also has an absorption feature near 350 nm. This absorption feature is attributed to the electronic transition from a nonbonding orbital (n; from −C=O or −NH_2_ groups present) to the π* orbital.^40,41^ The green fraction has a slight absorption feature in the same region but is rather ill-defined. The emission spectra show a subtle difference in emission wavelength maximum (λ_MAX_) for the two MSA-CQD fractions (∼485 nm for green and ∼490 nm for blue at an excitation wavelength = 347 nm). While the λ_MAX_ values are almost identical, there is a significant difference in the shape of the spectral features between the two fractions. The blue fraction has a narrower peak shape than the green fraction, with a full width at half-maximum (fwhm) of 88 and 126 nm, respectively. This difference in fwhm means less spectral clarity for the green MSA-CQDs. This supports some of the experimental findings from the quantum yield determination and the analysis of the excitation wavelength variation. The excitation spectra display the excitation wavelength range where photoluminescence is expected in the CQDs. For the blue MSA-CQDs, there is a narrower range of excitation spectra (300–450 nm) compared to that of the green MSA-CQDs (300–480 nm). This is reflected in the emission spectra in Figures 2 and S1, where significant photoluminescence is observed in these wavelength ranges. Also, the excitation spectra show a major difference in appearance compared to the absorption spectra. This is an initial indication that both Kasha’s Rule and Kasha-Vavilov’s Rule are not obeyed with these CQDs.^42,43^

**Figure 2:**
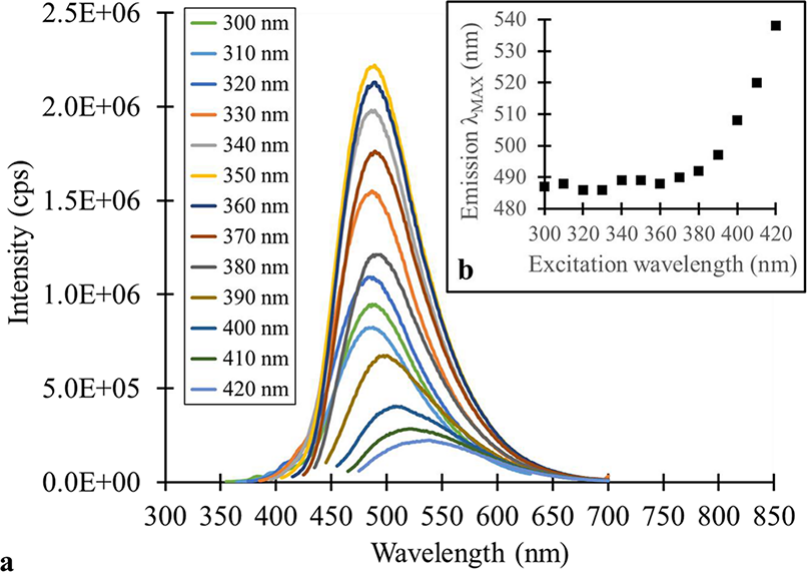
(a) Steady-state fluorescence spectra of the blue MSA-CQD fraction at various λ_EXC_ (300–420 nm), and (b) inset of emission λ_MAX_ vs λ_EXC_ for the blue MSA-CQD fraction.

### 3.2 Variation of Excitation Wavelength

Kasha’s rule is defined by IUPAC as follows: “Polyatomic molecular entities luminesce with appreciable yield only from the lowest excited state of a given multiplicity.”^42^ This means photoluminescence will always be generated from the vibrational ground state of the lowest excited singlet state (S_1_). Therefore, in a fluorescence spectroscopy experiment, if the excitation wavelength is changed, then no change in the resulting wavelength of maximum emission is observed. This means that excitation-independent photoluminescence is observed when Kasha’s rule is obeyed. However, if the emission wavelength changes as the excitation wavelength is altered, then Kasha’s rule is violated. This is excitation-dependent photoluminescence, which is observed in the carbon nanoparticles in this work.

The MSA-CQDs exhibited excitation-dependent behavior, which violates Kasha’s rule (Figure 2). The emission intensity was strongest for the blue MSA-CQD fraction for excitation wavelengths (λ_EXC_) from 300 to 370 nm, reaching a maximum emission at λ_EXC_ = 350 nm. The emission maxima were observed in the 485–490 nm range. However, at λ_EXC_ of 390 nm and above, the emission maxima have a clear red shift, reaching a λ_MAX_ ∼ 540 nm at λ_EXC_ = 420 nm (Figure 2b). Additionally, a decrease in photoluminescence observed at longer λ_EXC_ values corresponds to the absorption spectrum for the blue MSA-CQD fraction displaying diminished absorption at wavelengths beyond 400 nm (Figure 2a).

For the green MSA-CQDs, excitation-dependent behavior was observed throughout the selected excitation wavelengths, which differed from the blue fraction (Figure S1). The green MSA-CQDs displayed the lowest emission intensity at λ_EXC_ = 300 nm. The emission intensity steadily increased to a maximum when λ_EXC_ = 370 nm. Then, the emission intensity decreased as the λ_EXC_ values approached 420 nm. However, the decrease in emission intensity was not as significant as seen with the blue fraction. Also, a red shift of the emission maxima was observed, starting with λ_EXC_ = 390 nm. This was observed in the blue fraction as well. A shift in the wavelength of maximum photoluminescence emission toward higher wavelengths, caused by a shift in the excitation wavelength toward the red edge of the absorption band, is termed red edge excitation shift (REES).^44^ REES arises from relatively slow rates (compared to fluorescence lifetime) of solvent relaxation (reorientation) around an excited-state fluorophore. REES depends on the environment-induced motional restriction imposed on the solvent molecules near the fluorophore. The potential reasons for observing REES in nanoparticles include structural heterogeneity, the distribution of emissive states, and the surface trap states present in the nanomaterials.^37,45–47^ The REES observed in the CQDs is more prominent in the green fraction than in the blue fraction. Additionally, this excitation-dependent behavior for the MSA-CQDs suggested that the size distribution of the nanomaterials is polydisperse.

### 3.3 IR Spectroscopy

Infrared spectroscopy provides information about the functional group composition of the CQD surface. We analyzed the blue and green MSA-CQD samples using Fourier Transform infrared spectroscopy (Figure S2). In the region of 3600–3000 cm^–1^, a broad feature is observed, which corresponds to a combination of the N–H stretching of amines present (from the urea) and the O–H stretching of carboxylic acids (from the MSA). There are two features of interest, one around 1710 cm^–1^ and the other around 1660 cm^–1^. The peak at 1710 cm^–1^ indicates the presence of C=O stretching from carboxylic acid groups. It is expected that some free carboxylic acid groups are present on the CQD surface, which is supported by this observation in the IR spectra. The peak at 1660 cm^–1^ corresponds to the C=O stretching associated with an amide group. The formation of amides in the CQDs is expected with the numerous carboxylic acids and amines available from the chemical reagents used in the synthesis. Thus, we can use this feature as a diagnostic tool to confirm the formation of amide bonds in the CQDs. There are two additional features to consider in the IR spectra. The peak around 1400 cm^–1^ correlates to the O–H bending of carboxylic acids. Finally, a peak was observed in the region of 1100–1000 cm^–1^, which is attributed to the C–N stretching of amines. From the IR analysis, it is clear that the CQDs have formed through amide bond formation, and that there are carboxylic acid groups on their surface. These results correspond to previously reported IR analysis of CQDs.^48^

### 3.4 Transmission Electron Microscopy (TEM)

Transmission electron microscopy (TEM) imaging revealed that the carbon nanoparticles were well-dispersed across the Lacey carbon grids, with minimal aggregation. The CQDs appeared as roughly spherical nanoparticles with uniform contrast, indicating a homogeneous size distribution. Measured particle diameters were typically in the range of 1.7–2.6 nm for the blue MSA-CQDs, with an average diameter of 2.16 ± 0.21 nm and an average distribution of 4.76 ± 0.87 nm^2^ in area, as determined from their corresponding images and histograms (Figure S3). Green MSA-CQDs were in the range of 2–5 nm with an average diameter of 3.88 ± 0.68 nm and an average distribution of 1.85 ± 0.15 nm^2^ in area (Figure S4).

### 3.5 Dynamic Light Scattering

Our data presented thus far reveal strong similarities between green and blue MSA-CQDs. However, examination of Figure 1a,b suggests that differences do exist that drive differences in photophysical behaviors that include absorption and emissions. Given the relationship between CQD size and PL due to the conjugation effect,^26^ we sought to determine whether the subtle PL differences noted for green versus blue MSA-CQDs are due to particle size differences. To evaluate this question, Dynamic Light Scattering (DLS) techniques were employed to determine the size distribution profile, or dispersity, of nanoparticles suspended in a liquid.^49,50^ Briefly, DLS is a method wherein nanoparticle motion in solution is observed based on the intensity of the scattered light produced after exposure to a 600 nm laser source. By recording scattering intensities as a function of time, it becomes possible to back-calculate parameters such as the mean particle diameter and diffusion coefficient that describe the motion of a nanoparticle in solution using the Stokes–Einstein equation. Figure S5 reveals the resulting size distribution curves for blue (Figure S5a) and green (Figure S5b) MSA-CQDs when measured independently (*n* = 5). Each plot represents the percent frequency versus particle diameter, with the *x*-axis plotted on a logarithmic scale. Figure S5c summarizes the resulting analysis after the maximum particle diameter was determined for each replicate. A scatter plot of replicate measurements of the particle diameter for blue versus green MSA-CQDs reveals mean particle diameters equal to 112 ± 34 and 53 ± 26 nm, respectively. Application of an unpaired *t* test comparing mean values reveals statistically significant mean particle diameters based on *P* < 0.05. The steady-state fluorescence spectrum of the larger blue MSA-CQDs is slightly red-shifted compared to that of the smaller green MSA-CQDs (Figure 1a). The difference in mean particle diameter between the two fractions may correspond to these observed spectral differences. A difference in carbon nanoparticle size, with a small change in spectral properties, has been observed previously.^51^

Another aspect of interest for the nanoparticles is to determine their dispersity. Knowing the dispersity of the nanoparticles provides information about their size distribution in a particular sample. DLS can be used to estimate the size dispersity of a nanoparticle sample. The size distribution, which is assumed to be Gaussian, will provide a mean size and standard deviation based on the distribution statistics. The relative polydispersity is calculated by

$$relative\;polydispersity\;=\frac{standard\;deviation}{mean}$$ 2

For a theoretical Gaussian distribution, the overall polydispersity would be the relative polydispersity of the distribution. The overall polydispersity is converted into an overall polydispersity index (PDI), the square of the relative polydispersity. For a perfectly uniform sample, the PDI is zero. A monodisperse (narrow) distribution has a PDI between 0.0 and 0.1. Polydisperse distributions are classified as either “moderate” or “broad”, with a PDI of 0.1–0.4 and >0.4, respectively.^52^ Figure S5d reveals mean PDI estimates of 0.56 ± 0.09 and 0.37 ± 0.02 for blue versus green MSA-CQDs, respectively. Therefore, blue MSA-CQDs would be classified as broadly polydisperse, whereas green MSA-CQDs would be classified as moderately polydisperse. Taken together, these data support the excitation-dependent behavior of the CQDs by demonstrating statistically unique size distributions for each MSA-CQD type.

The zeta potential of the MSA-CQDs was measured using the DLS instrument. Zeta potential measures the magnitude of the electrostatic repulsion or attraction between nanoparticles in a liquid suspension. Zeta potentials have been measured previously in CQDs to determine the stability of these nanomaterials.^53^ For the current study, IR spectra confirmed the presence of carboxylic acids, amides, and amines on the surface of MSA-CQDs, which each have the potential to influence the surface charge. The rationale for undertaking such measurements is based on the fact that the zeta potential enables the prediction of nanoparticle stability. If the nanoparticles in solution have a large negative or positive zeta potential, they are more likely to repel each other. In this case, there would be little to no tendency for the nanoparticles to aggregate. However, if the nanoparticles in solution have a small zeta potential, there is a greater possibility that they will aggregate, thereby becoming unstable in solution. Figure S6 presents representative zeta potential distributions, where representative raw phase plots are presented in the inset. For blue (Figure S6a) and green (Figure S6b) MSA-CQDs, zeta potential distributions are qualitatively observed to adopt negative maxima. A general guideline for the designation between stable and unstable suspensions is ±30 mV. That is, nanoparticles with zeta potentials more positive than +30 mV or more negative than −30 mV are considered stable. To quantify stability for these nanoparticles, the mean zeta potential was estimated as −30.2 ± 1.5 and −27.4 ± 1.6 mV, respectively, for blue versus green MSA-CQDs. Individual replicate estimates are presented in Figure S6c, where unpaired *t* testing reveals the two means to be statistically unique based on *P* < 0.05. Taken together, the data presented here suggest that blue and green MSA-CQDs may exhibit moderate stability but should not be expected to have an indefinite shelf life.^54,55^

### 3.6 Quantum Yield

An extension of Kasha’s rule is highlighted in what is known as the Kasha-Vavilov rule. IUPAC defines this rule as “the quantum yield of luminescence is independent of the wavelength of exciting radiation.”^43^ If a change in excitation wavelength does not impact the quantum yield, then Kasha-Vavilov’s rule is obeyed. To confirm if Kasha-Vavilov’s rule is obeyed, the excitation wavelength used during a quantum yield experiment must be changed to see if it impacts the resulting quantum yield of the nanoparticle system. Two excitation wavelengths were selected for the quantum yield study (347 and 317 nm). These wavelengths were chosen because they corresponded to the absorbance peaks in the absorption spectrum (Figure S7) of the quinine sulfate reference standard (Φ_QS_ = 0.54 in 0.1 M H_2_SO_4_). We determined the photoluminescence quantum yield (Φ_CQD_) of the MSA-CQD fractions using a well-established method.^38,39^ Using absorption spectroscopy, the absorbance value of the CQD samples was adjusted from 0.10 to 0.01 at the two excitation wavelengths. We analyzed these diluted solutions using steady-state fluorescence spectroscopy, with the integrated fluorescence peak area calculated for each CQD sample. We produced plots of the integrated fluorescence peak area as a function of absorbance, and the resulting slope from the line of best fit was calculated for the CQD samples (*m*_CQD_) and quinine sulfate standard (*m*_QS_) (Figures S8 and S9). Eq 3 shows how the quantum yield is calculated for the nanomaterials, with the refractive index (*n*) incorporated into the calculation (*n*_CQD_ = *n*_QS_ = 1.33).^56^

$$\Phi_{\mathrm{CQD}}=\Phi_{\mathrm{QS}}\times \frac{m_{\mathrm{CQD}}}{m_{\mathrm{QS}}}\times \frac{n_{\mathrm{CQD}}^{2}}{n_{\mathrm{QS}}^{2}}$$ 3

For the blue MSA-CQDs, the quantum yield was 0.239 and 0.122 at excitation wavelengths of 347 and 317 nm, respectively. The quantum yield of green MSA-CQDs was 0.032 and 0.022 at λ_EXC_ of 347 and 317 nm, respectively. These quantum yield measurements clearly show that changing the excitation wavelength did alter the Φ_CQD_ values. Therefore, Kasha-Vavilov’s rule is violated for the MSA-CQDs. Additionally, the blue fraction has a significantly larger quantum yield than the green fraction. The blue fraction exhibits more spectral clarity than the green fraction.

### 3.7 Metal Ion Sensing

There are a handful of studies that have been published recently on the detection of Hg^2+^ using carbon quantum dots (Table 1). We explored the interaction between the MSA-CQDs and Hg^2+^, along with several other metal ions. For each experimental run, we prepared a diluted solution of the MSA-CQD fraction (for example, 1 mL of MSA-CQDs diluted to 100 mL with purified water). This diluted CQD solution was checked using absorption spectroscopy to ensure an absorbance of less than 0.10 at the excitation wavelength (350 nm). This was done to reduce errors from potential inner filter effects. Using the standard addition method, a 0.10 M metal ion solution was added to the CQD solution in precise increments (two exceptions: 0.0010 M Hg^2+^ and 0.010 M Fe^3+^ were used). After each addition, the resulting steady-state fluorescence spectrum was obtained (Figures 3a and 4a). To confirm that the decrease in photoluminescence intensity is due to interactions between the CQDs and the metal ions, and not from dilution effects, we designed a control experiment to examine how the photoluminescence changes from simple dilution. In the control experiment, we observed a slight decrease in photoluminescence intensity with the added volume of water from simple dilution (Figures S10 and S11).

**Table 1:** Summary of Recent Literature on CQD-Based Sensors for Hg^2+^ Detection

article title and authors	citation
investigation of fluorescence sensing capabilities in boron–nitrogen codoped carbon quantum dots toward Fe(III) and Hg(II) ions (R. Yadav et al.)	^57^
fluorescent carbon quantum dots for toxic mercury(II) ions detection in environmental waters (B. Altayli et al.)	^58^
highly luminescent nitrogen doped carbon quantum dots for mercury ion sensing with antibacterial activity (A. Dutta et al.)	^59^
o-phenylenediamine derived fluorescent carbon quantum dots for detection of Hg(II) in environmental water (A. Hu et al.)	^60^
diethylenetriamine-β-CD-modified carbon quantum dots for selective fluorescence sensing of Hg^2+^ and Fe^3+^ and cellular imaging (J. Yang et al.)	^61^
dual fluorometric detection of Fe^3+^ and Hg^2+^ ions in an aqueous medium using carbon quantum dots as a “turn-off” fluorescence sensor (S. Singh and S. K. Kansal)	^62^
detection of Fe^3+^ and Hg^2+^ ions by using high fluorescent carbon dots doped with S and N as fluorescence probes (H. Liu et al.)	^63^

**Figure 3:**
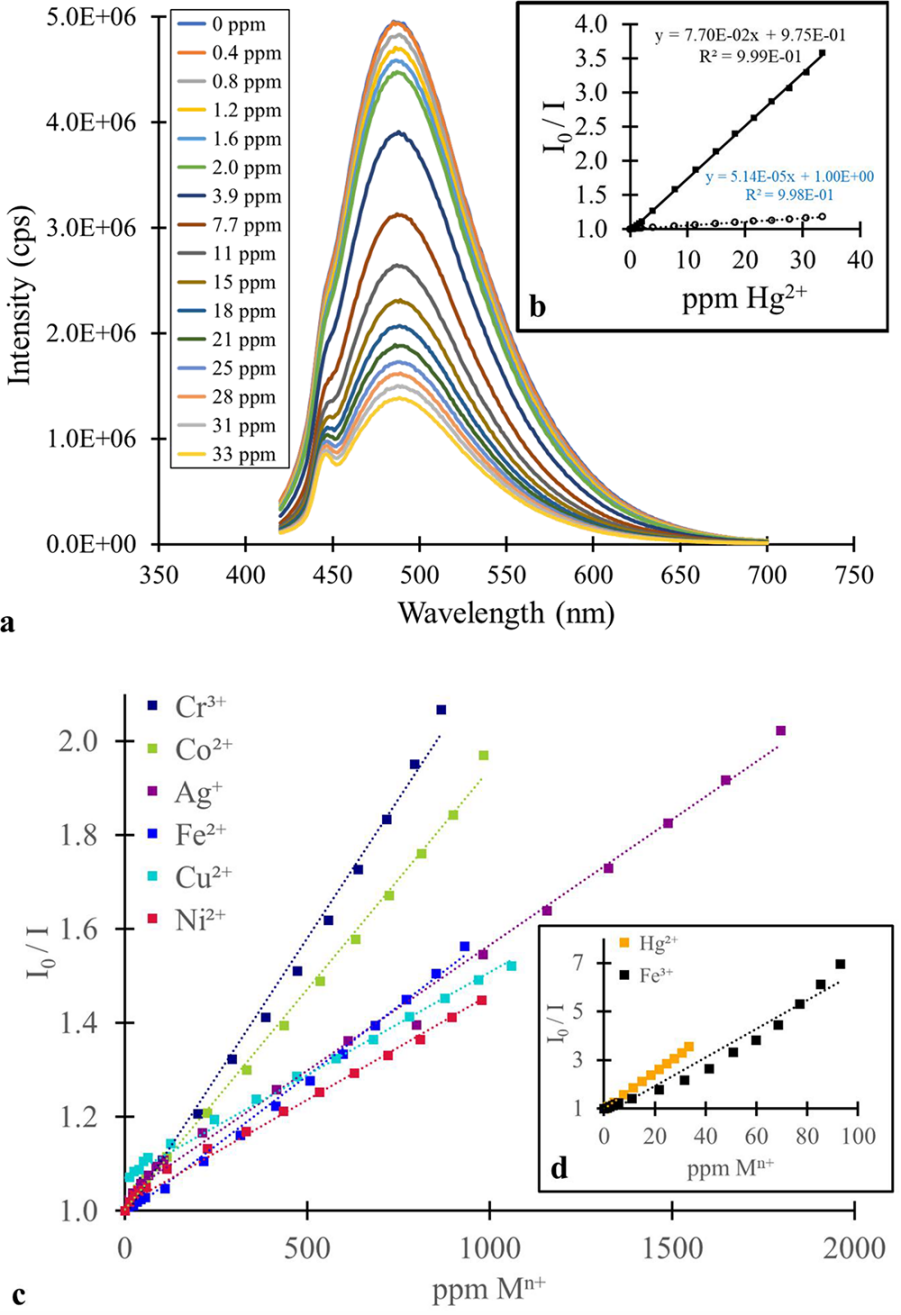
Fluorescence quenching of blue MSA-CQDs with metal ions: (a) steady-state fluorescence spectra (λ_EXC_ = 350 nm) with the addition of Hg^2+^, (b) inset is the corresponding Stern–Volmer plot (■ = CQD interaction with Hg^2+^, ○ = control experiment with same volumes of water added as metal ion solution, (c) composite of several Stern–Volmer plots with the addition of various metal ions, and (d) inset is the Stern–Volmer plot with the addition of Hg^2+^ and Fe^3+^ (separated for clarity).

**Figure 4:**
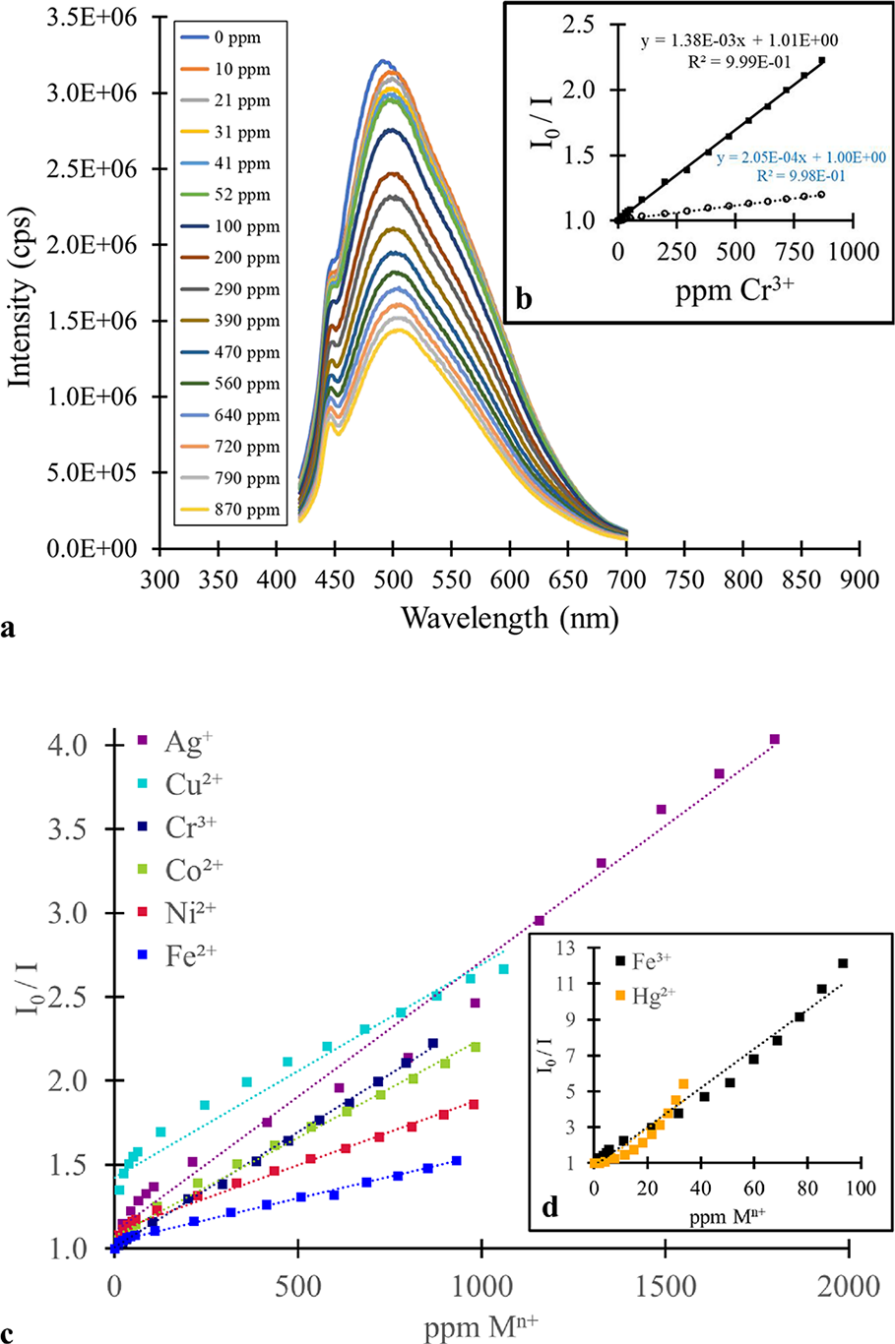
Fluorescence quenching of green MSA-CQDs with metal ions: (a) steady-state fluorescence spectra (λ_EXC_ = 350 nm) with the addition of Cr^3+^, (b) inset is the corresponding Stern–Volmer plot (■ = CQD interaction with Cr^3+^, ○ = control experiment with same volumes of water added as metal ion solution, (c) composite of several Stern–Volmer plots with the addition of various metal ions, and (d) inset is the Stern–Volmer plot with the addition of Hg^2+^ and Fe^3+^ (separated for clarity).

We prepared Stern–Volmer plots for the metal ion quenching of the CQDs (Figures 3b and 4b). We compared these plots with a Stern–Volmer “plot” from the control experiment results. Specifically, we compared the slopes of the lines of best fit. If the slope ratio (i.e., metal ion: water control) was greater than 2, we concluded that metal ion quenching occurred in this instance. We observed photoluminescence quenching for the blue and green MSA-CQD fractions for Hg^2+^, Fe^3+^, Cr^3+^, Co^2+^, Ag^+^, Fe^2+^, Cu^2+^, and Ni^2+^ (Figures 3 and 4). Most of these metal ions have partially filled d orbitals, with Hg^2+^ being the lone exception. Metal ions with partially filled d orbitals can interact with the electrophilic carboxylate functional groups to accept electrons from the photoexcited CQDs. Each mercaptosuccinic acid molecule has two carboxylic acid groups, providing numerous locations for the metal ions to interact with the CQDs. The metal ion sensing experiments were conducted in pH-neutral solutions (i.e., pH = 7). The p*K*_a_ values for the carboxylic acid groups on MSA are 3.30 and 4.60. Therefore, the carboxylic acid groups are deprotonated at neutral pH, producing an electrophilic environment for the metal ions.

CQDs, like semiconductors, can exhibit electron–hole properties. When an electron is excited in a CQD, it can move from the highest occupied molecular orbital (HOMO), or valence band, to the lowest unoccupied molecular orbital (LUMO), or conduction band. This movement creates a hole in the valence band. The excited electron can then drop back into the valence band, releasing energy as light. The color of the light depends on the energy difference between the two bands. For the metal ions with partially filled d orbitals, unfilled shells are available for the photoexcited electrons of the CQDs. If these electrons fill these shells, instead of returning to the valence band of the CQDs, then the photoluminescence will be quenched. Also, the CQDs and metal ions may form ground-state complexes, leading to a decrease in photoluminescence.

Some of the metal ions with partially filled d orbitals (Fe^3+^, Cr^3+^, Co^2+^, Ag^+^, Fe^2+^, Cu^2+^, and Ni^2+^) quenched the photoluminescence of the CQDs. However, Mn^2+^, which is isoelectronic with Fe^3+^, did not quench the CQD photoluminescence. A comparison of the ionic radii for these metal ions can provide some insights into this difference. The ionic radius for Mn^2+^ is larger than that of Fe^3+^ (Table 2).^64^ The larger size of the Mn^2+^ may limit interaction with the carboxylate groups on the CQD surface, minimizing quenching. Comparing the other metal ions with partially filled d orbitals (Cr^3+^, Co^2+^, Ag^+^, Fe^2+^, Cu^2+^, and Ni^2+^), only Ag^+^ has an ionic radius larger than Mn^2+^. However, the quenching observed using Ag^+^ is likely due to its interactions with the thiol groups present in the CQDs.^65^

**Table 2:** Ionic Radii (pm) of Metal Ions with Partially Filled d Orbitals (Coordination Number = 6)^64^

	**Mn** ^**2+**^	**Fe** ^**3+**^	**Cr** ^**3+**^	**Fe** ^**2+**^	**Co** ^**2+**^	**Ni** ^**2+**^	**Cu** ^**2+**^	**Ag** ^**+**^
low spin	67	55		61	65			
high spin	83	64.5		78	74.5			
			61.5			69	73	115

Another factor when considering how the metal ions interact with the carbon nanoparticles is related to Pearson’s hard soft acid base (HSAB) theory, which is used to classify hard and soft acids and bases.^66^ Hard acids and bases have smaller atomic or ionic radii, high oxidation states, and lower polarizability. Conversely, soft acids and bases have larger atomic or ionic radii, low oxidation states, and higher polarizability. The metal ions used can be classified into three categories: hard acids, “intermediate” acids, and soft acids (Table 3).

**Table 3:** Metal Ions Classified Using HSAB Theory

Classification	Metal ions
hard acids	Na^+^, K^+^, Mg^2+^, Ca^2+^, Cr^3+^, Al^3+^
“intermediate” acids	Mn^2+^, Fe^2+^, Fe^3+^, Co^2+^, Ni^2+^, Cu^2+^, Zn^2+^, Pb^2+^
soft acids	Ag^+^, Cd^2+^, Hg^2+^

The surface of the carbon quantum dots contains carboxylic acid, amine, and thiol functional groups. In HSAB theory, the carboxylic acid and amine groups are considered hard bases, while the thiol group is classified as soft. Most of the metal ions with full d orbitals did not quench the photoluminescence of the CQDs. The one exception was the Hg^2+^ ion, where we observed the most quenching, which has filled d-orbital and f-orbital subshells. According to HSAB theory, Hg^2+^ is a soft acid. With the thiol group of the MSA, a soft base, available on the CQD surface, it is likely that a complex is made between the CQDs and the mercury ions. The formation of this complex reduces the photoluminescence of the nanoparticles.

The photoluminescence of the MSA-CQDs was quenched with two of the three soft acids (Hg^2+^ and Ag^+^) examined. No photoluminescence quenching was observed for the soft acid Cd^2+^. Cd^2+^ does not have partially filled d orbitals, which is a reason for this absence of interaction with the CQDs. Both Cd^2+^ and Hg^2+^ lack partially filled d orbitals, and they are comparable in ionic radius with Hg^2+^ (109 pm vs 116 pm for Cd^2+^ and Hg^2+^, respectively). The observation of strong quenching with Hg^2+^ is likely because the thiol groups of the MSA-CQDs interacted strongly with the metal ion.^67^ It has been observed that there is a lack of interaction between thiols and several metal ions (Cd^2+^, Pb^2+^, Zn^2+^, Co^3+^, Fe^3+^, Cu^2+^, and Ni^2+^).^68^

It should be noted that a spectral artifact is observed around 445 nm in the fluorescence spectra (Figures 3a and 4a). This erroneous feature, which is more pronounced at lower fluorescence intensities, is related to an issue with our fluorescence spectrometer, which is nearing the end of its useful lifetime.

In the PL quenching for Fe^3+^ with the blue and green fractions of MSA-CQDs, the Stern–Volmer plot shows a positive deviation from linearity (Figures 3d and 4d). A positive, nonlinear relationship is characteristic of a system where dynamic and static quenching occurs. Static quenching involves the interaction of the emitter and the quencher, forming a nonemissive species. The emission intensity is decreased because of the reduction in the number of available emitters. Static quenching can be verified by observing a change in the absorption spectrum. The absorption spectra of the blue MSA-CQDs were examined with added Fe^3+^ (Figure S12). The blue MSA-CQDs, without any added Fe^3+^, have two features in the absorption spectrum (280 and 350 nm). With added Fe^3+^, the absorption spectrum changes for the blue MSA-CQDs, with a peak observed around 300 nm. The change in absorption spectra supports the observation of static quenching when Fe^3+^ is added to the MSA-CQDs.

In most instances, a total of 1.0 mmol of the metal ion solution was added to the diluted CQD solution. This was not the case for Hg^2+^ and Fe^3+^, where 0.010 and 0.10 mmol were added to the CQD solution, respectively. Figure 5 shows the impact on the CQD photoluminescence intensity in the presence and absence of the metal ion quencher in the form of a heat map, where more red color indicates more quenching and more blue represents less quenching. For nine of the metal ion solutions (Na^+^, K^+^, Ca^2+^, Mg^2+^, Zn^2+^, Al^3+^, Cd^2+^, Mn^2+^, and Pb^2+^), there is no effective change in the photoluminescence intensity of the CQDs upon addition of the metal ions, hence the nanoparticles are not selective for them. However, for the remaining metal ions (Fe^2+^, Ni^2+^, Co^2+^, Cu^2+^, Ag^+^, Cr^3+^, Fe^3+^, and Hg^2+^), quenching of the CQD photoluminescence was observed. The strongest quenching was observed with Fe^3+^ and Hg^2+^, with a smaller amount of these metal ions used to observe a significant decrease in photoluminescence. The MSA-CQDs were most selective for Hg^2+^ and Fe^3+^, which was the reason why smaller molar amounts of these metal ions were added to the carbon nanoparticle solution during the quenching study. A rough selectivity order for the MSA-CQDs, based on the heat map, is Hg^2+^ ≫ Fe^3+^ ≫ Ag^+^ > Cr^3+^ ≈ Cu^2+^ ≈ Co^2+^ > Ni^2+^ ≈ Fe^2+^. Differences were observed in the quenching between the blue and green MSA-CQDs. For most of the metal ions that quenched the CQDs, the green fraction had a larger photoluminescence intensity decrease than the blue fraction. In Figure 5, the photoluminescence intensity ratio (*I/I*_*0*_) was normalized against the control experiment, where water was added to the CQD solution instead of the metal ion solution. The corresponding *I*/*I*_0_ value obtained from the control experiment was used as the divisor. Also, the concentration of the metal ion solution, in millimolar concentrations, was factored in to calculate the normalized *I*/*I*_0_ used, as shown in eq 4 below. Effectively, smaller normalized *I*/*I*_0_ values indicate that more quenching has occurred.

$$\mathrm{normalized}\;I/I_{0}=\frac{I/I_{0}(\mathrm{metal}\;\mathrm{ion}\;\mathrm{addition})}{I/I_{0}(\mathrm{water}\;\mathrm{addition})}\times [M^{n+}](\mathrm{mM})$$ 4

**Figure 5:**
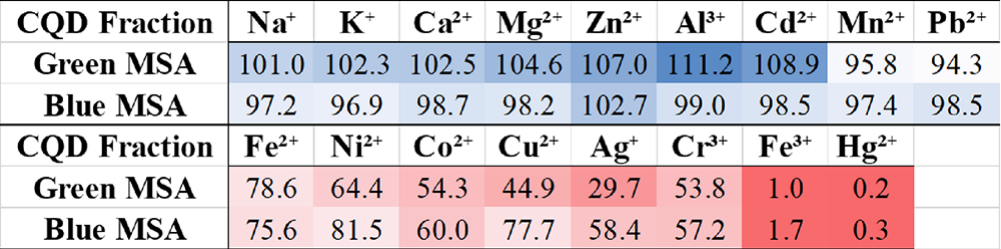
Heat map of blue and green MSA-CQDs in the absence and presence of various metal ions.

The limit of detection (LOD) is the lowest signal that can be observed with a certain confidence level (we use 90% confidence here). In particular, the signal associated with the detection limit can be discerned from the background noise associated with the measuring instrument. Knowing the LOD for metal ion sensing by the CQDs provides a quantitative measure of the effectiveness of the nanoparticles as sensors. From the fluorescence spectra of CQDs interacting with metal ions (Figures S13–S48), we can plot the fluorescence intensity at increasing metal ion concentrations. From these plots, the detection limit can be calculated using eq 5:

$$\mathrm{LOD}=\frac{3\times s_{b}}{m}$$ 5

where *s*_b_ is the standard deviation of the intercept and *m* is the slope from the line of best fit. The detection limits for metal ions are shown in Table 4. The lowest detection limits observed were for Hg^2+^, with values of 4.1 and 1.4 ppm using the blue and green MSA-CQDs, respectively. There are multiple analytical techniques used to detect mercury in water samples, including spectroscopy, electrochemistry, chromatography, and colorimetry.^69^ For example, using UV–visible spectrophotometry, a detection limit of 0.016 ppm was recently reported for the determination of mercury in water.^70^ Using differential pulse stripping voltammetry, a detection limit of 0.0058 ppm has been reported.^71^ Using a combination of liquid chromatography and inductively coupled plasma mass spectrometry, the detection limit was reported as low as 0.00008 ppm.^72^ Colorimetry has been used for mercury analysis, with the incorporation of various nanoparticles.^69^ A detection limit of 0.01 ppm was reported for the use of silver nanoparticles and UV–visible spectrophotometry.^73^

**Table 4:** Limits of Detection (ppm) for the Metal Ions

quenching metal ions								
CQD	Hg^2+^	Cr^3+^	Fe^3+^	Fe^2+^	Co^2+^	Cu^2+^	Ni^2+^	Ag^+^
blue MSA	4.11 ± 0.33	42.1 ± 1.3	10.98 ± 0.85	20.42 ± 0.29	50.7 ± 1.7	96.1 ± 5.7	89.1 ± 5.3	119.4 ± 5.2
green MSA	1.409 ± 0.039	62.3 ± 2.9	19.7 ± 2.7	62.4 ± 2.7	107.8 ± 7.7	274 ± 46	109.7 ± 8.0	233 ± 20

nonquenching metal ions									
CQD	Pb^2+^	Cd^2+^	Mn^2+^	Na^+^	K^+^	Ca^2+^	Mg^2+^	Zn^2+^	Al^3+^
blue MSA	140.3 ± 3.7	63.5 ± 1.4	15.61 ± 0.17	15.94 ± 0.43	32.0 ± 1.0	21.22 ± 0.44	10.97 ± 0.19	35.72 ± 0.76	13.06 ± 0.25
green MSA	203.3 ± 7.8	339 ± 40	26.96 ± 0.52	15.06 ± 0.39	9.755 ± 0.095	8.424 ± 0.069	22.52 ± 0.82	68.6 ± 2.8	73.9 ± 7.9

### 3.8 Variation of pH

The CQDs were investigated in different environments to see if their physical properties changed. The spectral properties of the MSA-CQDs under varied pH conditions were examined (Figure 6). The photoluminescence intensity changes with pH for both fractions, but with different outcomes. The blue MSA-CQD fraction displayed diminished photoluminescence under alkaline conditions (pH > 8). For the blue fraction at pH 12, the photoluminescence intensity was about 50% of the maximum intensity (measured at pH 3). Conversely, the green MSA-CQD fraction showed an increase in photoluminescence intensity under alkaline conditions. At pH 2, the photoluminescence intensity was about two-thirds of the maximum intensity (measured at pH 11) for the green fraction. An examination of the p*K*_a_ values for MSA, which are 3.30 and 4.60 for the carboxylic acid groups, and 10.38 for the thiol group, does not provide any insight into the spectral differences. At pH > 11, all of the functional groups are deprotonated, increasing the negative character of the CQDs. The blue and green fractions had negative zeta potentials from the dynamic light scattering results. This indicates that negative charges are dominant, in solution, at the nanoparticle surface.

**Figure 6:**
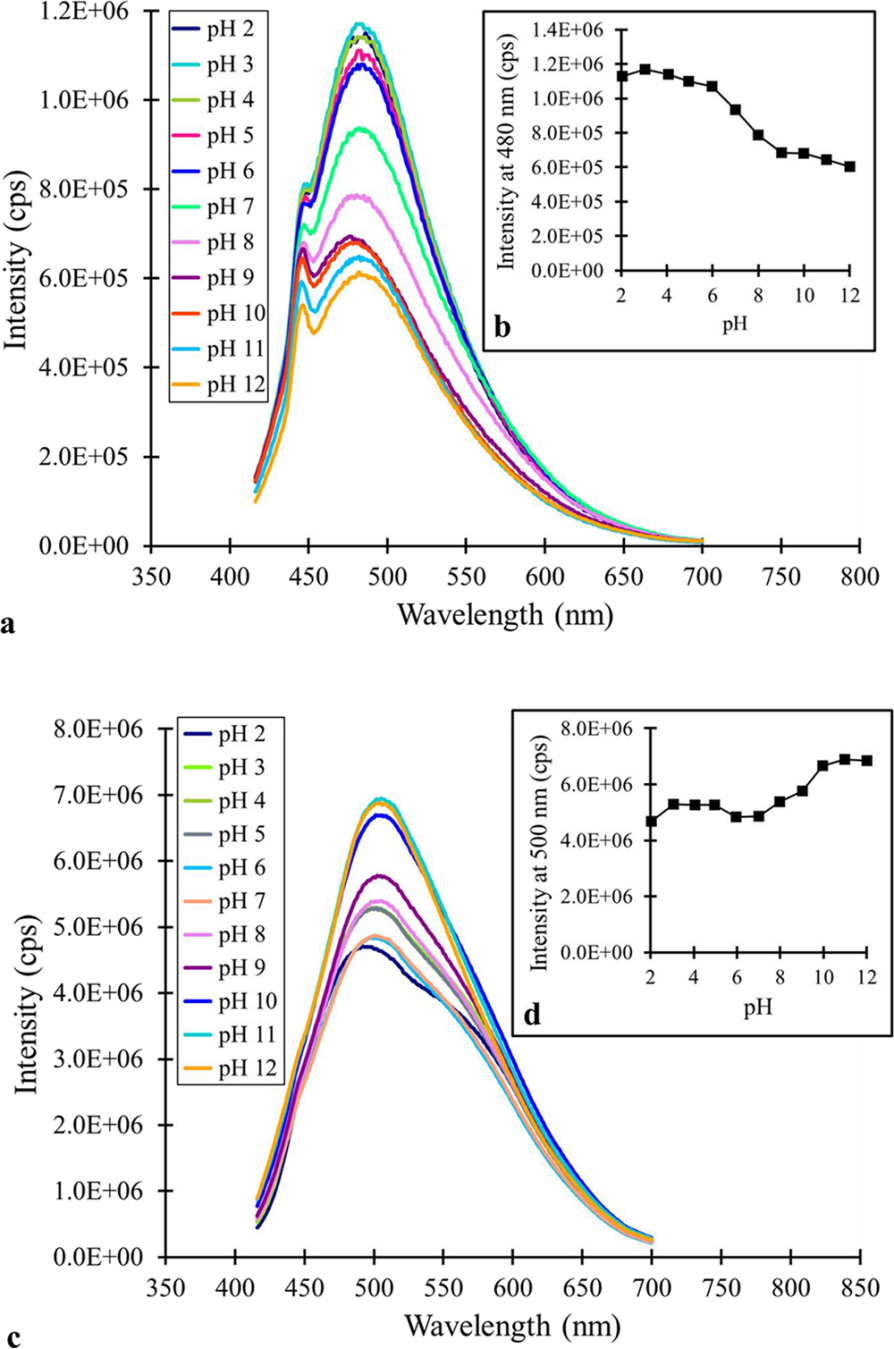
Impact of pH changes: (a) steady-state fluorescence spectra (λ_EXC_ = 350 nm) of the blue MSA-CQD fraction at various pH values, (b) inset of fluorescence intensity (at 480 nm) of the blue MSA-CQD fraction at various pH values, (c) steady-state fluorescence spectra (λ_EXC_ = 350 nm) of the green MSA-CQD fraction at various pH values, (d) inset of fluorescence intensity (at 500 nm) of the green MSA-CQD fraction at various pH values. Solutions were buffered in a 0.1 M NaH_2_PO_4_/Na_2_HPO_4_ buffer.

## 4 Conclusions

Carbon quantum dots were prepared from mercaptosuccinic acid as the primary carbon source. After several purification steps, two fractions of nanoparticles were collected, each with its unique spectral properties. Both fractions (blue and green) exhibited bright photoluminescence under UV illumination. The blue fraction had a higher quantum yield than the green fraction. Additionally, the blue fraction had more spectral clarity than the green fraction. The photoluminescent behavior of the nanoparticles violated both Kasha’s and Kasha-Vavilov’s rules. The carbon nanoparticles displayed excitation-dependent photoluminescence, and the photoluminescent quantum yield depends on the excitation wavelength. Adjusting the pH of the nanoparticle solutions resulted in moderate spectral changes.

Changes in the photoluminescence of the carbon nanoparticles were monitored with the addition of metal ions. Stern–Volmer plots were made to determine if photoluminescence quenching of the nanoparticles in solution occurred. Photoluminescence quenching was observed with adding Hg^2+^, Fe^3+^, Cr^3+^, Co^2+^, Ag^+^, Fe^2+^, Cu^2+^, and Ni^2+^. Most of these metal ions have partially filled d orbitals, contributing to the transfer of electrons from the photoexcited nanoparticles to the available empty orbitals of the metal ions. Hg^2+^, the lone exception in this group of metal ions, is a soft acid according to hard soft acid base theory. The thiol groups on the nanoparticle surface are considered soft bases. There was likely a strong interaction between the thiol group and Hg^2+^, resulting in a decrease in photoluminescence. The detection limits for sensing the metal ions were calculated. The lowest detection limits observed were for Hg^2+^, with values of 4.1 and 1.4 ppm using the blue and green fractions, respectively.

There are some limitations to the findings in this study. The CQDs were quenched by several metal ions, which curtails the specificity for the detection of certain metal ions. However, the significant quenching with very small concentrations of Hg^2+^ should be emphasized. Additionally, the focus of this study was on the collection of steady-state photoluminescent spectra of the CQDs; the acquisition of time-resolved photoluminescent spectra would provide addition information about the quenching mechanism. In future studies of similar carbon quantum dots, we plan to include the collection of these spectra to enhance our understanding of these nanomaterials.

## Supplementary Material



## Notes

Fluorescent spectra and Stern–Volmer plots of carbon quantum dot samples interacting with metal ion solutions (and control experiment using water), IR spectra of carbon quantum dot samples, transmission electron microscopy images and histograms, dynamic light scattering size distribution curves, quantum yield plots, absorption spectra of carbon quantum dot samples and quinine sulfate, excitation wavelength dependence plot, and synthesis scheme (PDF)

## ∥

Department of Chemistry, Mississippi State University, P.O. Box 9573, Mississippi State, Mississippi 39762-9573, United States
