# Supplementary material for: Mercury Ion Sensing Using Mercaptosuccinic Acid-Derived Carbon Quantum Dots
Source: ACS Omega. 2026 Mar 11;11(11):17746–57. doi: 10.1021/acsomega.5c12026 (PMC13019202; doi:10.1021/acsomega.5c12026)
Supplement: Supplementary file 1 [file ao5c12026_si_001.pdf]

Supporting Information for "Mercury Ion Sensing using Mercaptosuccinic Acid-derived Carbon Quantum Dots"

Authors
Haven I. Blair[(a)], Rayna E. Nemcek[(a)], Hallie G. McKinnie[(a)1], Sarah Saleh[(b)], Madison L. Walker[(b)], Justin M. Miller[(c)], and Deon T. Miles[(a)]

Affiliations
(a) Department of Chemistry, The University of the South, 735 University Avenue, Sewanee, Tennessee 37383, United States
(b) Vanderbilt University, Department of Chemistry, 7330 Stevenson Center, Station B 351822, Nashville, TN 37235, United States
(c) Middle Tennessee State University College of Basic and Applied Sciences, Chemistry, 1301 East Main Street, Murfreesboro, Tennessee, 37132, United States

Table of Contents

Description	Page
Synthesis of carbon quantum dots	2
Dependence of excitation wavelength on green MSA-CQDs	3
Infrared spectra of MSA-CQDs	4
Transmission electron microscopy (TEM) of MSA-CQDs	5
Dynamic light scattering (DLS) of MSA-CQDs	7
Absorption spectrum of quinine sulfate	9
Quantum yield plots	10
Fluorescence spectra and Stern-Volmer "plots" for control experiments using water	12
Absorption spectra of blue MSA-CQDs with added Fe^{3+}	14
Fluorescence spectra and Stern-Volmer plots for blue MSA-CQDs	15
Fluorescence spectra and Stern-Volmer plots for green MSA-CQDs	32
Stern-Volmer plots of blue MSA-CQDs with added Hg^{2+} and Fe^{3+}	49
Stern-Volmer plots of green MSA-CQDs with added Fe^{3+} and Hg^{2+}	50

[1] Department of Chemistry, P.O. Box 9573, Mississippi State, MS 39762-9573

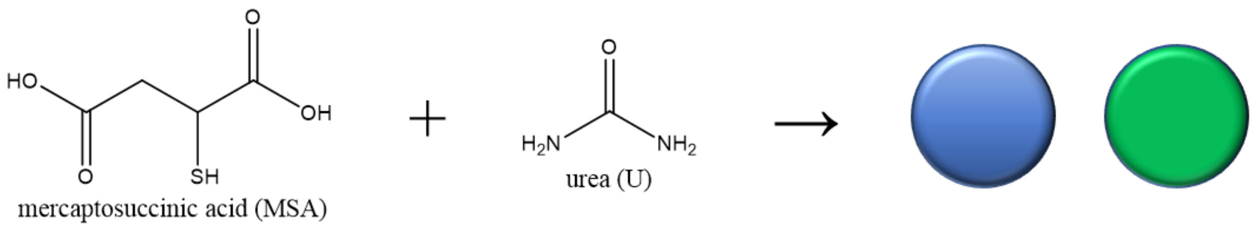

Scheme S1. Synthesis of carbon quantum dots

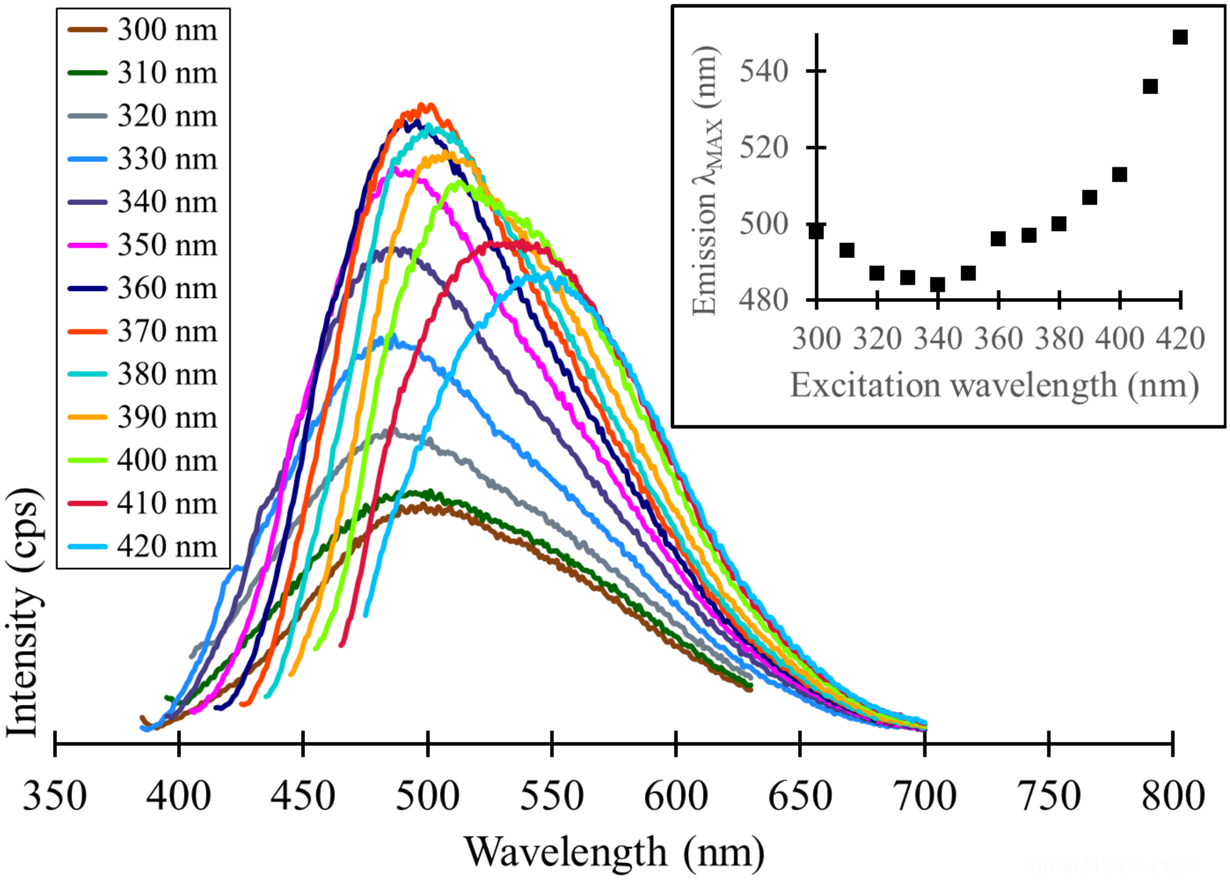

Figure S1. Steady-state fluorescence spectra of the green MSA-CQDs at various λ_{EXC} (300-420 nm). Inset is a plot of emission λ_{MAX} vs. λ_{EXC} for the green MSA-CQDs.

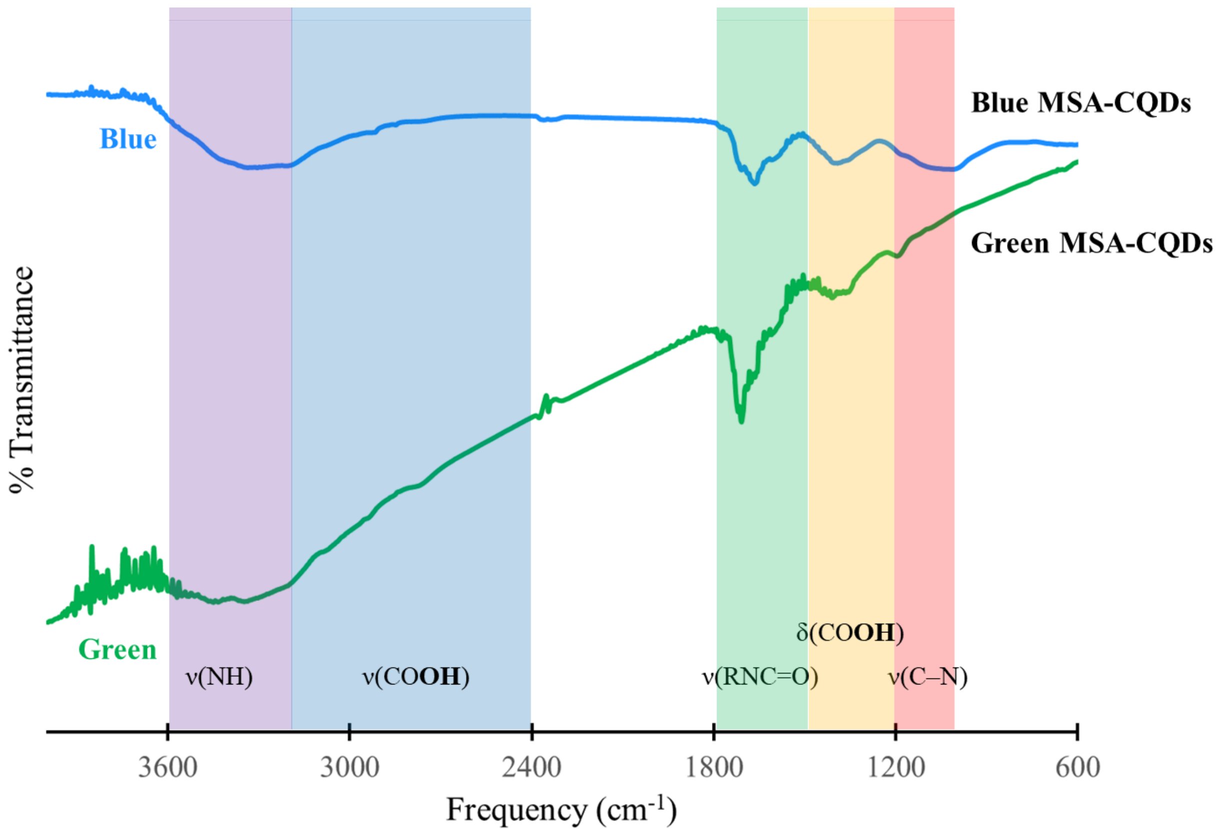

Figure S2. Infrared spectra of blue and green MSA-CQDs. From left to right, the purple band (3600-3200 cm^{-1}) is where the N–H stretching of amines and O–H stretching of alcohols is observed. The blue band (3200-2400 cm^{-1}) is where the O–H stretching of carboxylic acids is found. The green band (1800-1500 cm^{-1}) is the location to examine carbonyl (C=O) stretching from forming amide bonds. The orange band (1500-1200 cm^{-1}) is where the O–H bending of carboxylic acids is located. The red band (1200-1000 cm^{-1}) is where the C–N stretching of amines is found.

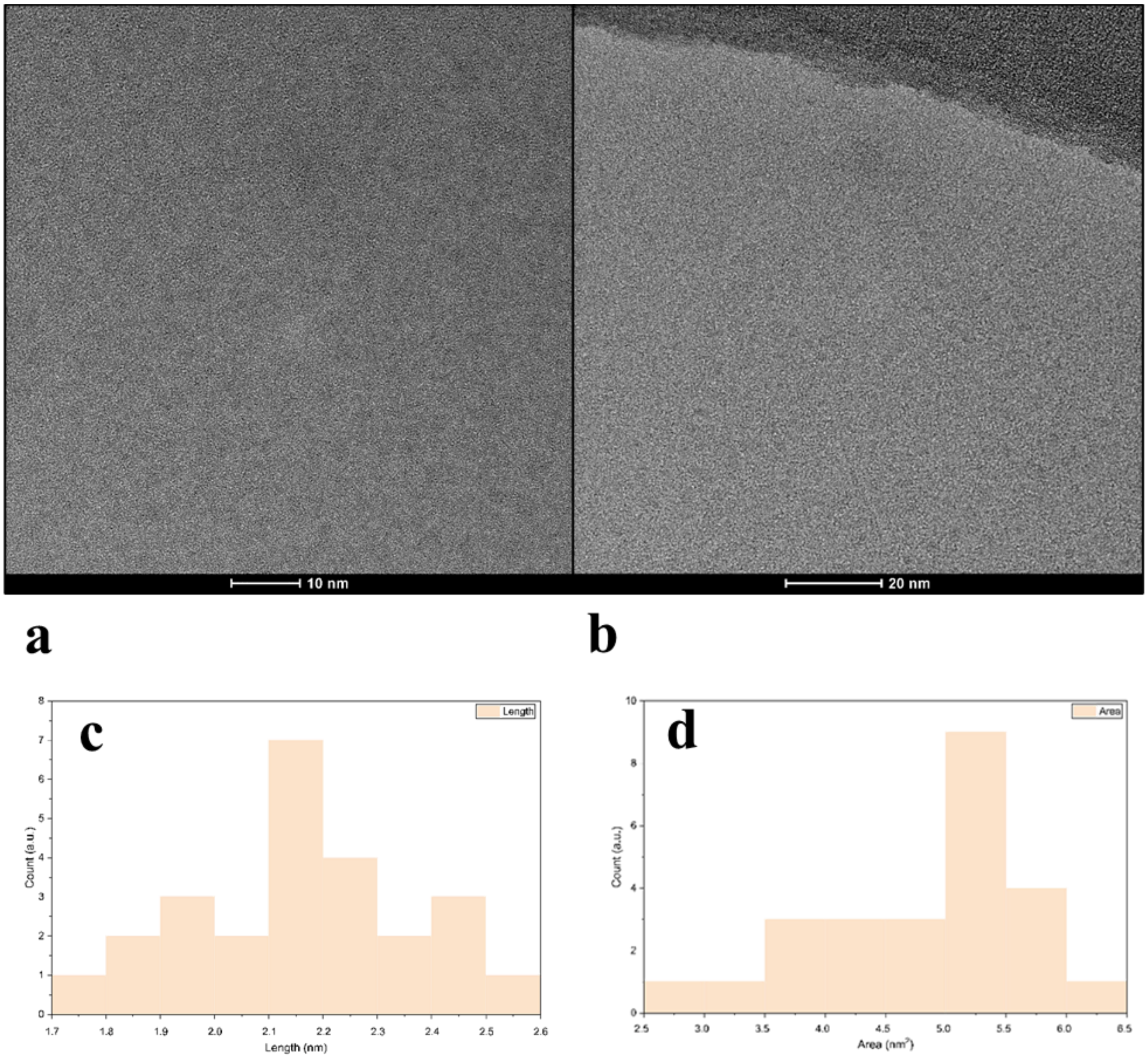

Figure S3. Blue MSA-CQDs: (a) and (b) transmission electron microscopy (TEM) images, and corresponding histograms for (c) length (diameter) and (d) area.

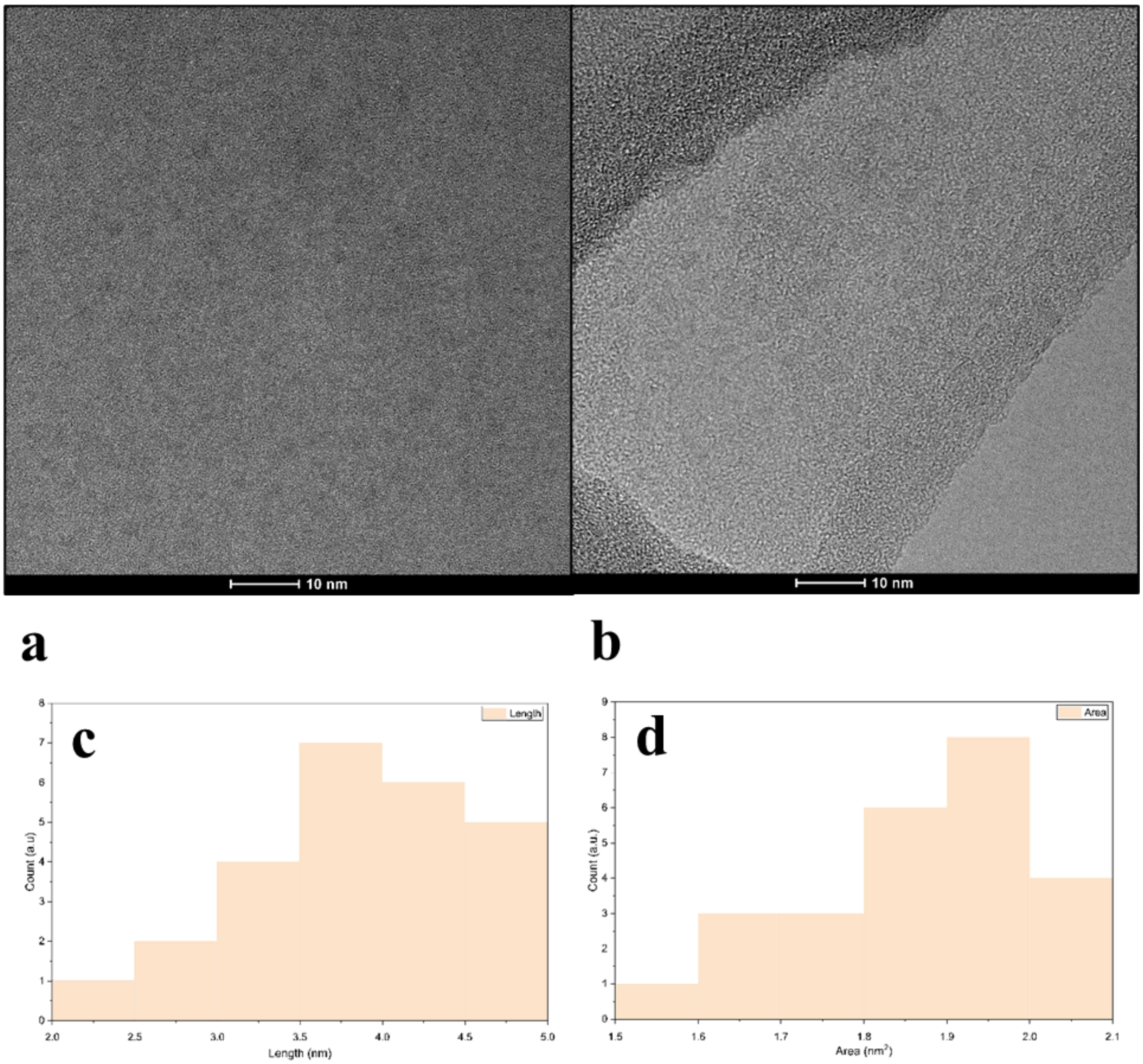

Figure S4. Green MSA-CQDs: (a) and (b) transmission electron microscopy (TEM) images, and corresponding histograms for (c) length (diameter) and (d) area.

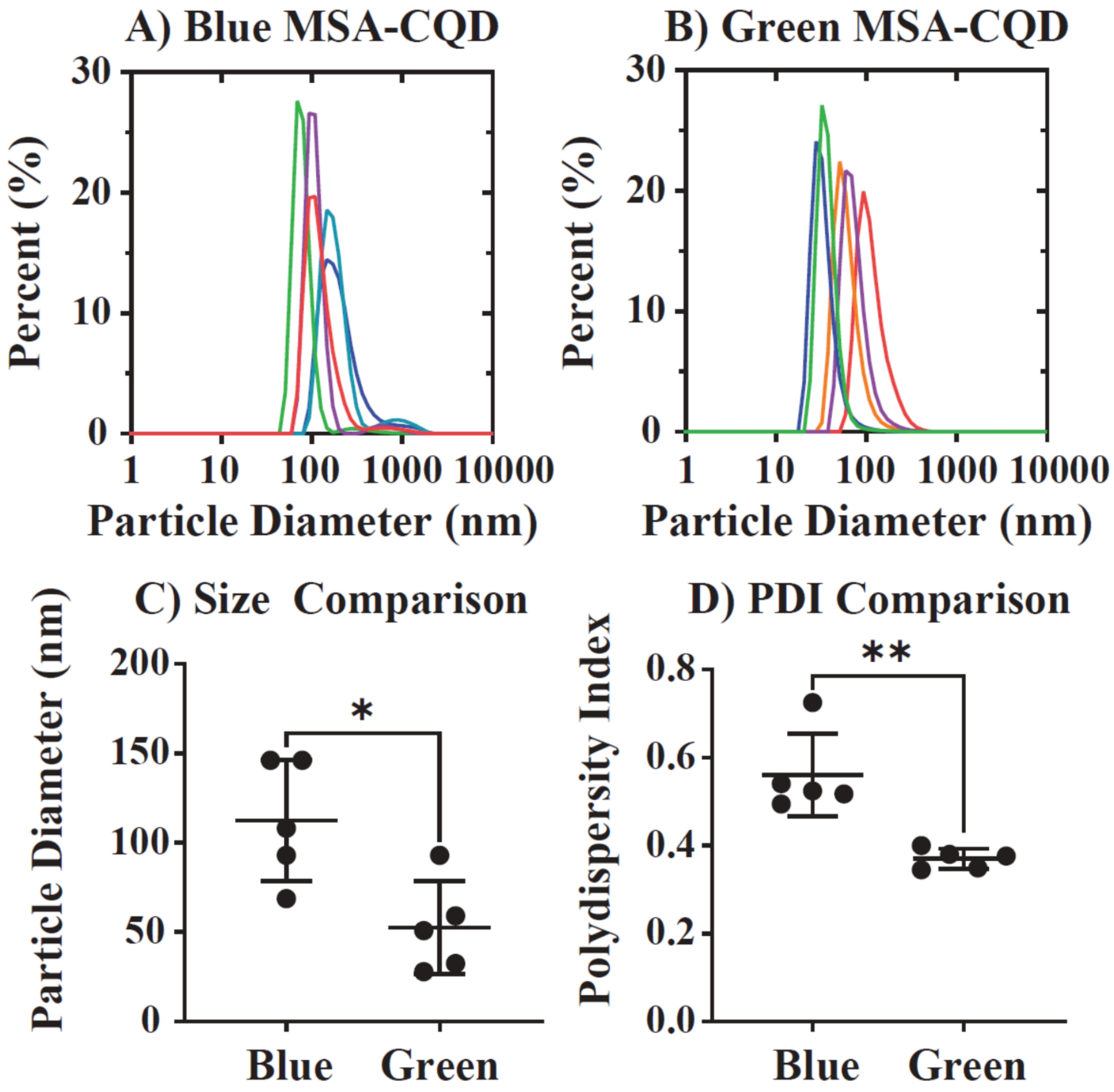

Figure S5. Dynamic light scattering (DLS) analysis: size distribution curves of (a) Blue MSA-CQDs in water and (b) Green MSA-CQDs in water; (c) size comparison of Blue and Green MSA-CQDs, and (d) polydispersity index (PDI) comparison of Blue and Green MSA-CQDs.

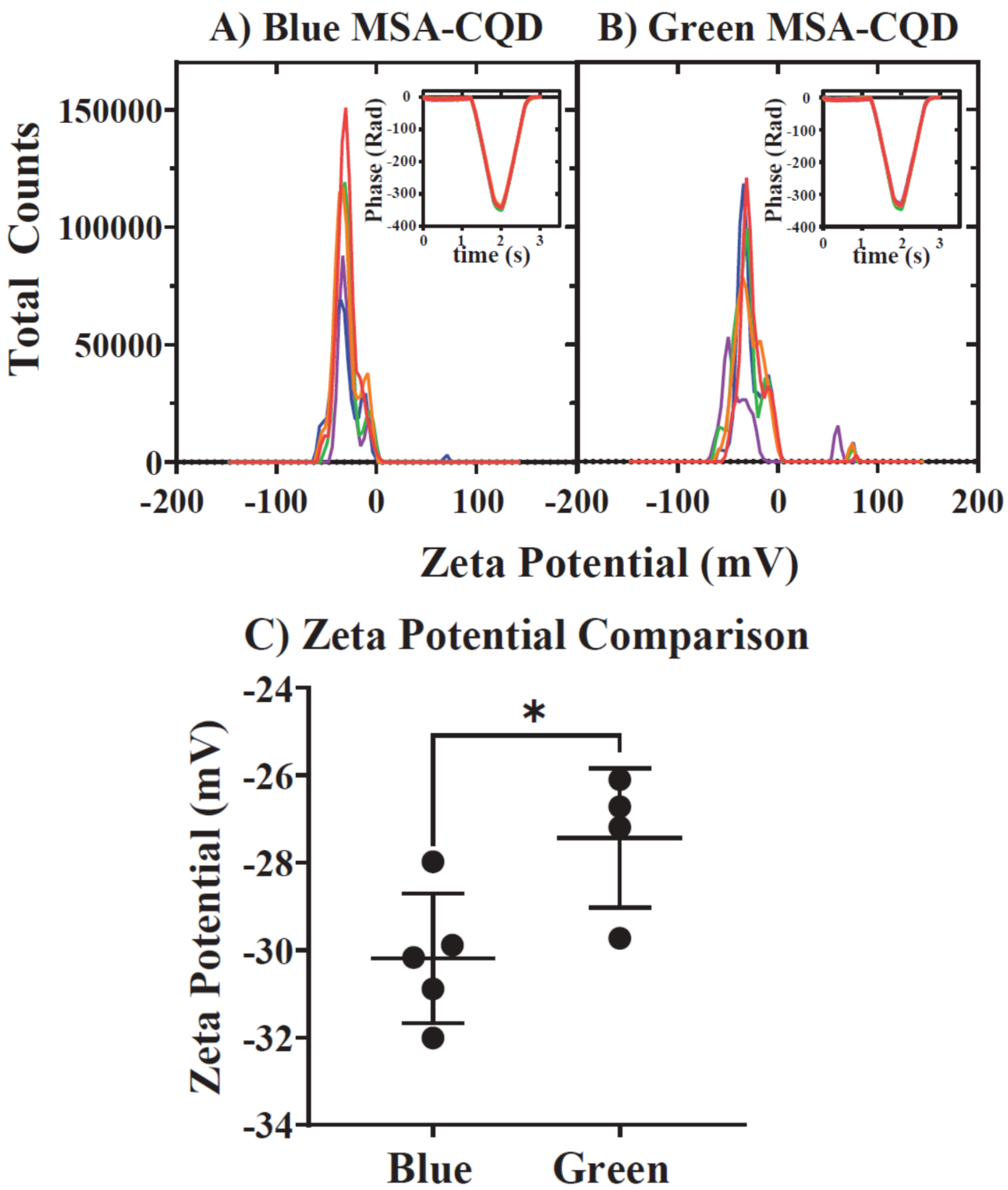

Figure S6. Zeta potential distributions of (a) Blue MSA-CQDs and (b) Green MSA-CQDs in water; and (c) comparison of Zeta potential measurements.

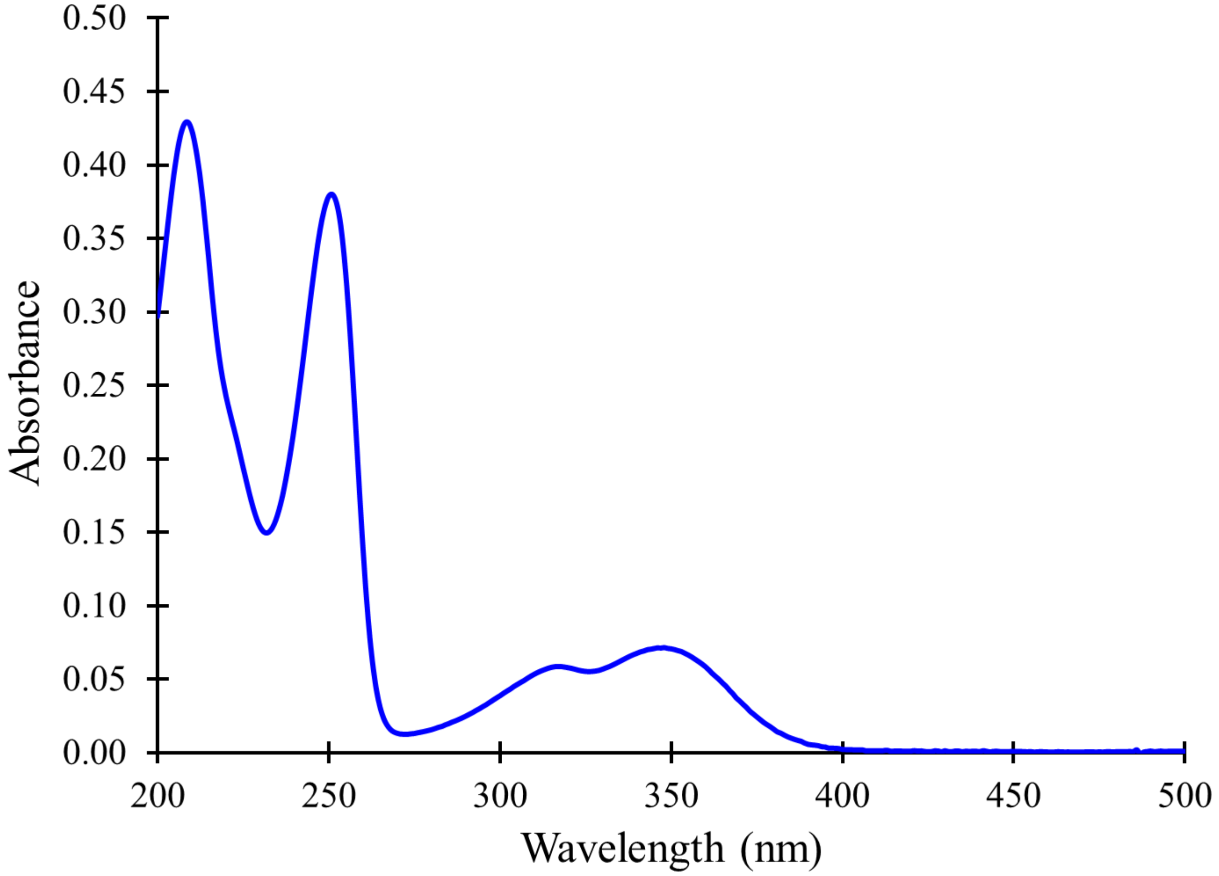

Figure S7. Absorption spectrum of quinine sulfate in 0.1 M H_2SO_4. Quinine sulfate was the reference standard for the quantum yield measurements (Φ_{QS} = 0.54).

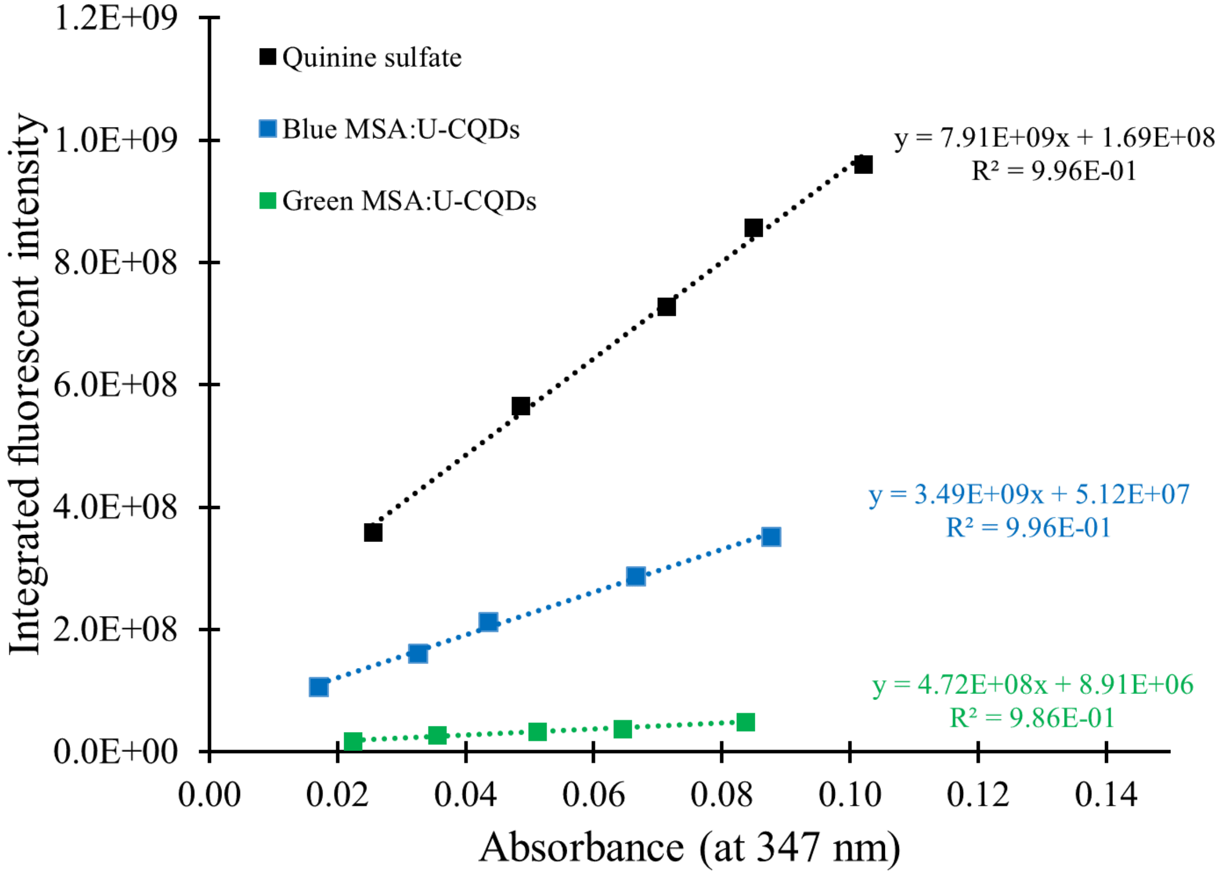

Figure S8. Quantum yield plots at λ_{EXC} = 347 nm for quinine sulfate (in 0.1 M H_2SO_4, reference Φ = 0.54), and blue and green MSA-CQDs (in water).

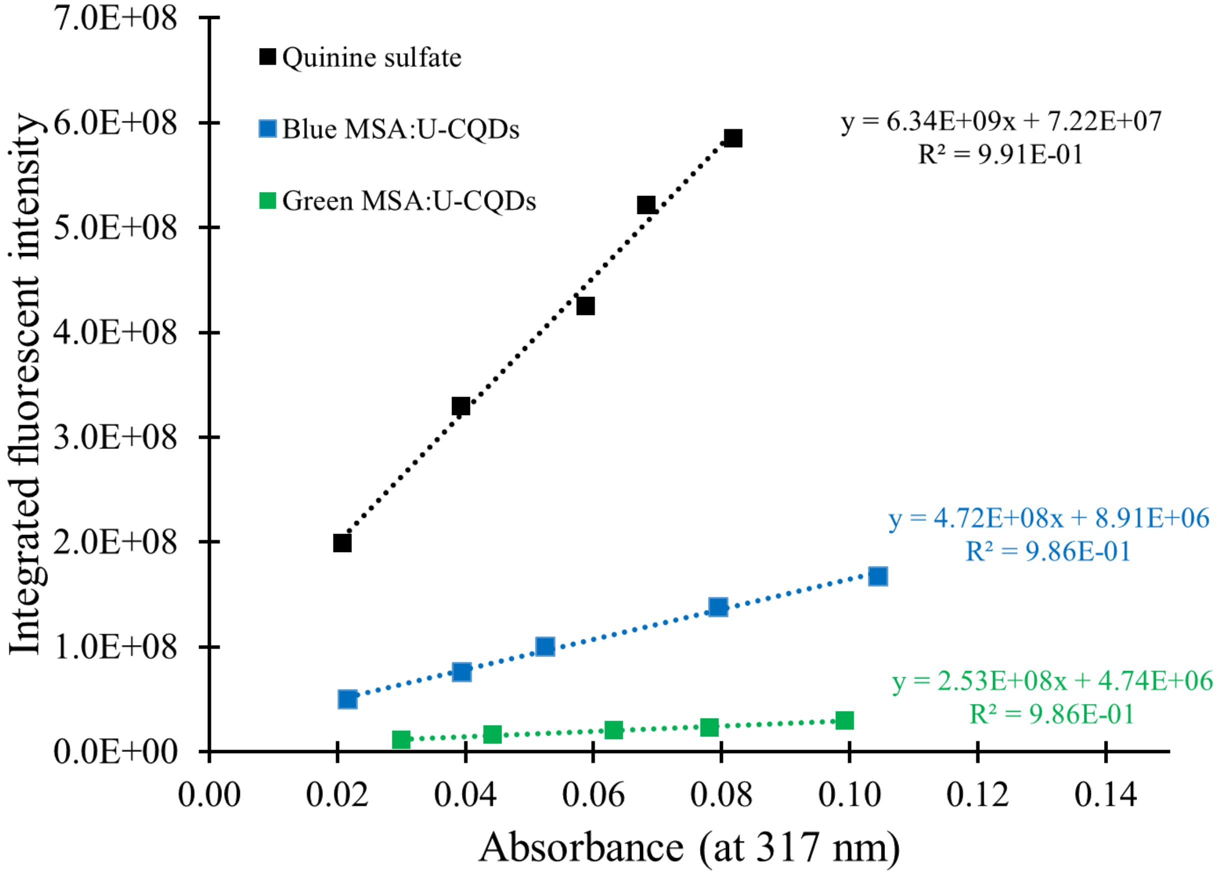

Figure S9. Quantum yield plots at λ_{EXC} = 317 nm for quinine sulfate (in 0.1 M H_2SO_4, reference Φ = 0.54), and blue and green MSA-CQDs (in water).

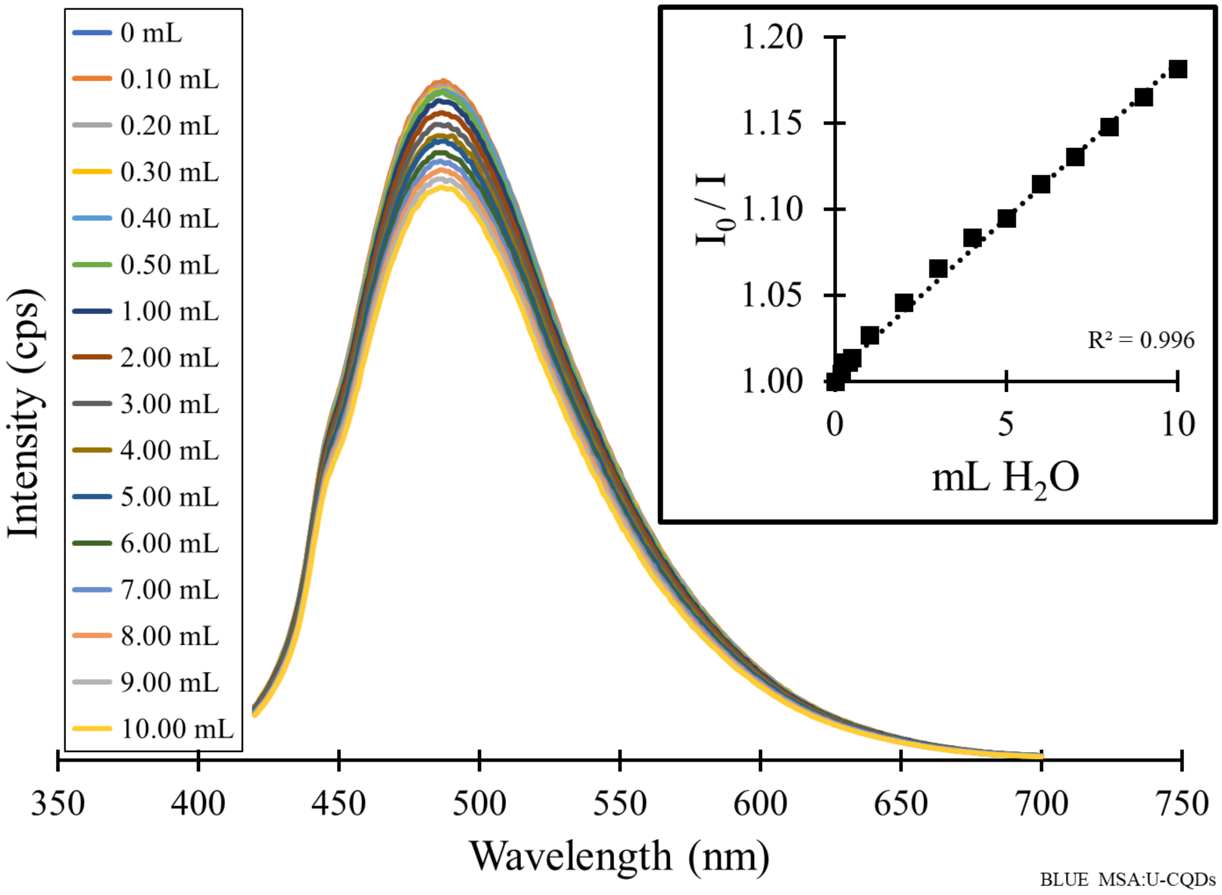

Figure S10. Steady-state fluorescence spectra (λ_{EXC} = 350 nm) of blue MSA-CQDs with added water. The added volumes of water were equal to the added metal ion solution volumes. Inset is the corresponding Stern-Volmer-like plot for this control experiment.

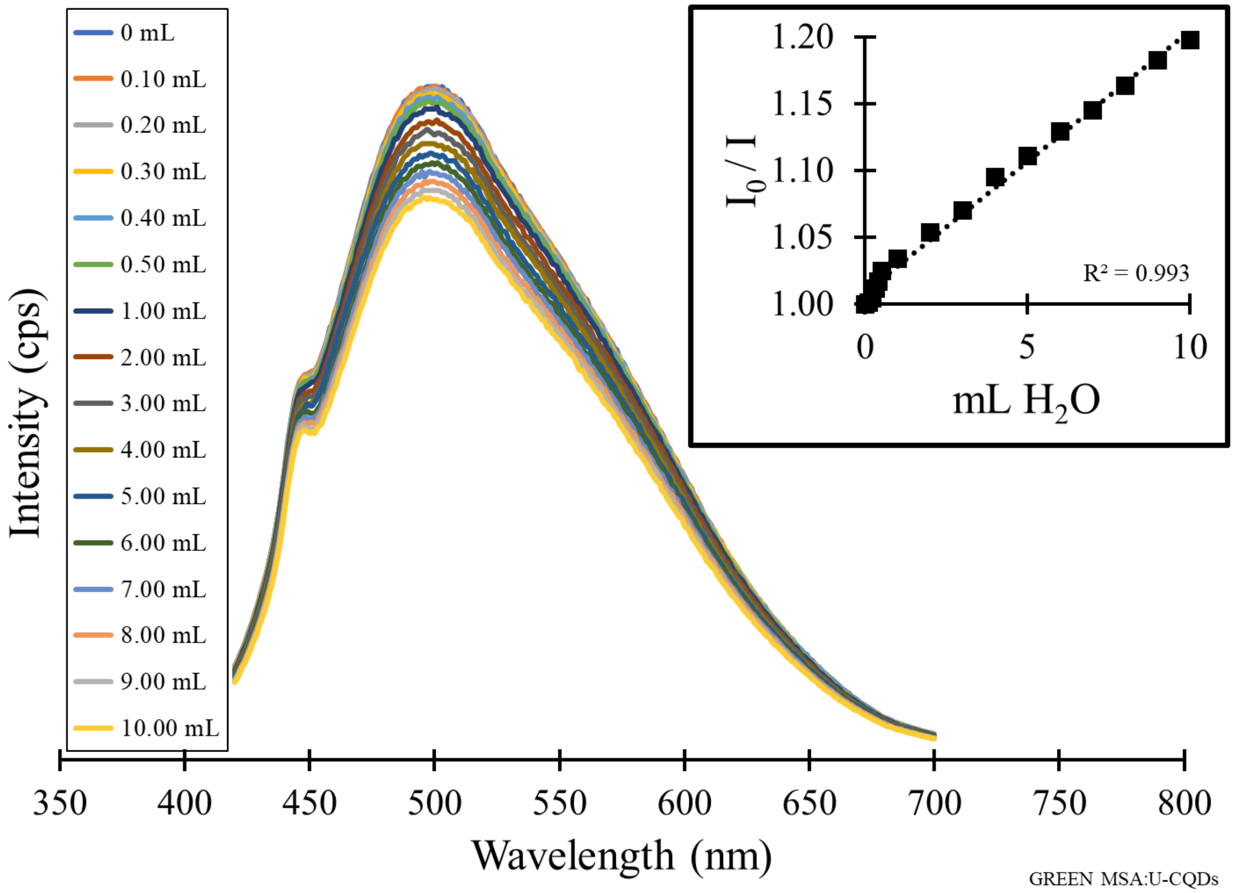

Figure S11. Steady-state fluorescence spectra (λ_{EXC} = 350 nm) of green MSA-CQDs with added water. The added volumes of water were equal to the added metal ion solution volumes. Inset is the corresponding Stern-Volmer-like plot for this control experiment.

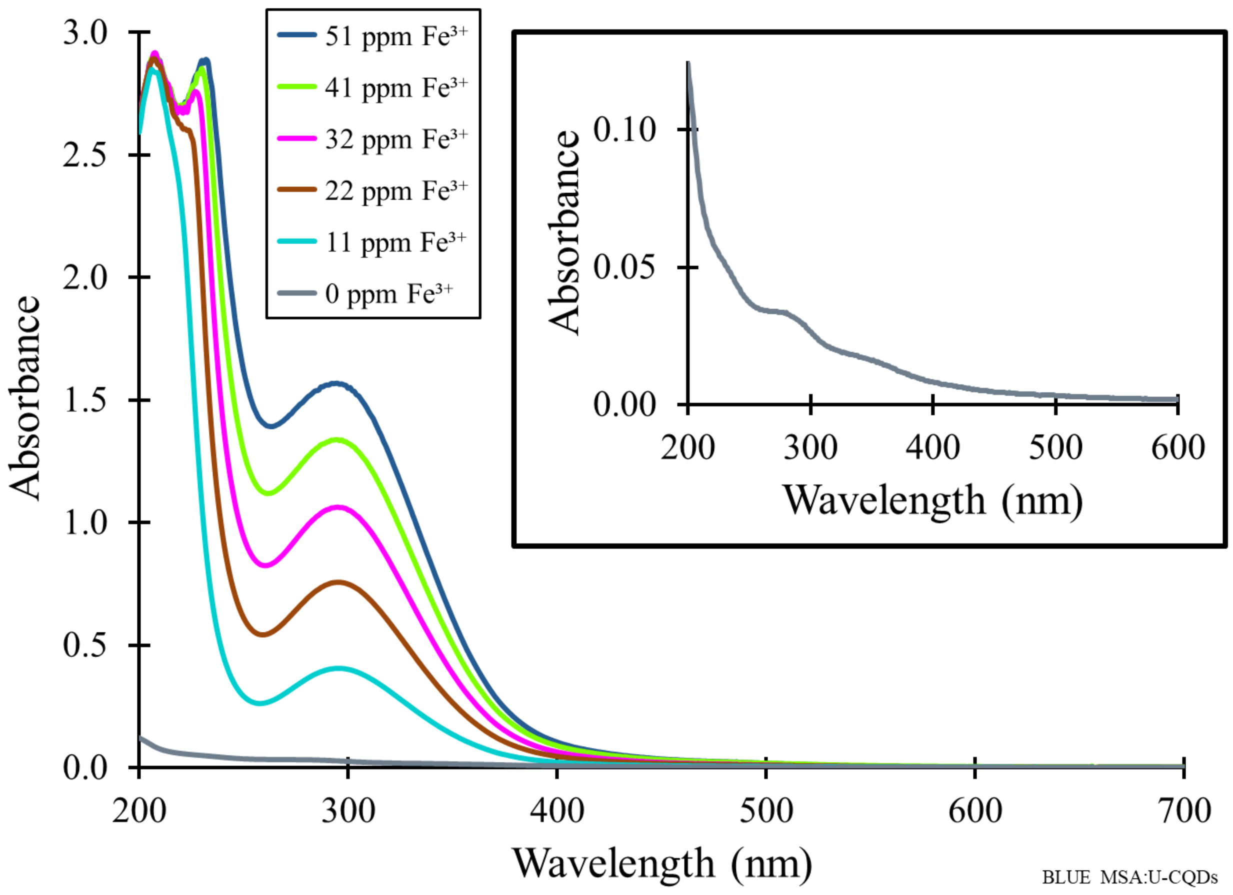

Figure S12. Absorption spectra of blue MSA-CQDs with added Fe^{3+}. Inset is the absorption spectrum of blue MSA-CQDs without added Fe^{3+} (shown for clarity).

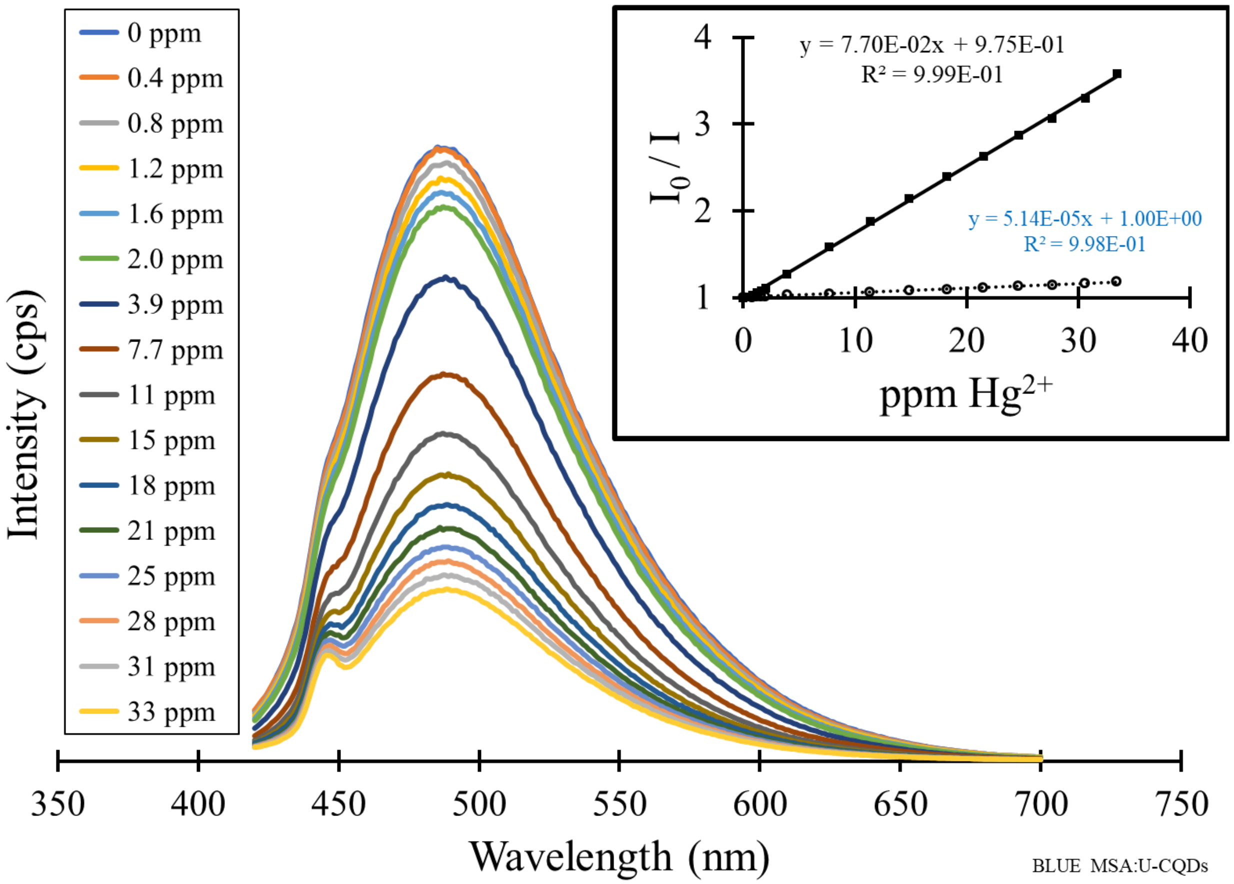

Figure S13. Steady-state fluorescence spectra (λ_{EXC} = 350 nm) of blue MSA-CQDs with added Hg^{2+}. Inset is the corresponding Stern-Volmer plot (■ = CQD interaction with Hg^{2+}, ○ = control experiment with the same volumes of water added as metal ion solution.

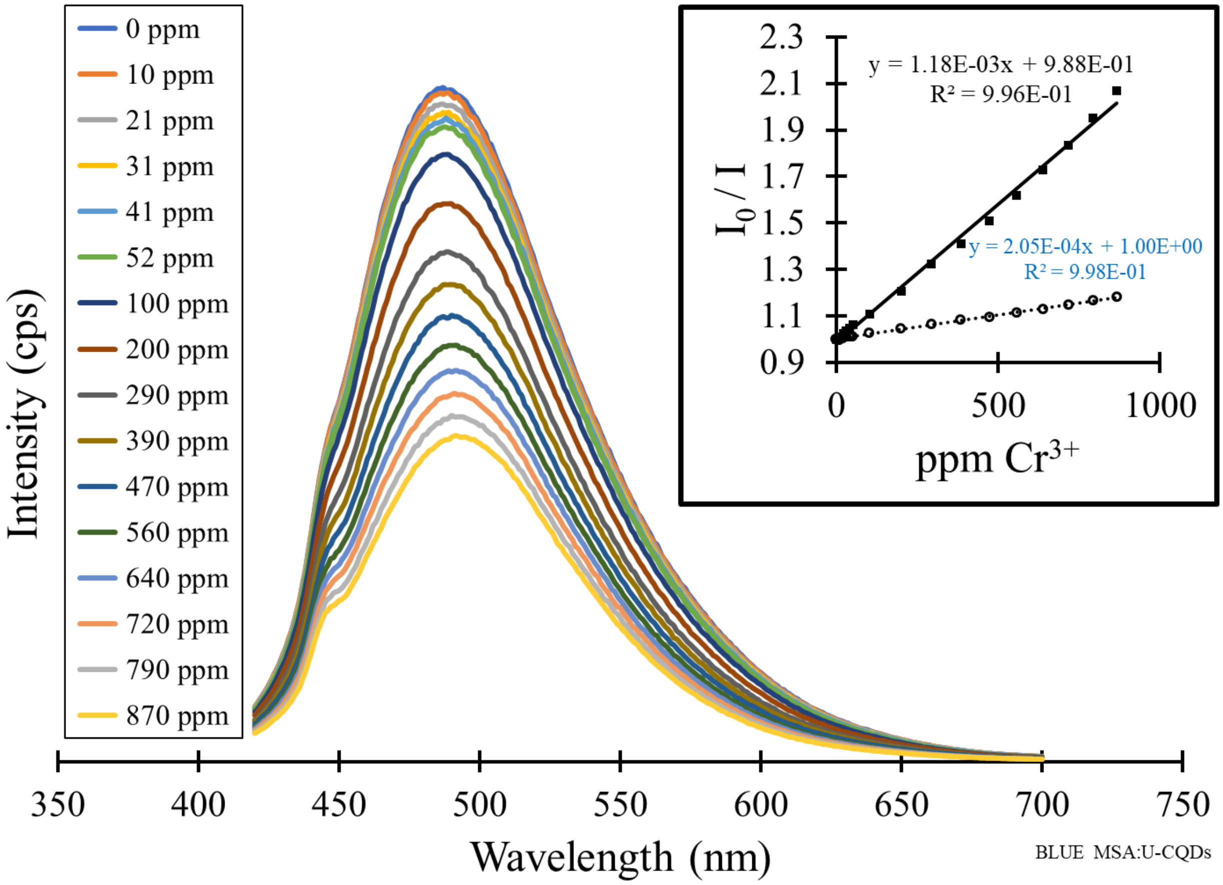

Figure S14. Steady-state fluorescence spectra (λ_{EXC} = 350 nm) of blue MSA-CQDs with added Cr^{3+}. Inset is the corresponding Stern-Volmer plot (■ = CQD interaction with Cr^{3+}, ○ = control experiment with the same volumes of water added as metal ion solution.

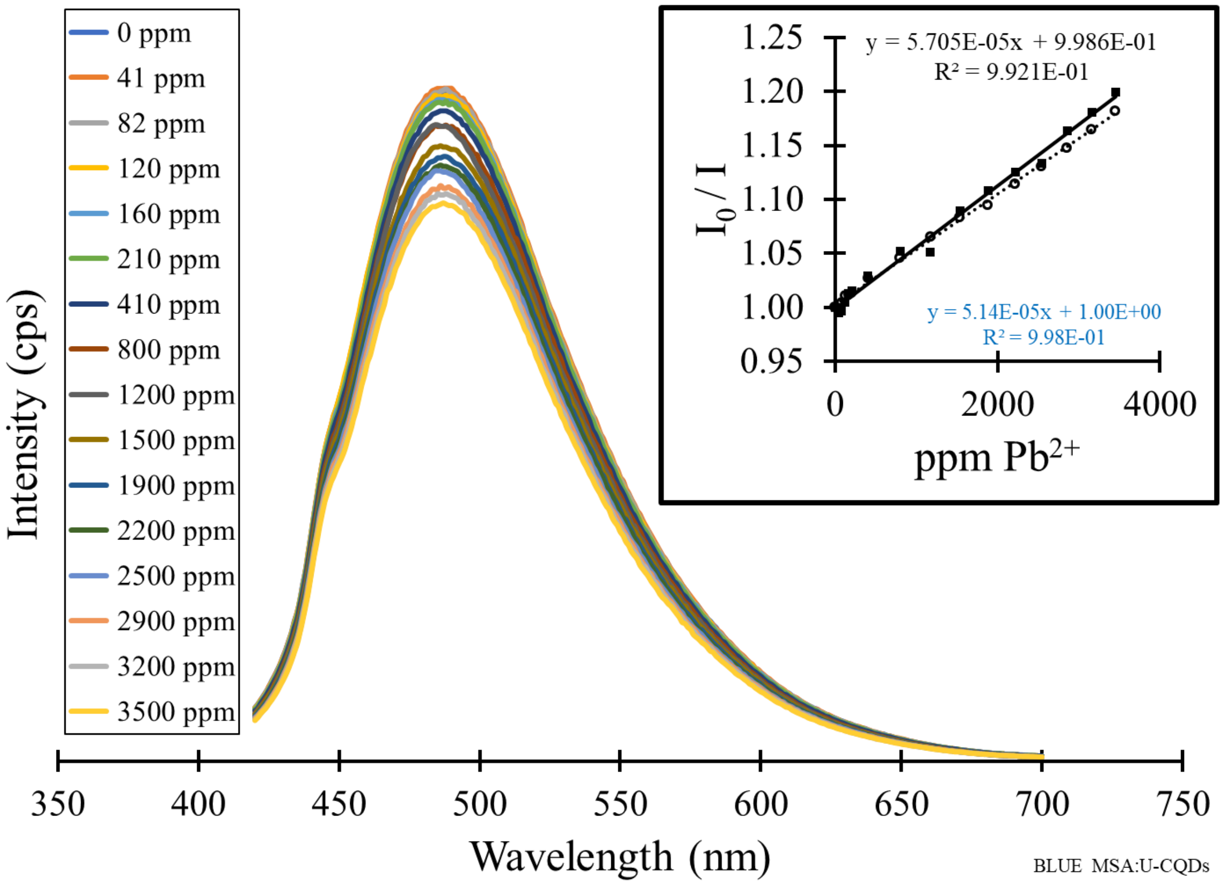

Figure S15. Steady-state fluorescence spectra (λ_{EXC} = 350 nm) of blue MSA-CQDs with added Pb^{2+}. Inset is the corresponding Stern-Volmer plot (■ = CQD interaction with Pb^{2+}, ○ = control experiment with the same volumes of water added as metal ion solution.

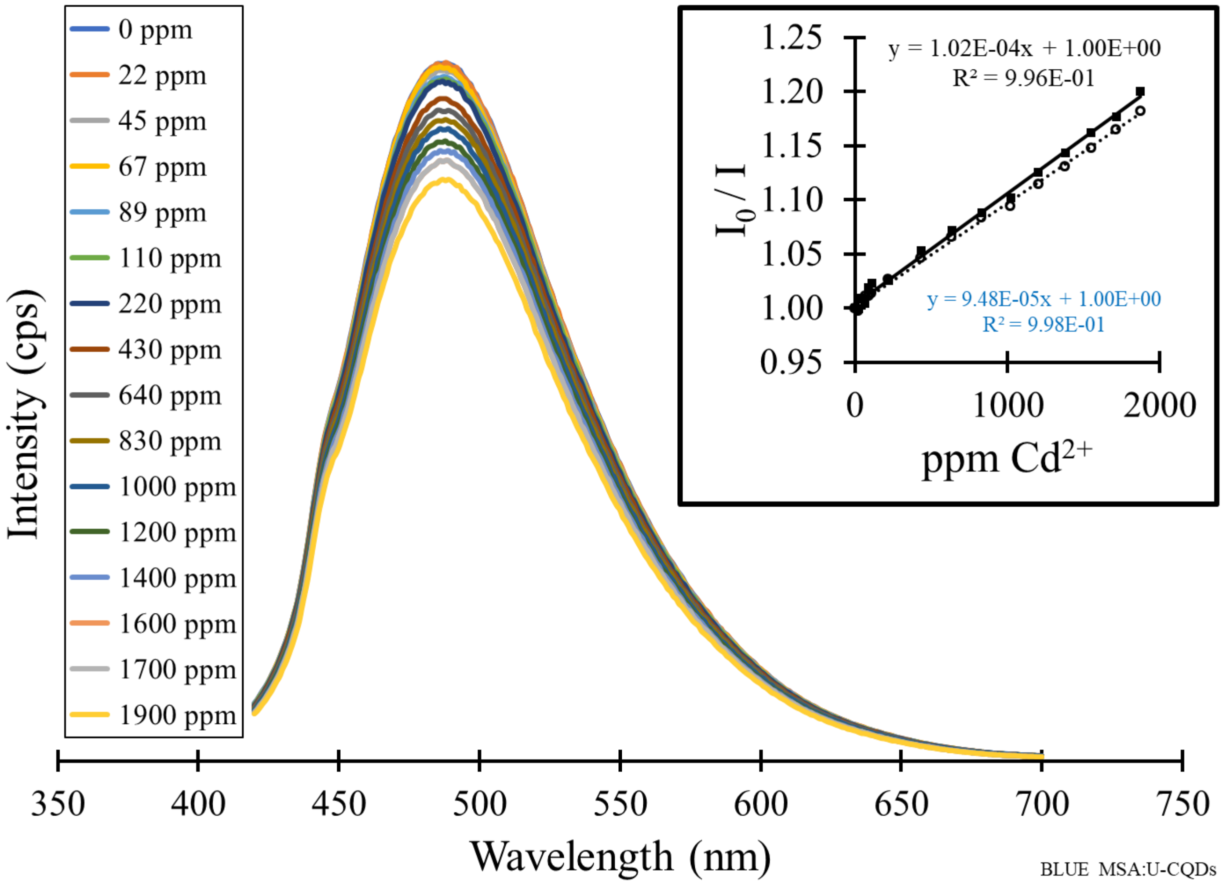

Figure S16. Steady-state fluorescence spectra (λ_{EXC} = 350 nm) of blue MSA-CQDs with added Cd^{2+}. Inset is the corresponding Stern-Volmer plot (■ = CQD interaction with Cd^{2+}, ○ = control experiment with the same volumes of water added as metal ion solution.

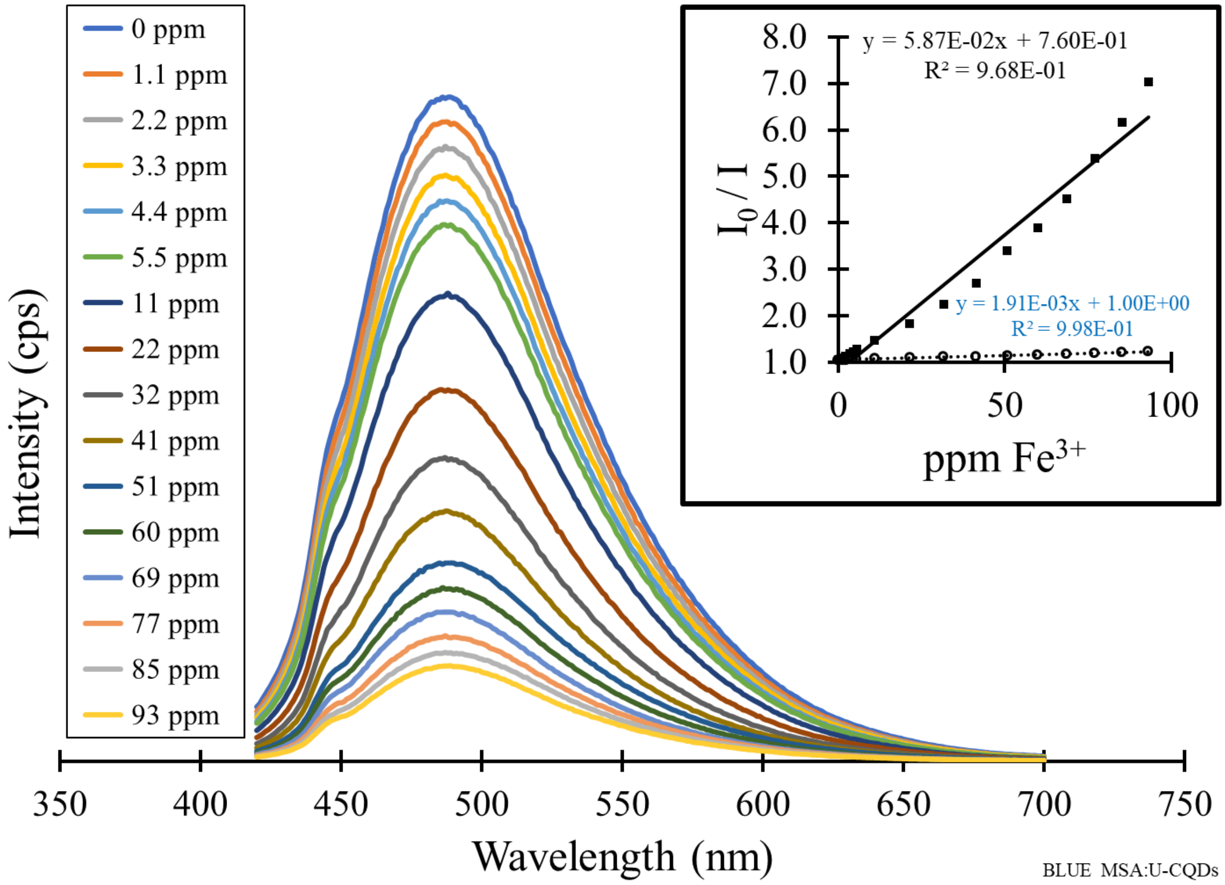

Figure S17. Steady-state fluorescence spectra (λ_{EXC} = 350 nm) of blue MSA-CQDs with added Fe^{3+}. Inset is the corresponding Stern-Volmer plot (■ = CQD interaction with Fe^{3+}, ○ = control experiment with the same volumes of water added as metal ion solution.

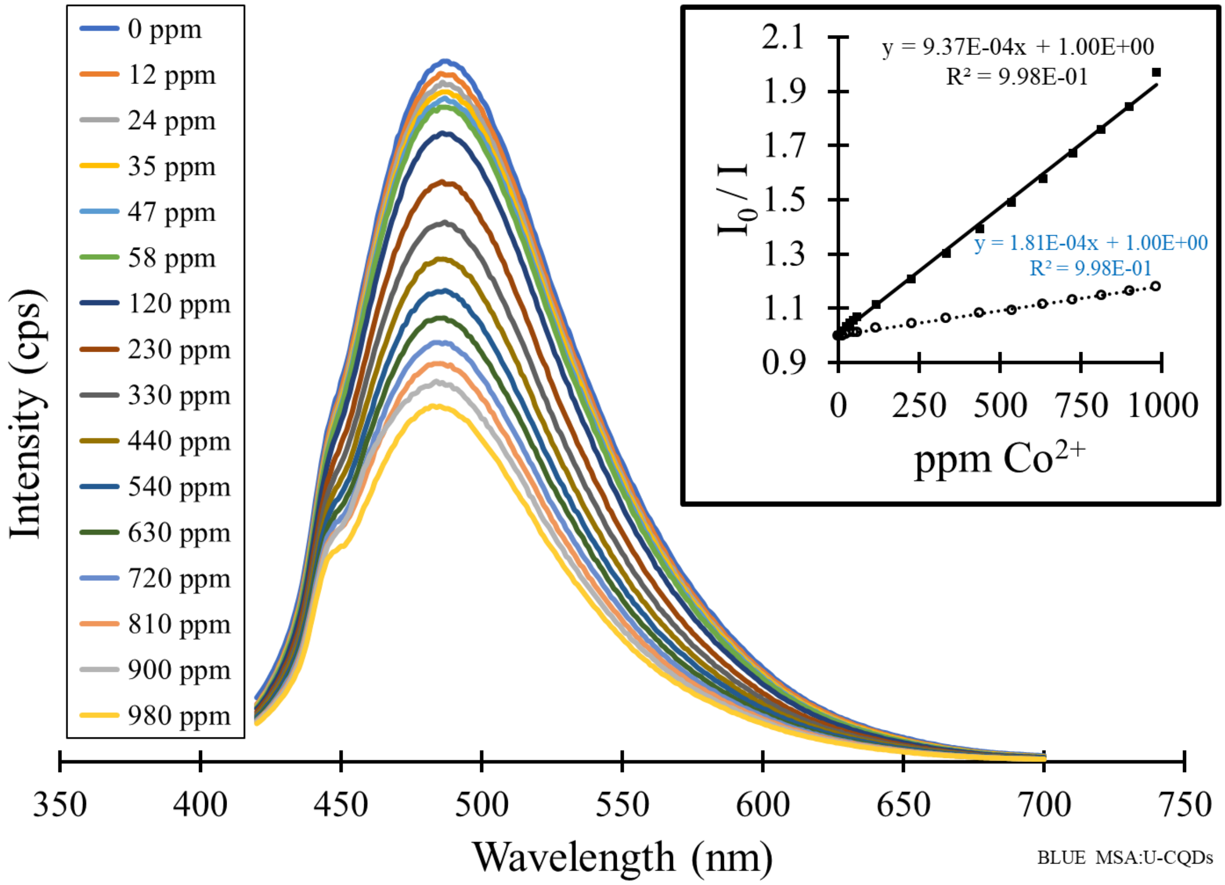

Figure S18. Steady-state fluorescence spectra (λ_{EXC} = 350 nm) of blue MSA-CQDs with added Co^{2+}. Inset is the corresponding Stern-Volmer plot (■ = CQD interaction with Co^{2+}, ○ = control experiment with the same volumes of water added as metal ion solution.

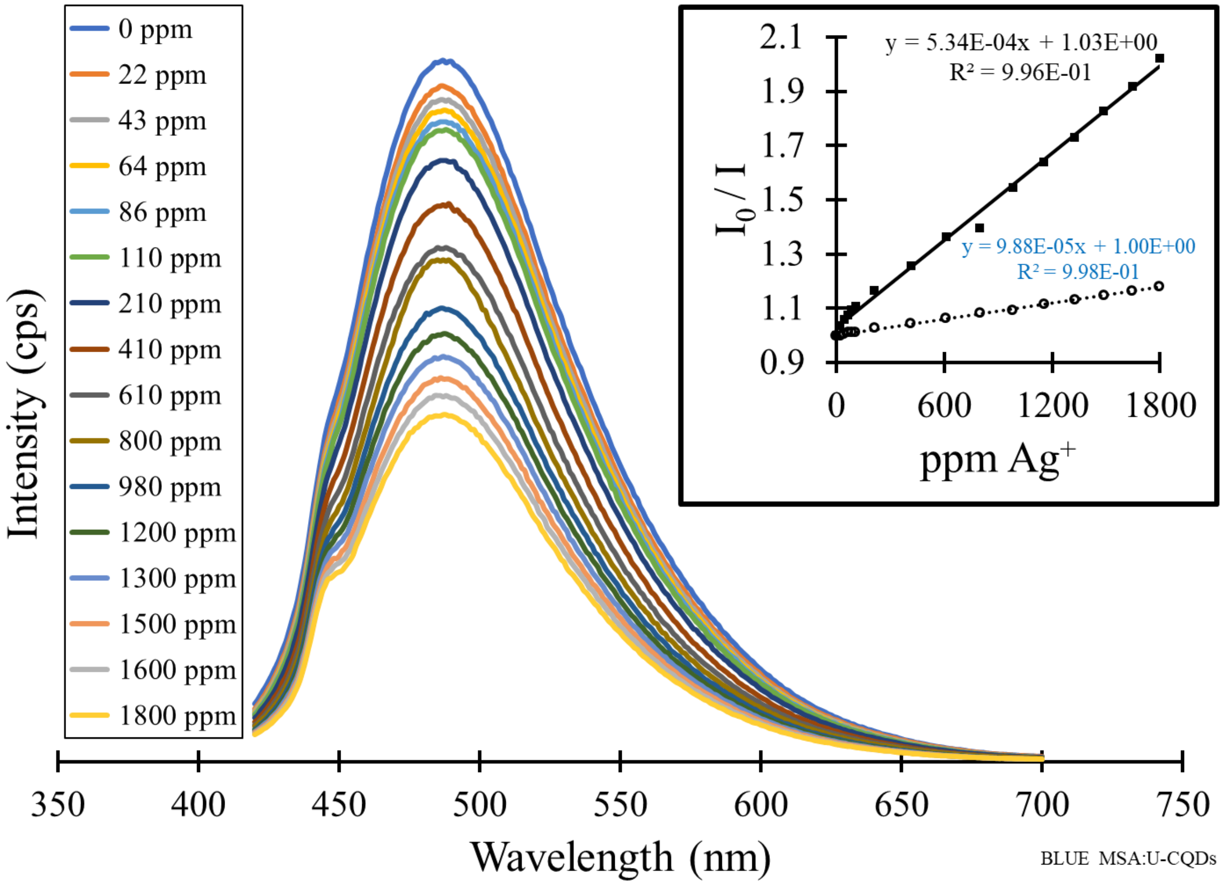

Figure S19. Steady-state fluorescence spectra (λ_{EXC} = 350 nm) of blue MSA-CQDs with added Ag^{+}. Inset is the corresponding Stern-Volmer plot (■ = CQD interaction with Ag^{+}, ○ = control experiment with the same volumes of water added as metal ion solution.

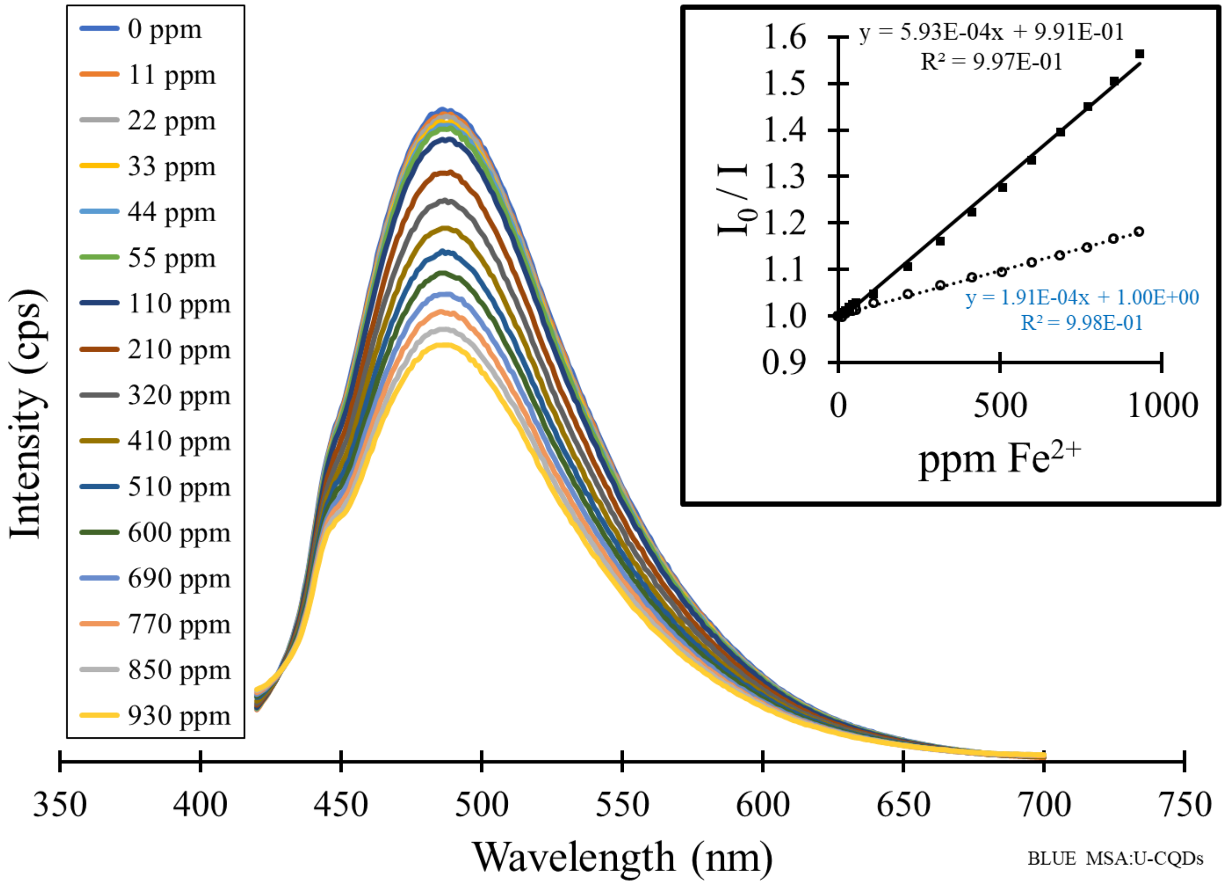

Figure S20. Steady-state fluorescence spectra (λ_{EXC} = 350 nm) of blue MSA-CQDs with added Fe^{2+}. Inset is the corresponding Stern-Volmer plot (■ = CQD interaction with Fe^{2+}, ○ = control experiment with the same volumes of water added as metal ion solution.

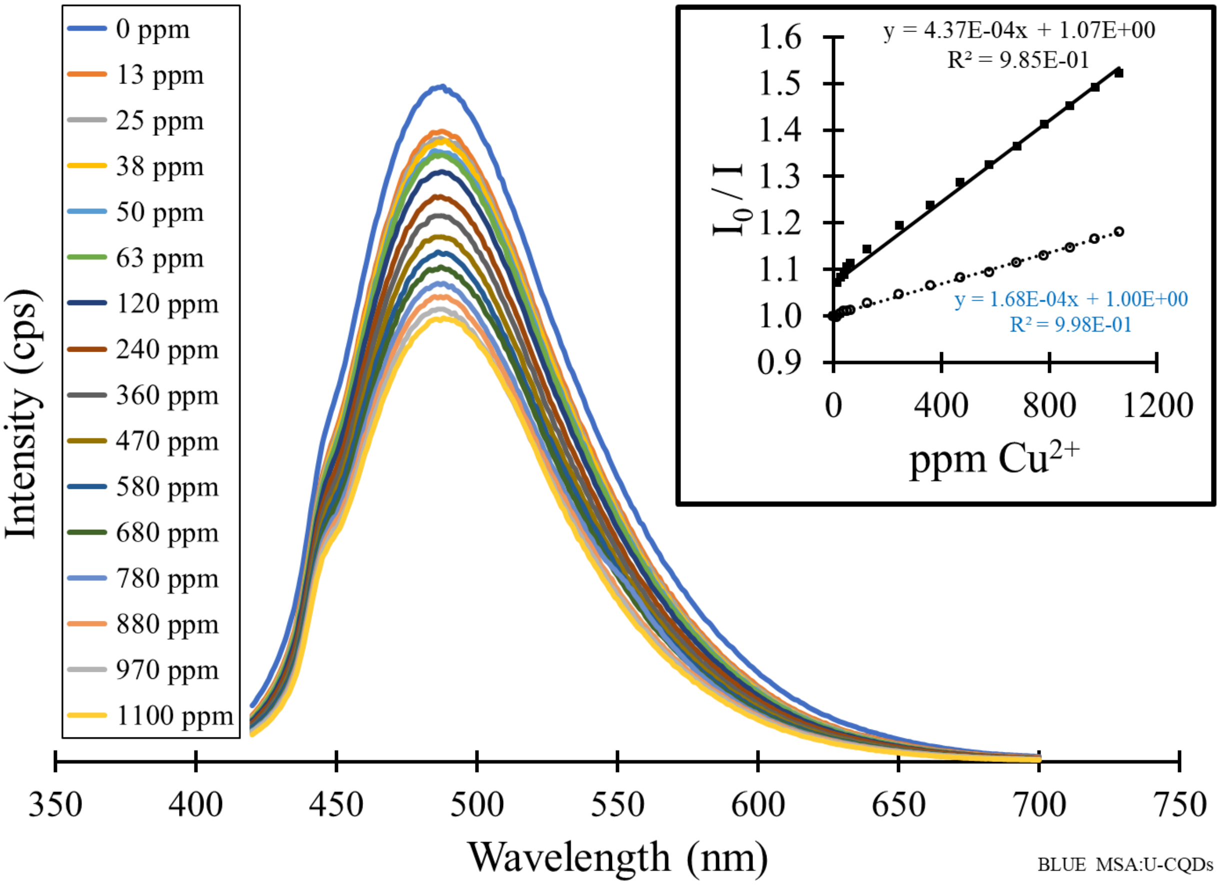

Figure S21. Steady-state fluorescence spectra (λ_{EXC} = 350 nm) of blue MSA-CQDs with added Cu^{2+}. Inset is the corresponding Stern-Volmer plot (■ = CQD interaction with Cu^{2+}, ○ = control experiment with the same volumes of water added as metal ion solution.

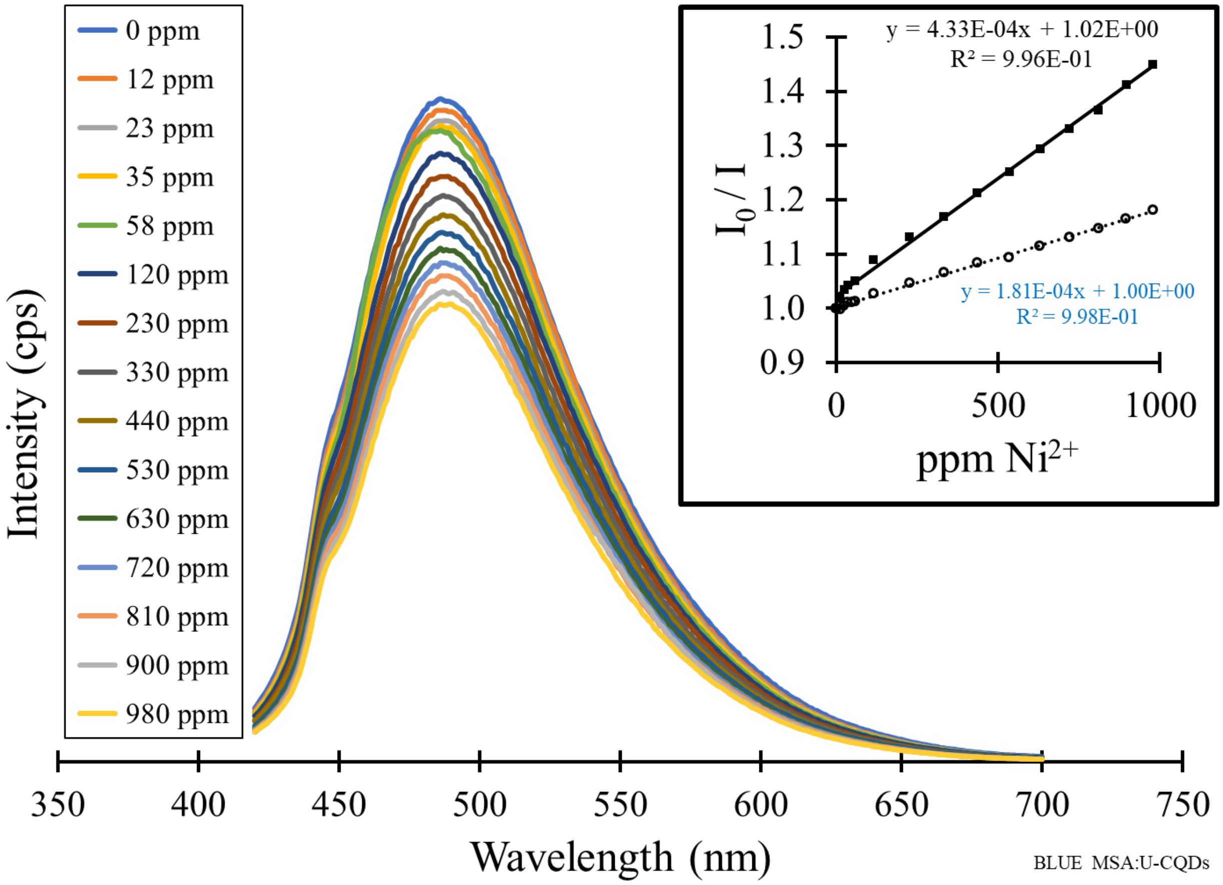

Figure S22. Steady-state fluorescence spectra (λ_{EXC} = 350 nm) of blue MSA-CQDs with added Ni^{2+}. Inset is the corresponding Stern-Volmer plot (■ = CQD interaction with Ni^{2+}, ○ = control experiment with the same volumes of water added as metal ion solution.

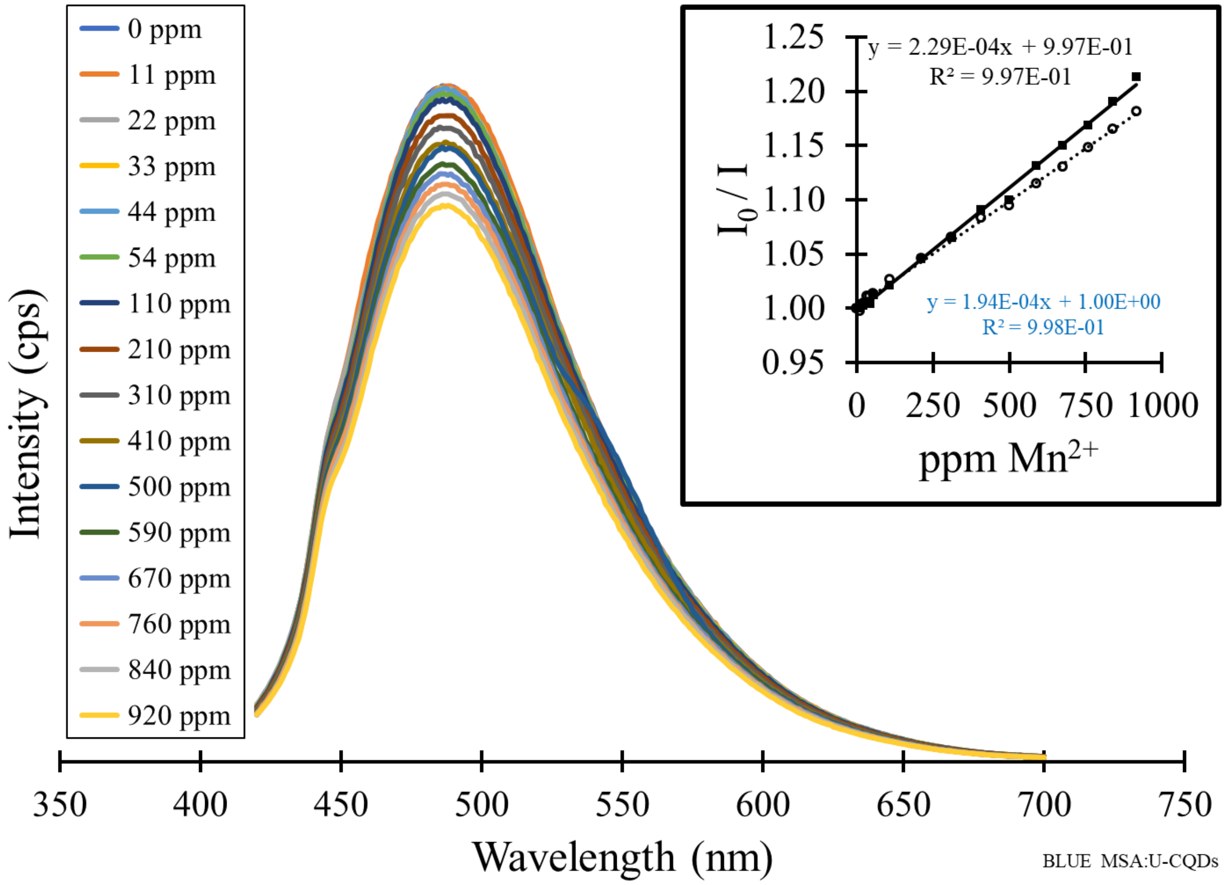

Figure S23. Steady-state fluorescence spectra (λ_{EXC} = 350 nm) of blue MSA-CQDs with added Mn^{2+}. Inset is the corresponding Stern-Volmer plot (■ = CQD interaction with Mn^{2+}, ○ = control experiment with the same volumes of water added as metal ion solution.

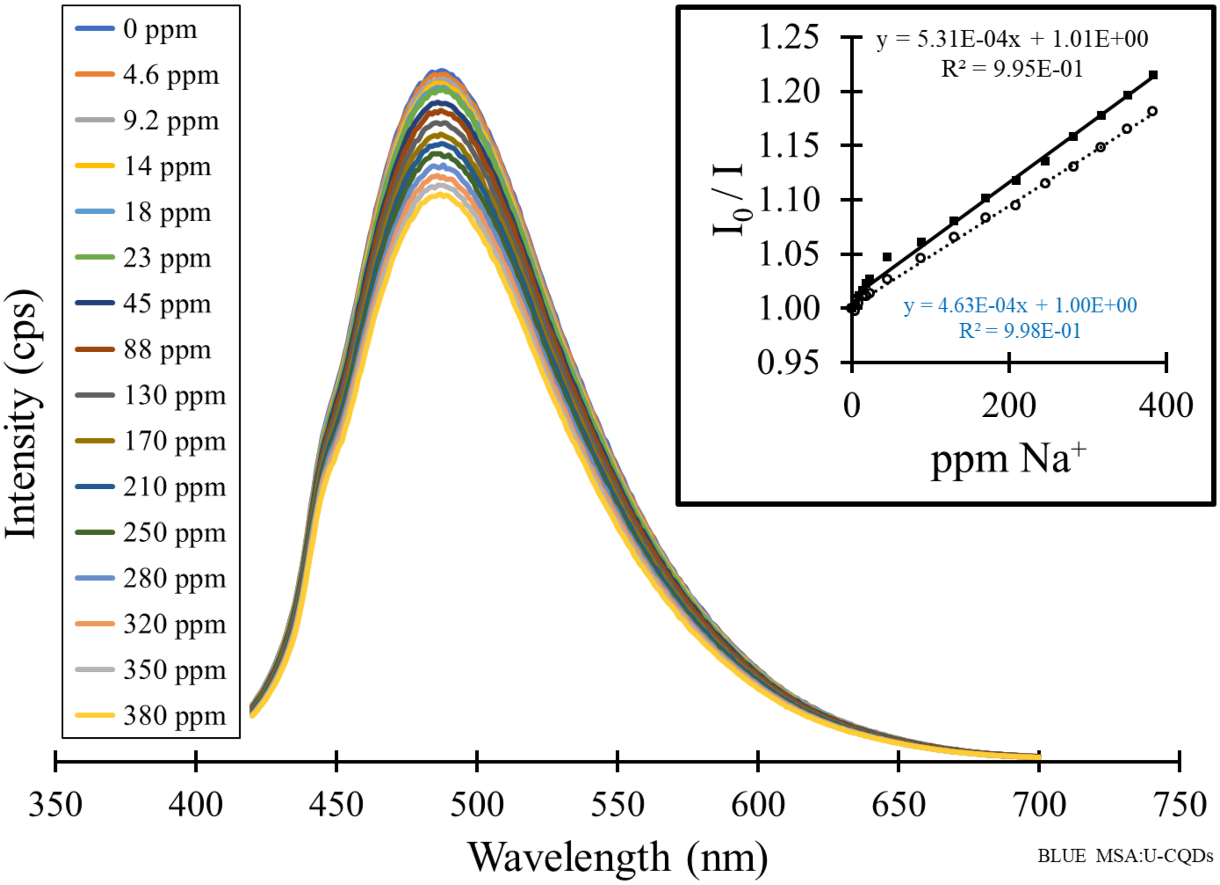

Figure S24. Steady-state fluorescence spectra (λ_{EXC} = 350 nm) of blue MSA-CQDs with added Na^+. Inset is the corresponding Stern-Volmer plot (■ = CQD interaction with Na^+, ○ = control experiment with the same volumes of water added as metal ion solution.

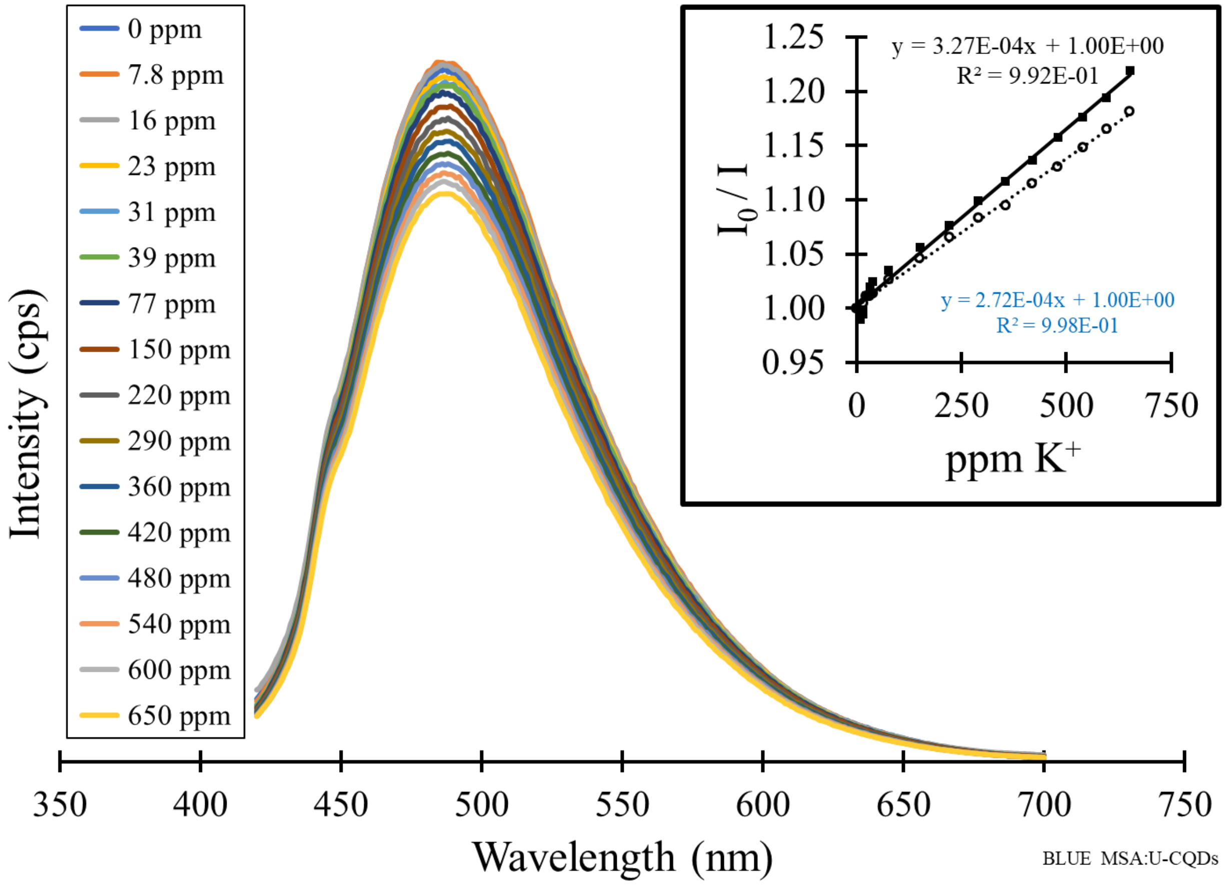

Figure S25. Steady-state fluorescence spectra (λ_{EXC} = 350 nm) of blue MSA-CQDs with added K^+. Inset is the corresponding Stern-Volmer plot (■ = CQD interaction with K^+, ○ = control experiment with the same volumes of water added as metal ion solution.

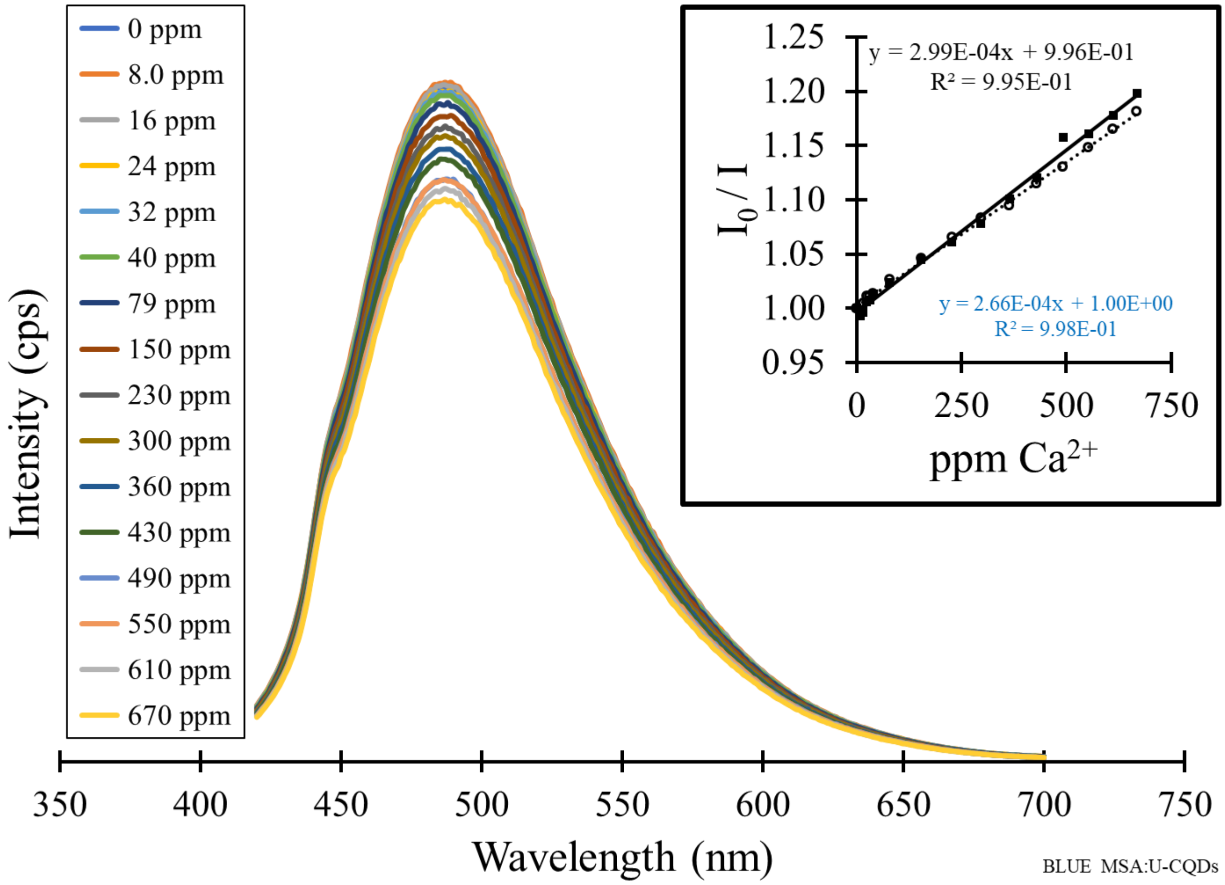

Figure S26. Steady-state fluorescence spectra (λ_{EXC} = 350 nm) of blue MSA-CQDs with added Ca^{2+}. Inset is the corresponding Stern-Volmer plot (■ = CQD interaction with Ca^{2+}, ○ = control experiment with the same volumes of water added as metal ion solution.

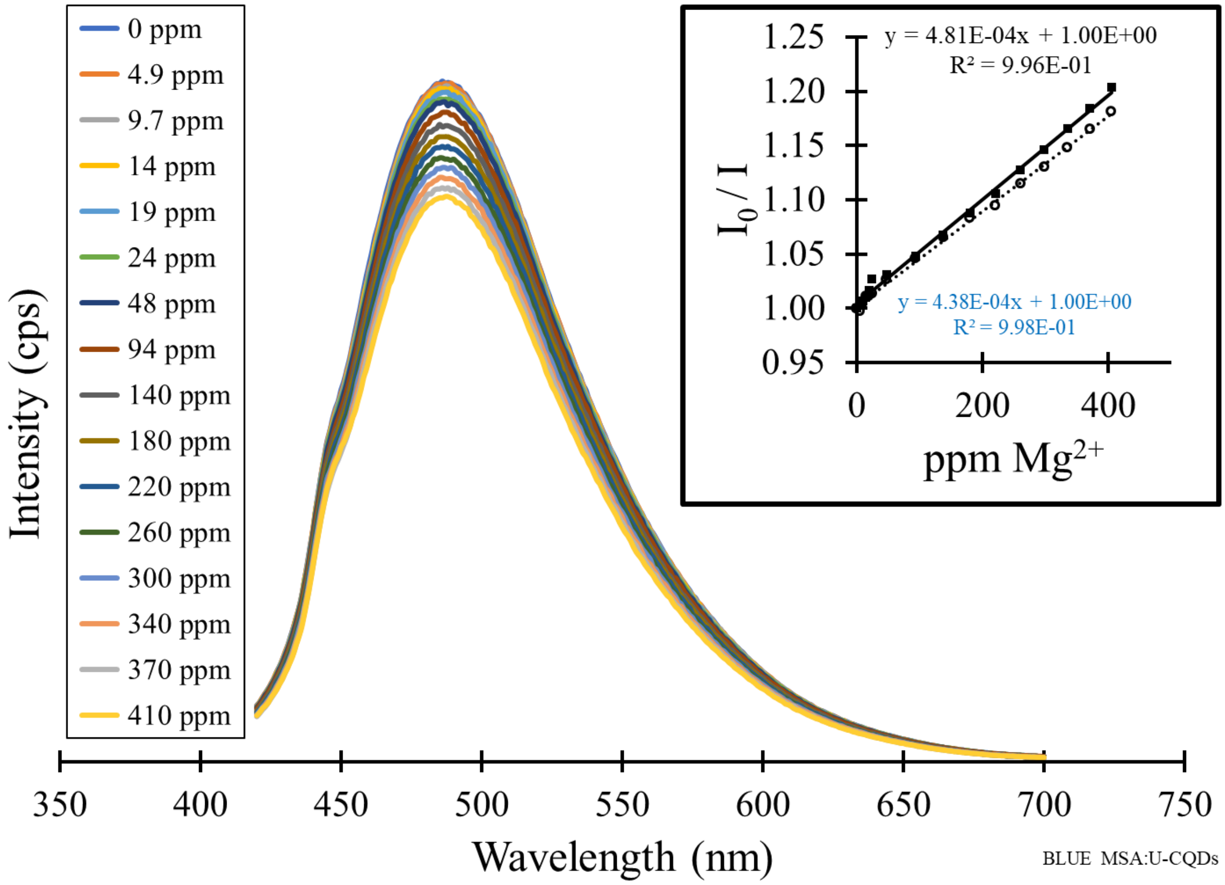

Figure S27. Steady-state fluorescence spectra (λ_{EXC} = 350 nm) of blue MSA-CQDs with added Mg^{2+}. Inset is the corresponding Stern-Volmer plot (■ = CQD interaction with Mg^{2+}, ○ = control experiment with the same volumes of water added as metal ion solution.

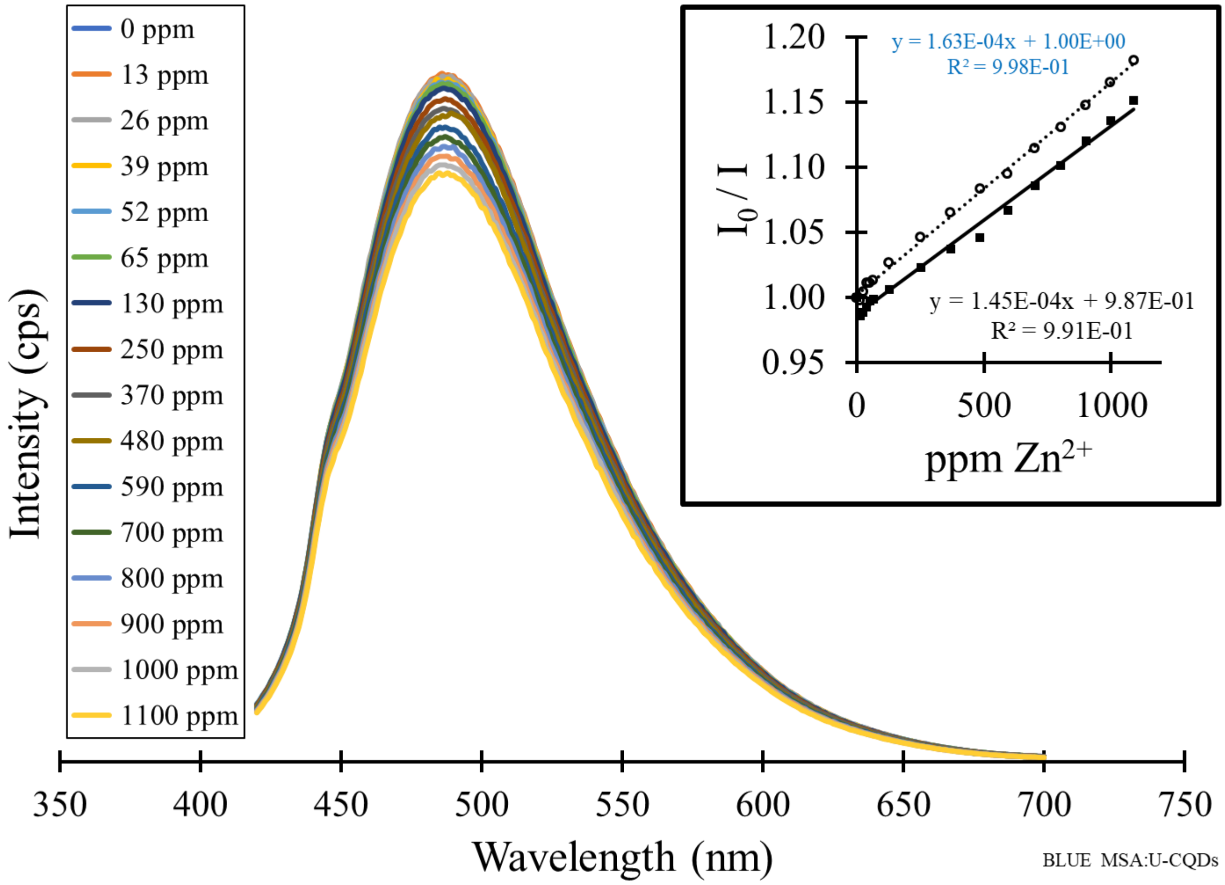

Figure S28. Steady-state fluorescence spectra (λ_{EXC} = 350 nm) of blue MSA-CQDs with added Zn^{2+}. Inset is the corresponding Stern-Volmer plot (■ = CQD interaction with Zn^{2+}, ○ = control experiment with the same volumes of water added as metal ion solution.

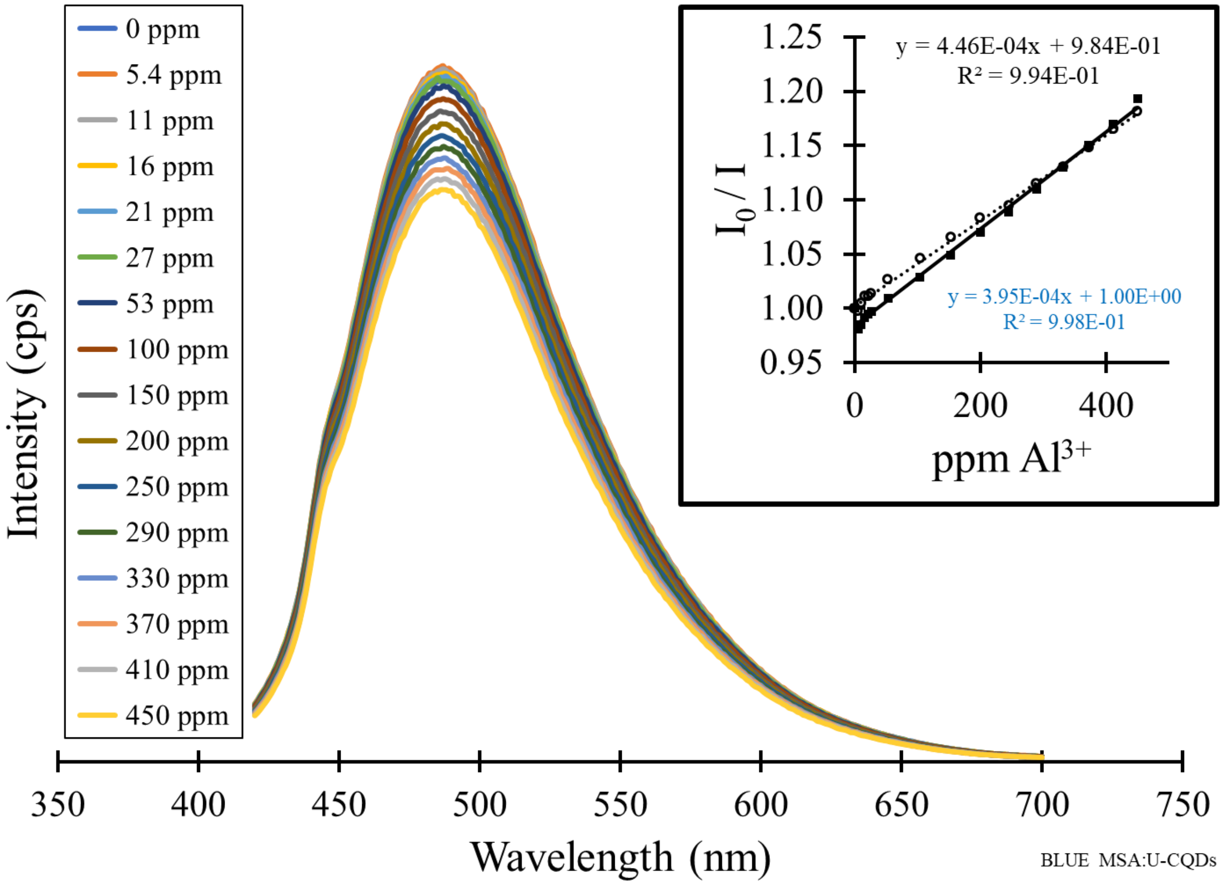

Figure S29. Steady-state fluorescence spectra (λ_{EXC} = 350 nm) of blue MSA-CQDs with added Al^{3+}. Inset is the corresponding Stern-Volmer plot (■ = CQD interaction with Al^{3+}, ○ = control experiment with the same volumes of water added as metal ion solution.

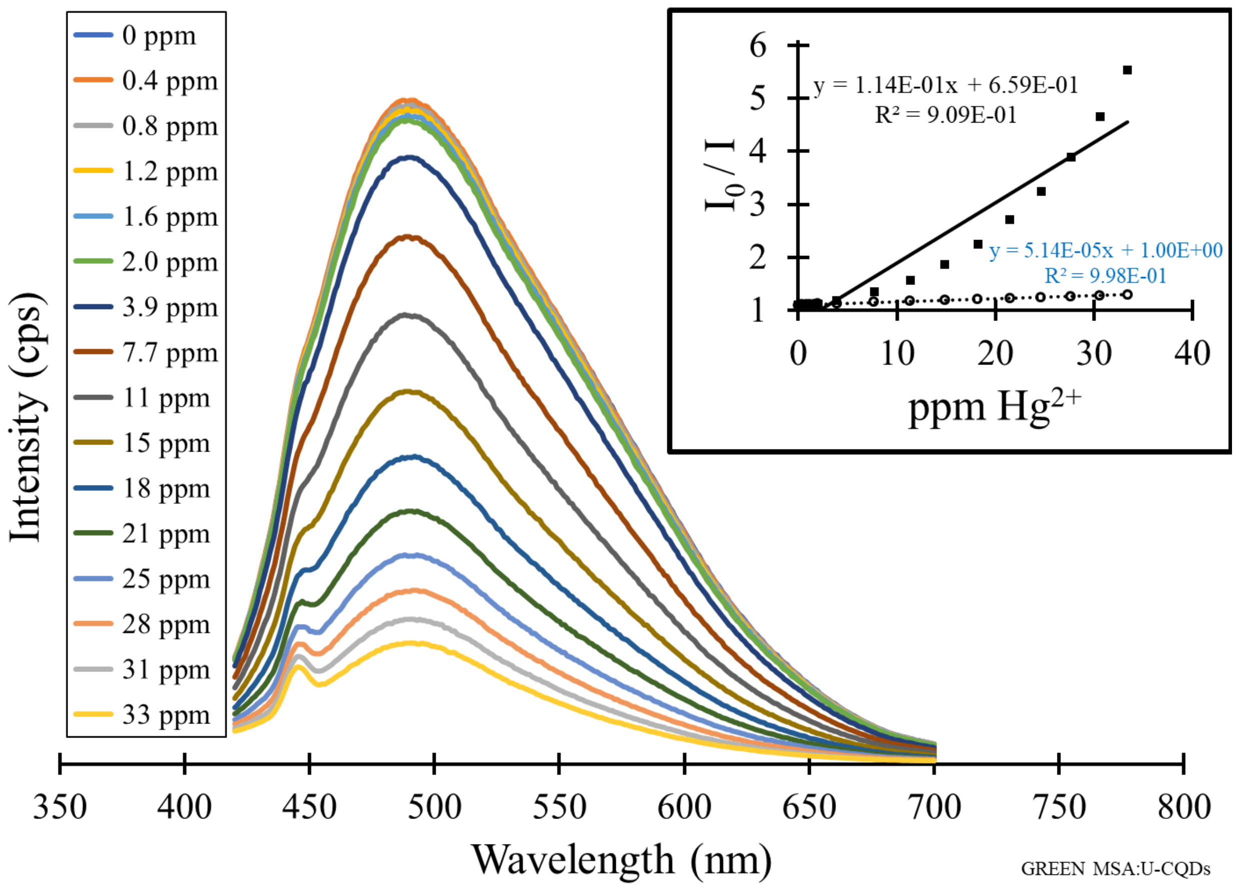

Figure S30. Steady-state fluorescence spectra (λ_{EXC} = 350 nm) of green MSA-CQDs with added Hg^{2+}. Inset is the corresponding Stern-Volmer plot (■ = CQD interaction with Hg^{2+}, ○ = control experiment with the same volumes of water added as metal ion solution.

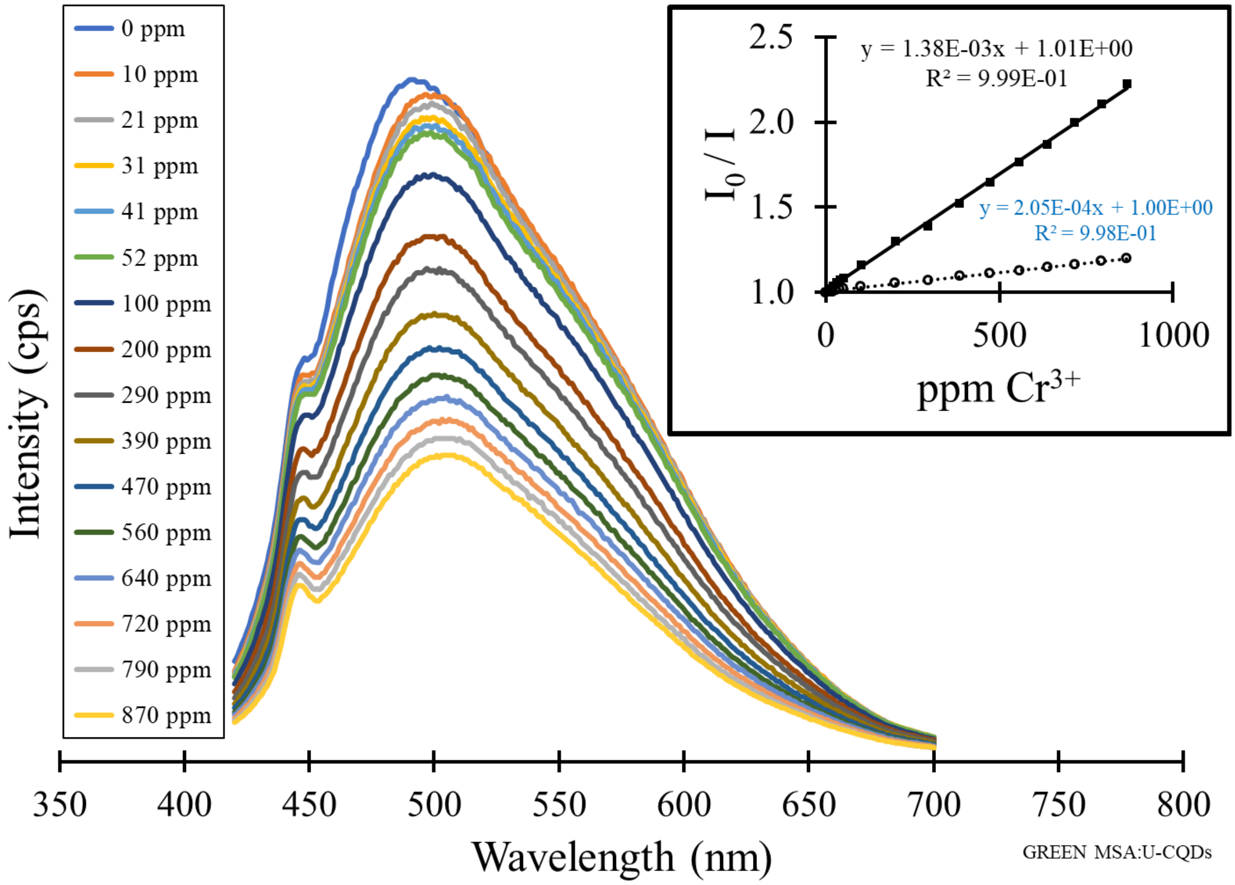

Figure S31. Steady-state fluorescence spectra (λ_{EXC} = 350 nm) of green MSA-CQDs with added Cr^{3+}. Inset is the corresponding Stern-Volmer plot (■ = CQD interaction with Cr^{3+}, ○ = control experiment with the same volumes of water added as metal ion solution.

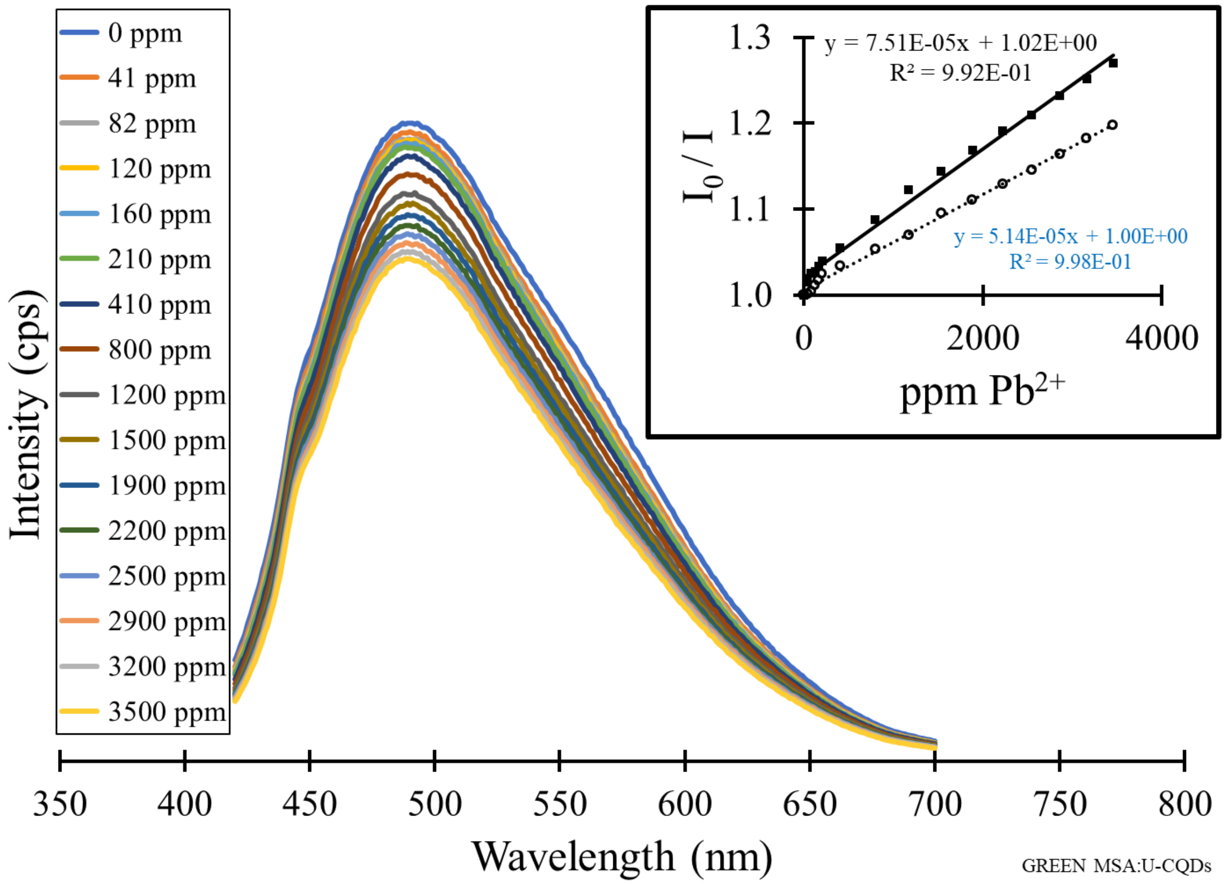

Figure S32. Steady-state fluorescence spectra (λ_{EXC} = 350 nm) of green MSA-CQDs with added Pb^{2+}. Inset is the corresponding Stern-Volmer plot (■ = CQD interaction with Pb^{2+}, ○ = control experiment with the same volumes of water added as metal ion solution.

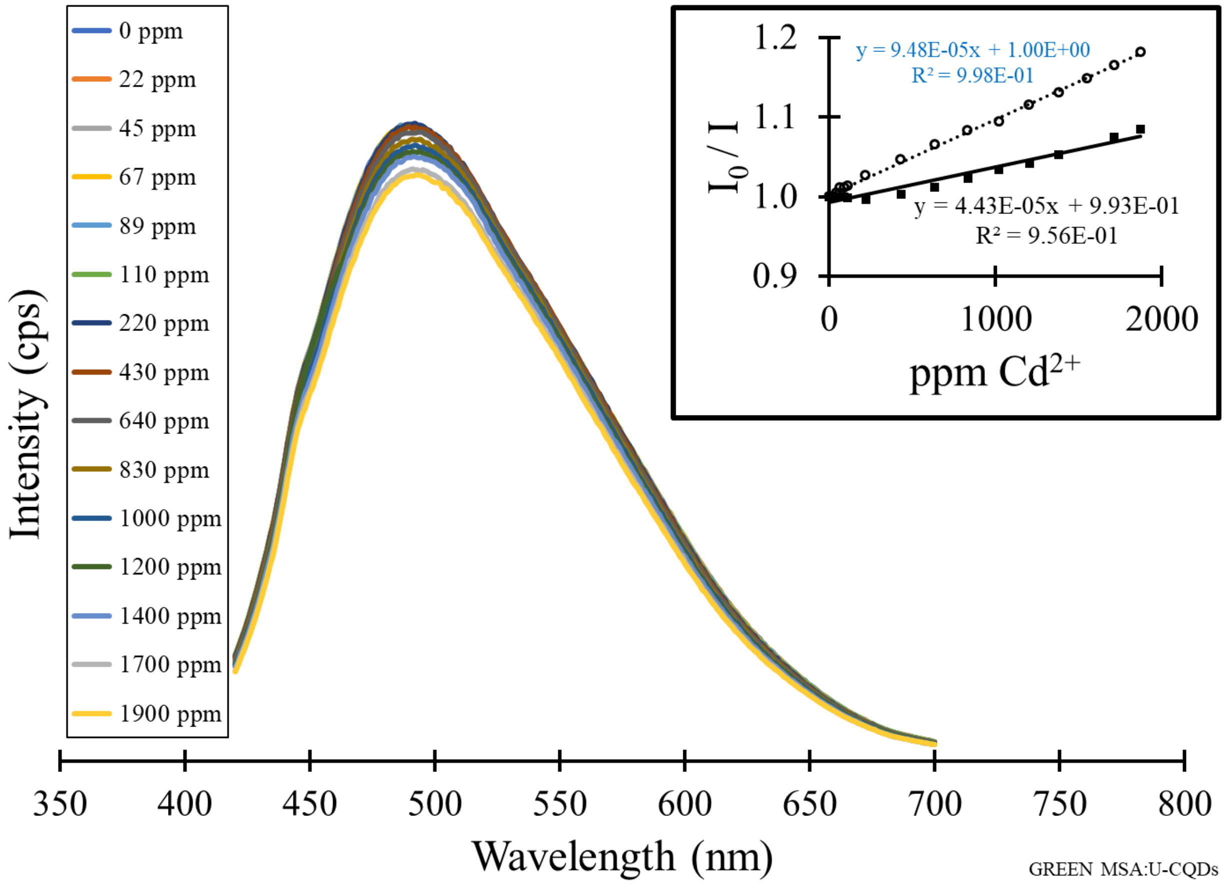

Figure S33. Steady-state fluorescence spectra (λ_{EXC} = 350 nm) of green MSA-CQDs with added Cd^{2+}. Inset is the corresponding Stern-Volmer plot (■ = CQD interaction with Cd^{2+}, ○ = control experiment with the same volumes of water added as metal ion solution.

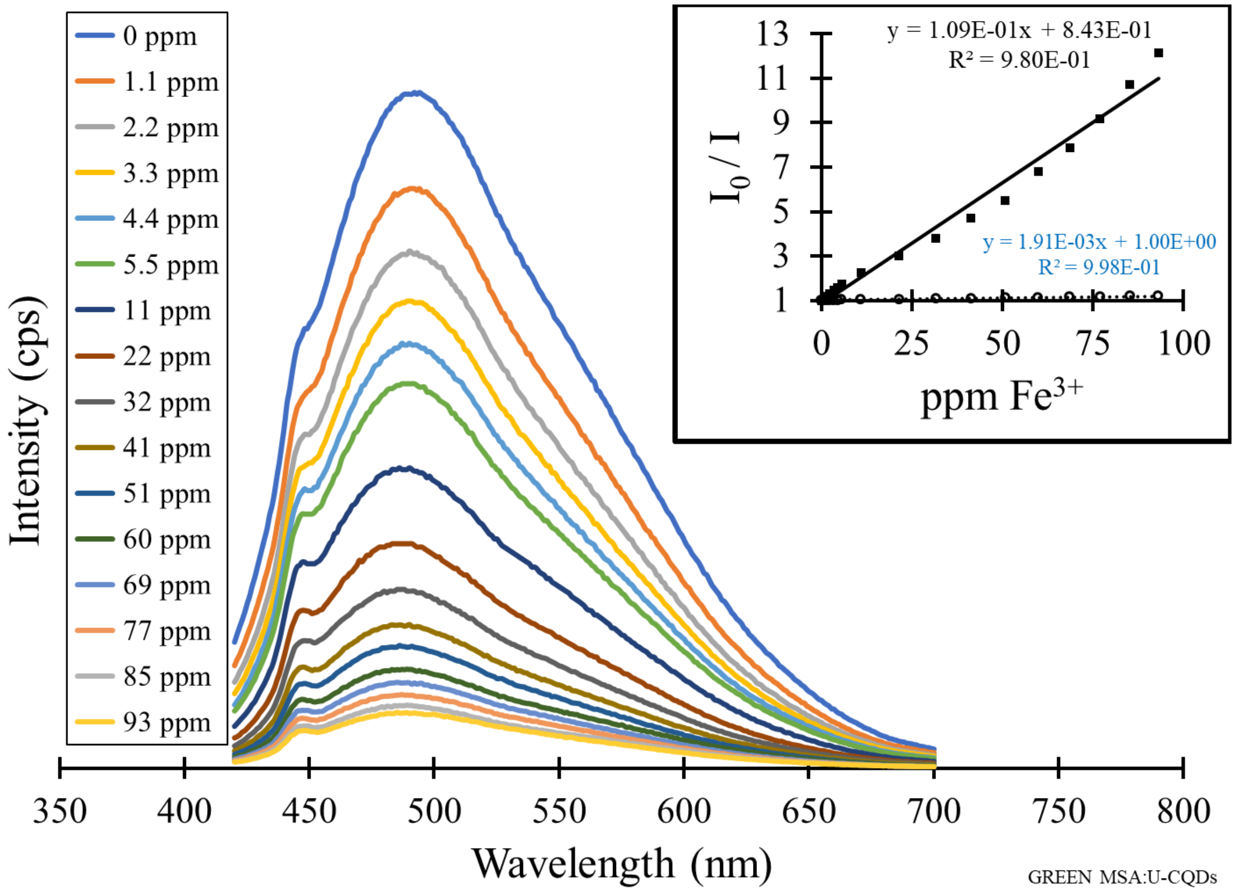

Figure S34. Steady-state fluorescence spectra (λ_{EXC} = 350 nm) of green MSA-CQDs with added Fe^{3+}. Inset is the corresponding Stern-Volmer plot (■ = CQD interaction with Fe^{3+}, ○ = control experiment with the same volumes of water added as metal ion solution.

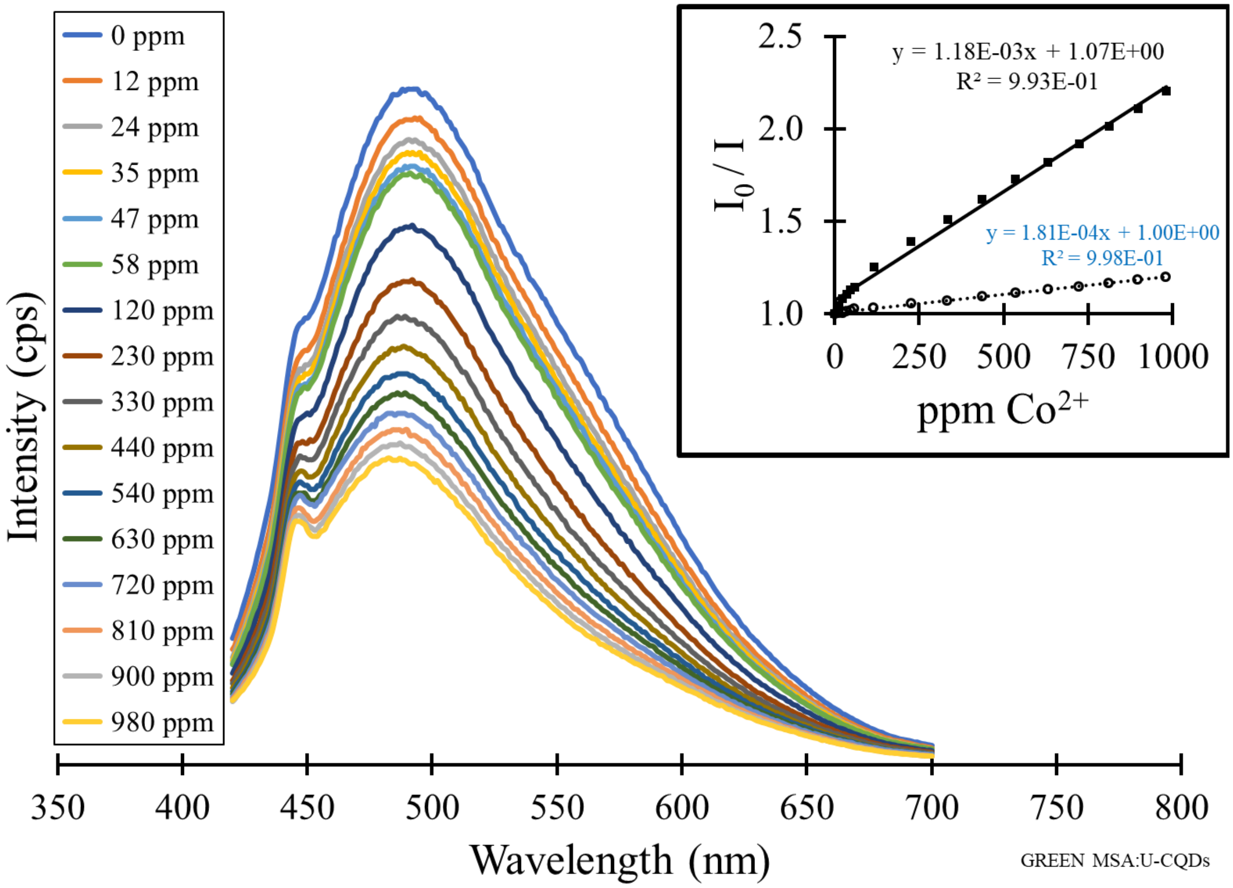

Figure S35. Steady-state fluorescence spectra (λ_{EXC} = 350 nm) of green MSA-CQDs with added Co^{2+}. Inset is the corresponding Stern-Volmer plot (■ = CQD interaction with Co^{2+}, ○ = control experiment with the same volumes of water added as metal ion solution.

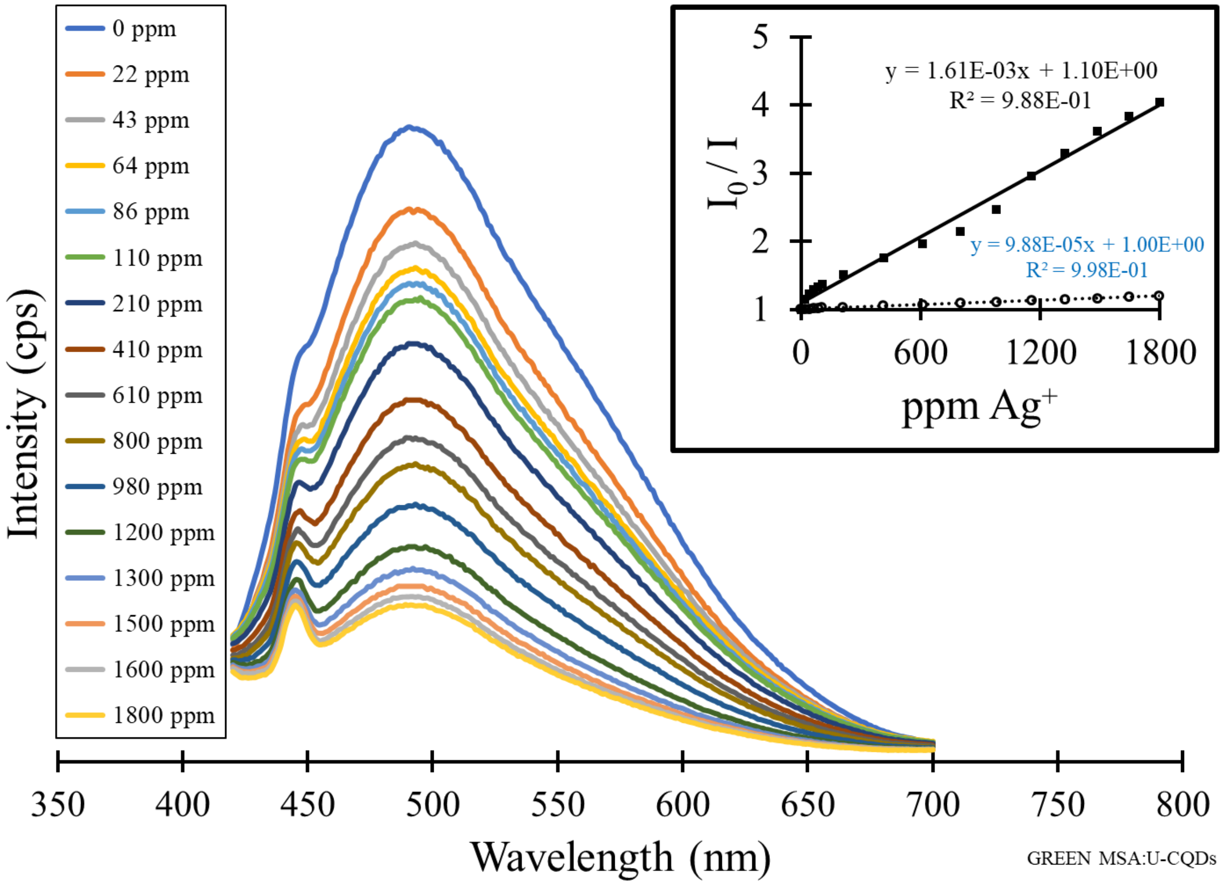

Figure S36. Steady-state fluorescence spectra (λ_{EXC} = 350 nm) of green MSA-CQDs with added Ag^+. Inset is the corresponding Stern-Volmer plot (■ = CQD interaction with Ag^+, ○ = control experiment with the same volumes of water added as metal ion solution.

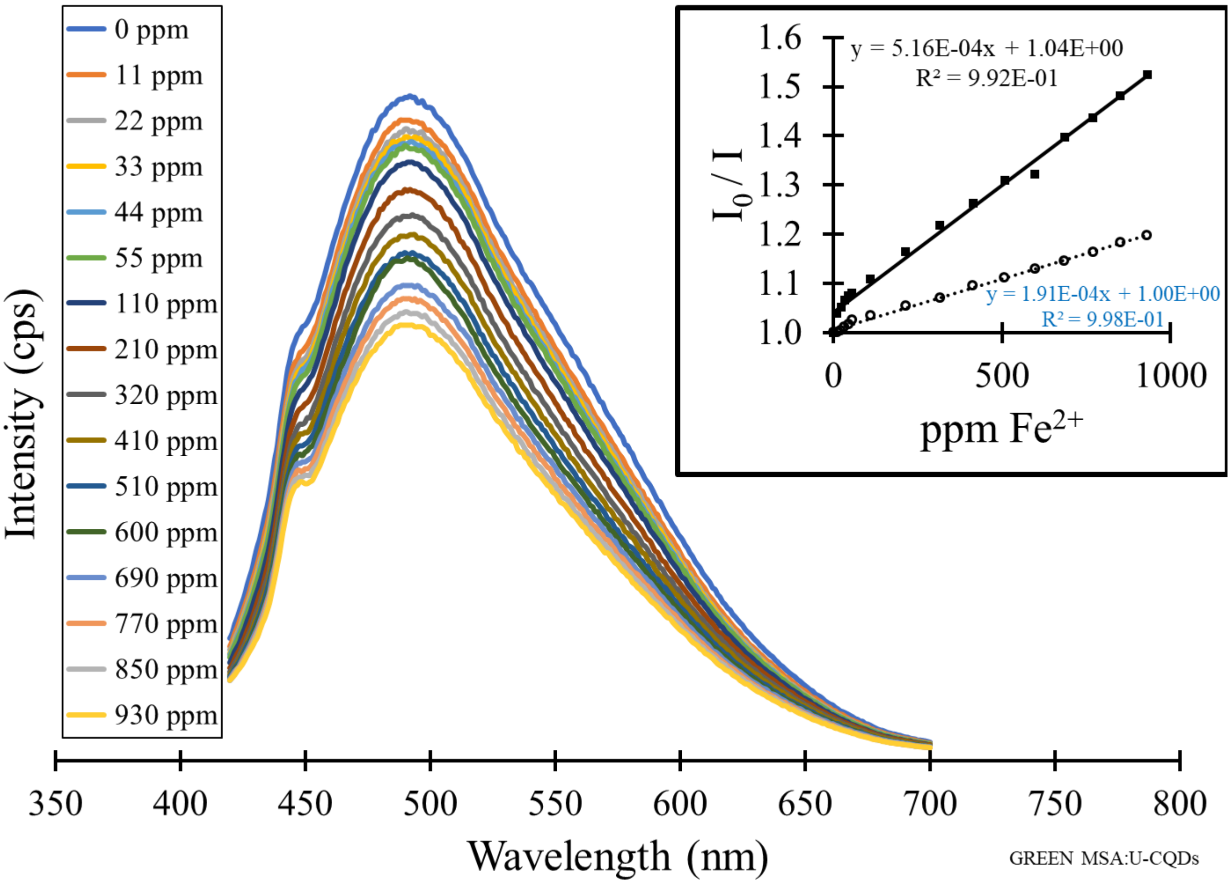

Figure S37. Steady-state fluorescence spectra (λ_{EXC} = 350 nm) of green MSA-CQDs with added Fe^{2+}. Inset is the corresponding Stern-Volmer plot (■ = CQD interaction with Fe^{2+}, ○ = control experiment with the same volumes of water added as metal ion solution.

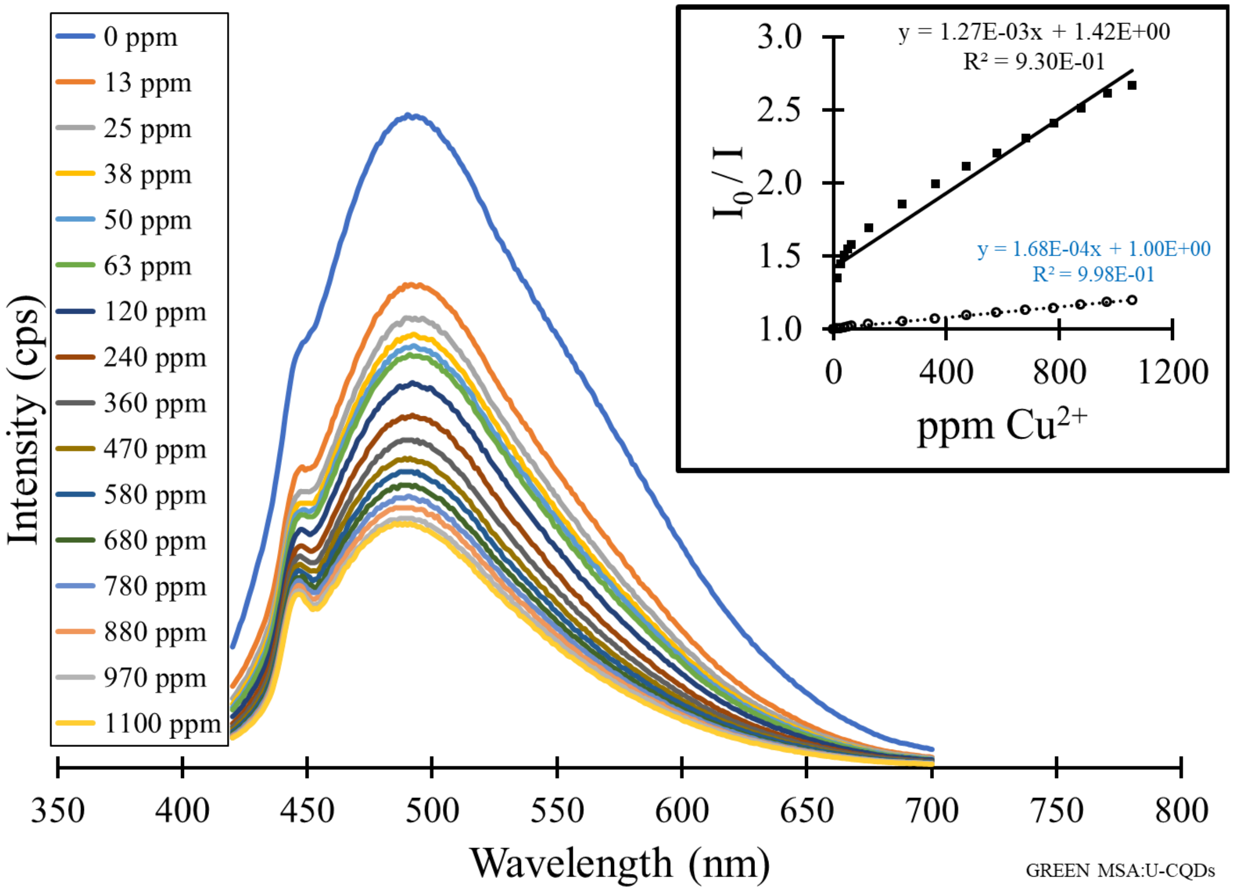

Figure S38. Steady-state fluorescence spectra (λ_{EXC} = 350 nm) of green MSA-CQDs with added Cu^{2+}. Inset is the corresponding Stern-Volmer plot (■ = CQD interaction with Cu^{2+}, ○ = control experiment with the same volumes of water added as metal ion solution.

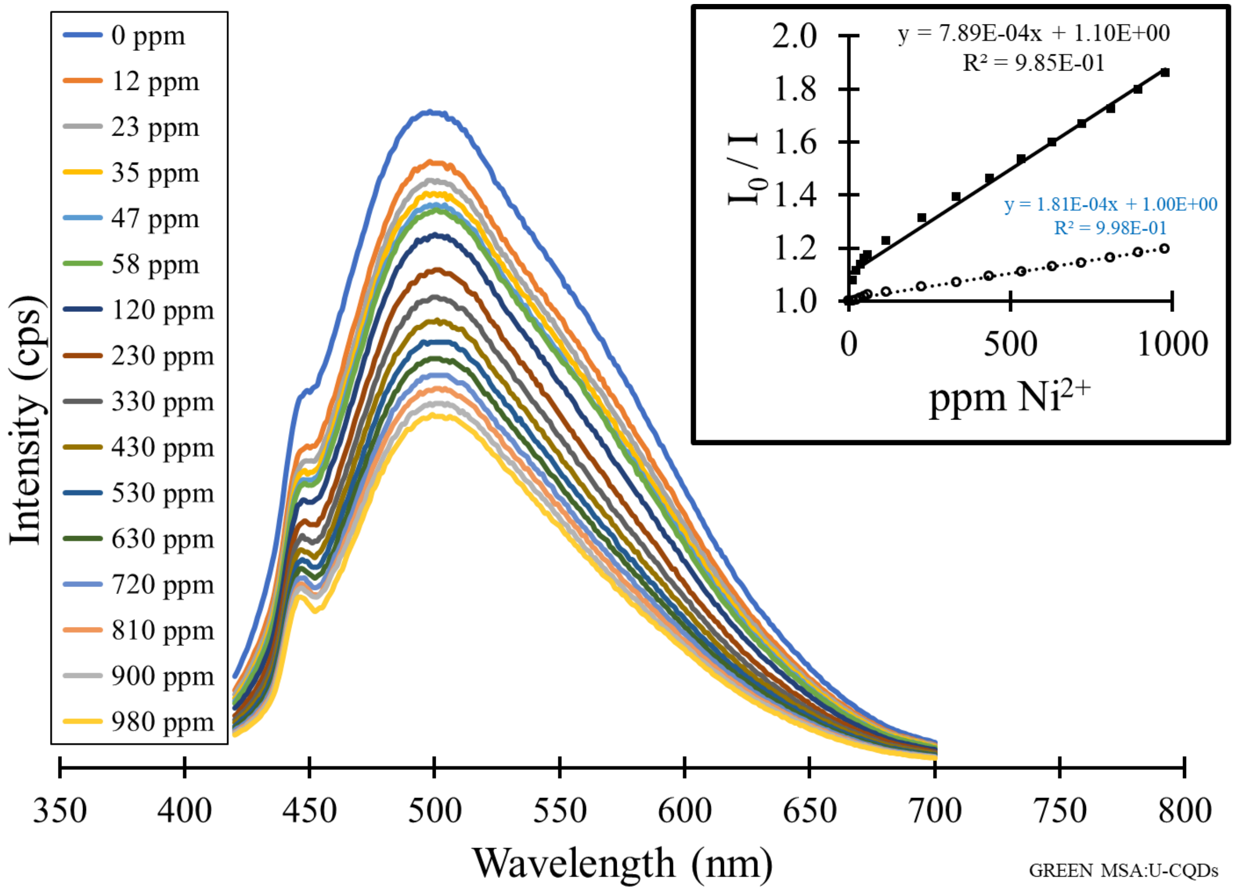

Figure S39. Steady-state fluorescence spectra (λ_{EXC} = 350 nm) of green MSA-CQDs with added Ni^{2+}. Inset is the corresponding Stern-Volmer plot (■ = CQD interaction with Ni^{2+}, ○ = control experiment with the same volumes of water added as metal ion solution.

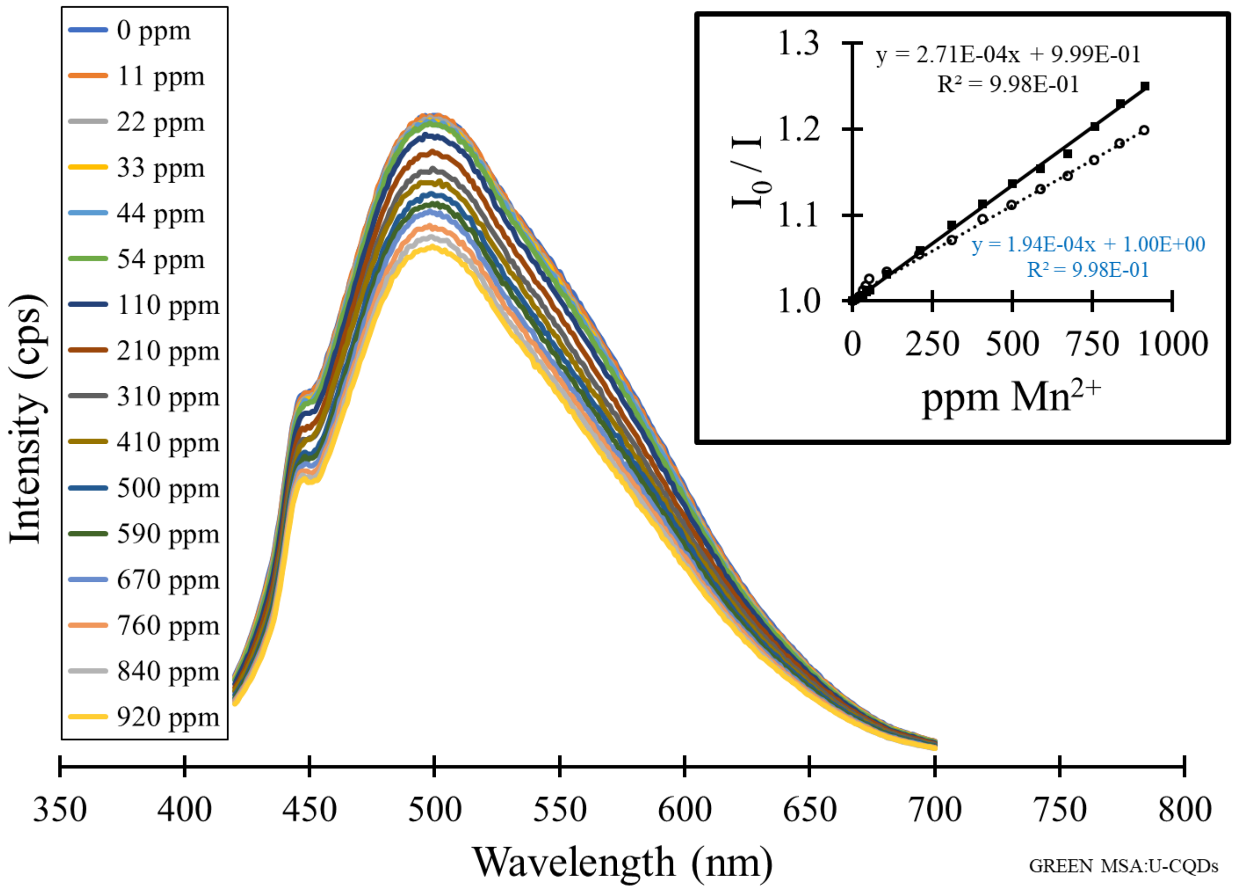

Figure S40. Steady-state fluorescence spectra (λ_{EXC} = 350 nm) of green MSA-CQDs with added Mn^{2+}. Inset is the corresponding Stern-Volmer plot (■ = CQD interaction with Mn^{2+}, ○ = control experiment with the same volumes of water added as metal ion solution.

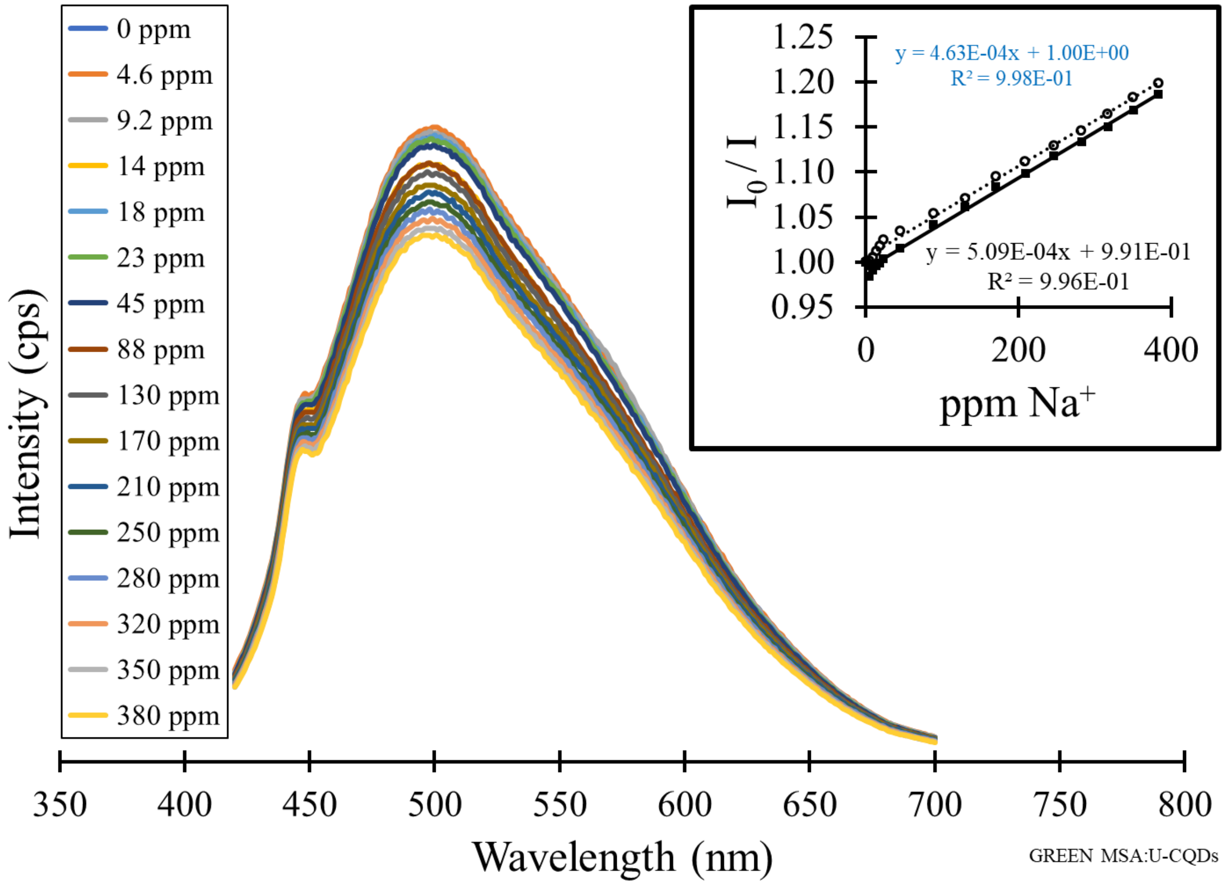

Figure S41. Steady-state fluorescence spectra (λ_{EXC} = 350 nm) of green MSA-CQDs with added Na^+. Inset is the corresponding Stern-Volmer plot (■ = CQD interaction with Na^+, ○ = control experiment with the same volumes of water added as metal ion solution.

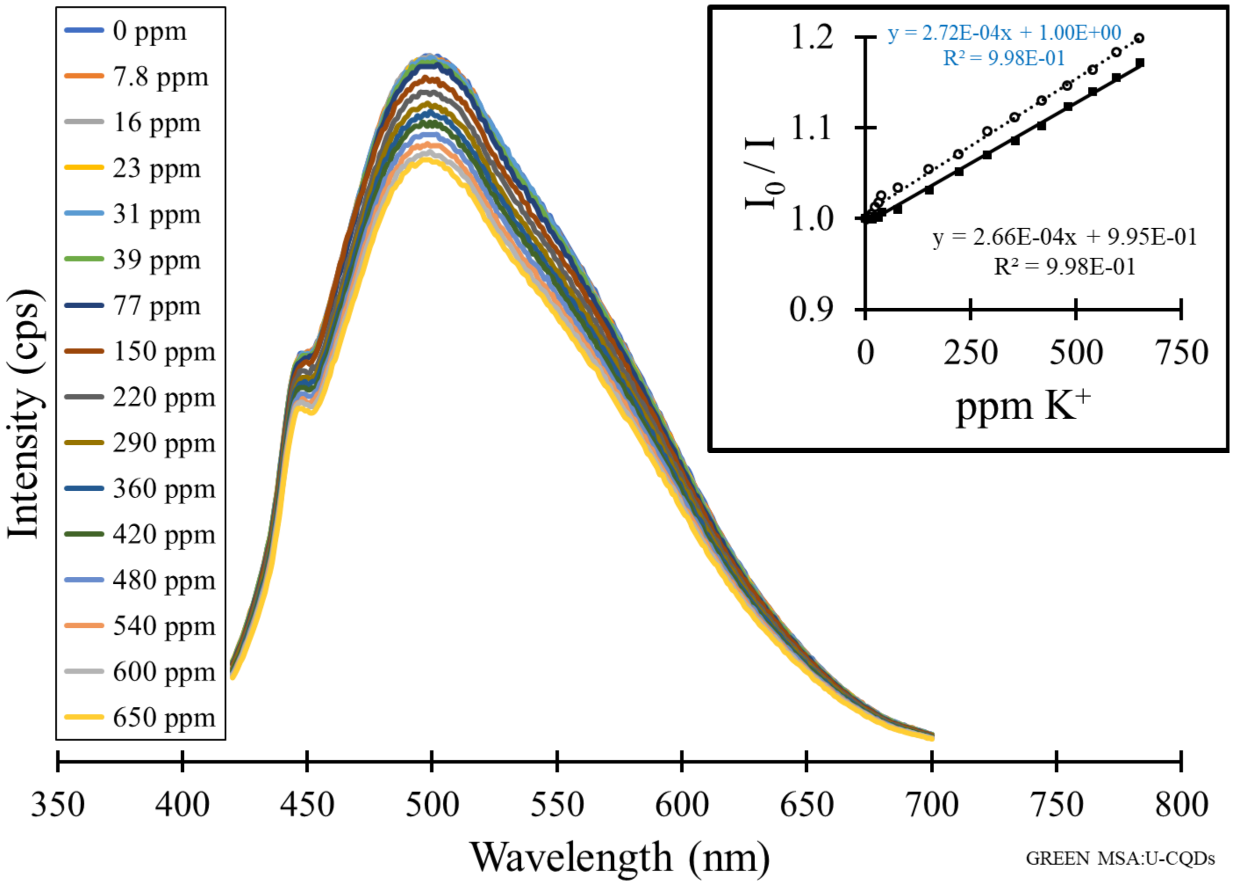

Figure S42. Steady-state fluorescence spectra (λ_{EXC} = 350 nm) of green MSA-CQDs with added K^+. Inset is the corresponding Stern-Volmer plot (■ = CQD interaction with K^+, ○ = control experiment with the same volumes of water added as metal ion solution.

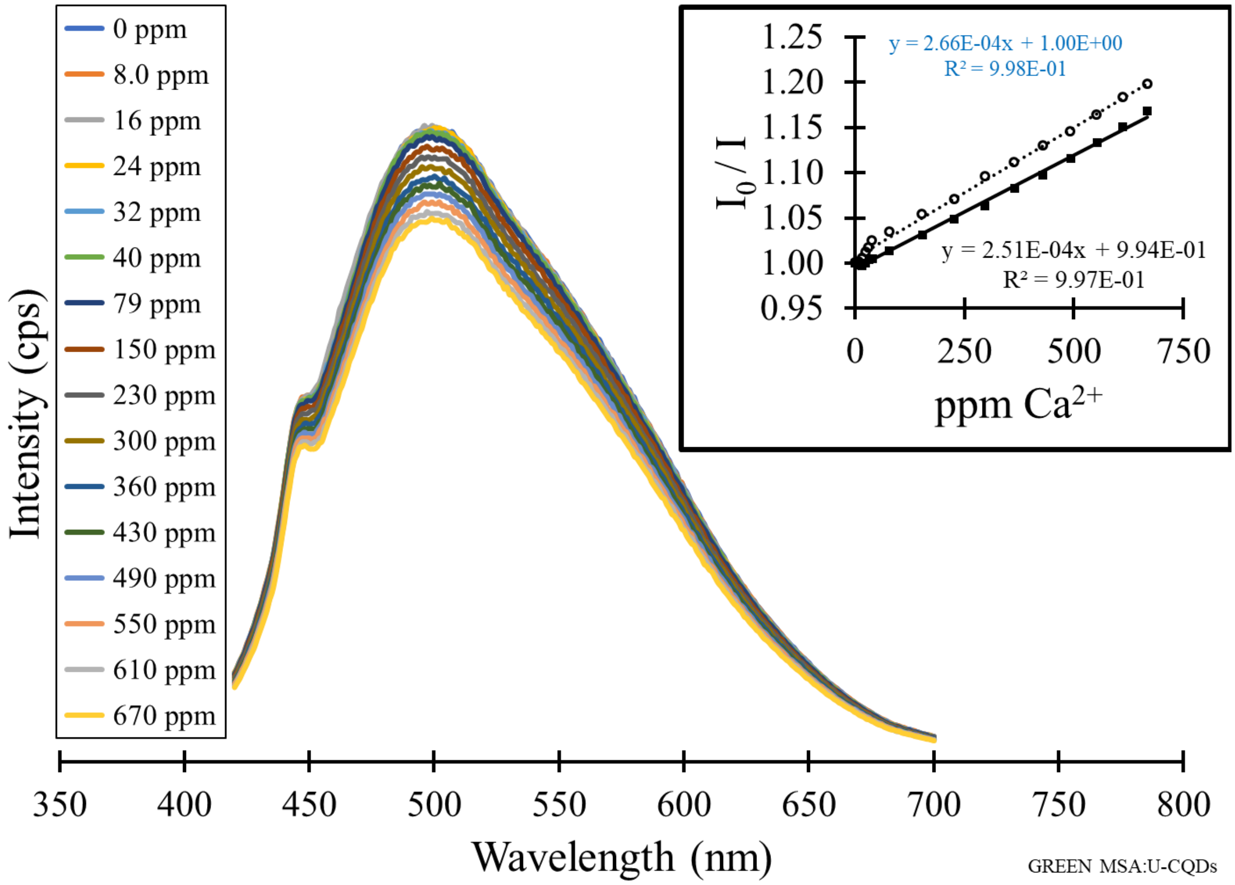

Figure S43. Steady-state fluorescence spectra (λ_{EXC} = 350 nm) of green MSA-CQDs with added Ca^{2+}. Inset is the corresponding Stern-Volmer plot (■ = CQD interaction with Ca^{2+}, ○ = control experiment with the same volumes of water added as metal ion solution.

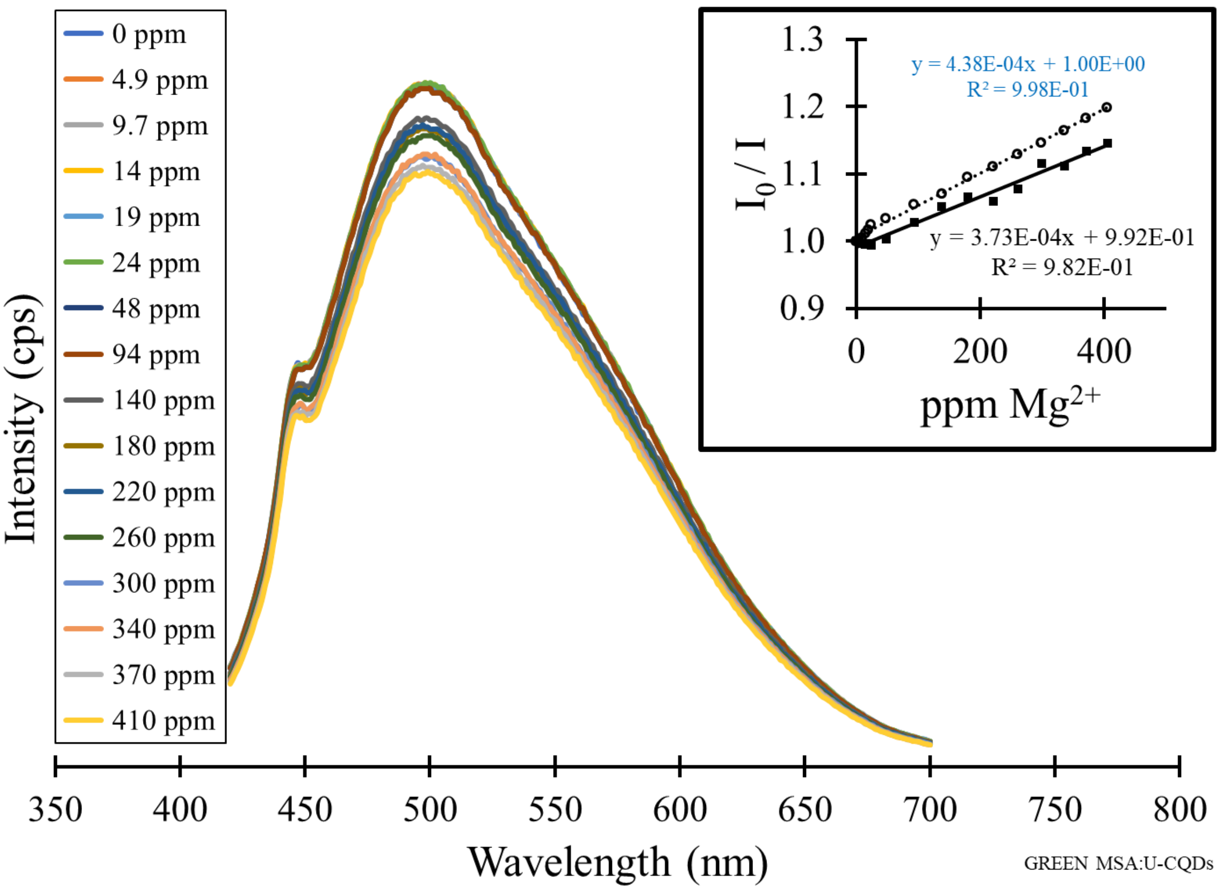

Figure S44. Steady-state fluorescence spectra (λ_{EXC} = 350 nm) of green MSA-CQDs with added Mg^{2+}. Inset is the corresponding Stern-Volmer plot (■ = CQD interaction with Mg^{2+}, ○ = control experiment with the same volumes of water added as metal ion solution.

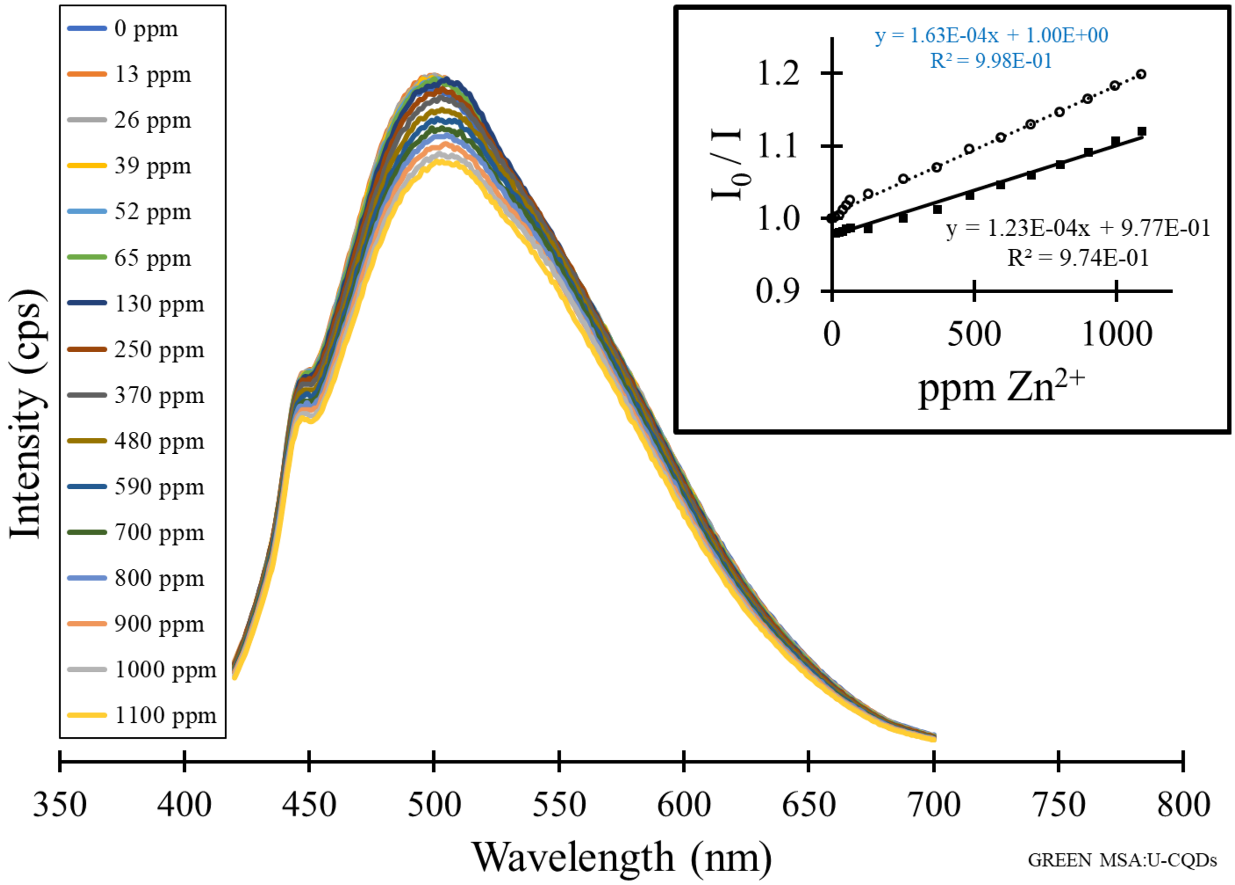

Figure S45. Steady-state fluorescence spectra (λ_{EXC} = 350 nm) of green MSA-CQDs with added Zn^{2+}. Inset is the corresponding Stern-Volmer plot (■ = CQD interaction with Zn^{2+}, ○ = control experiment with the same volumes of water added as metal ion solution.

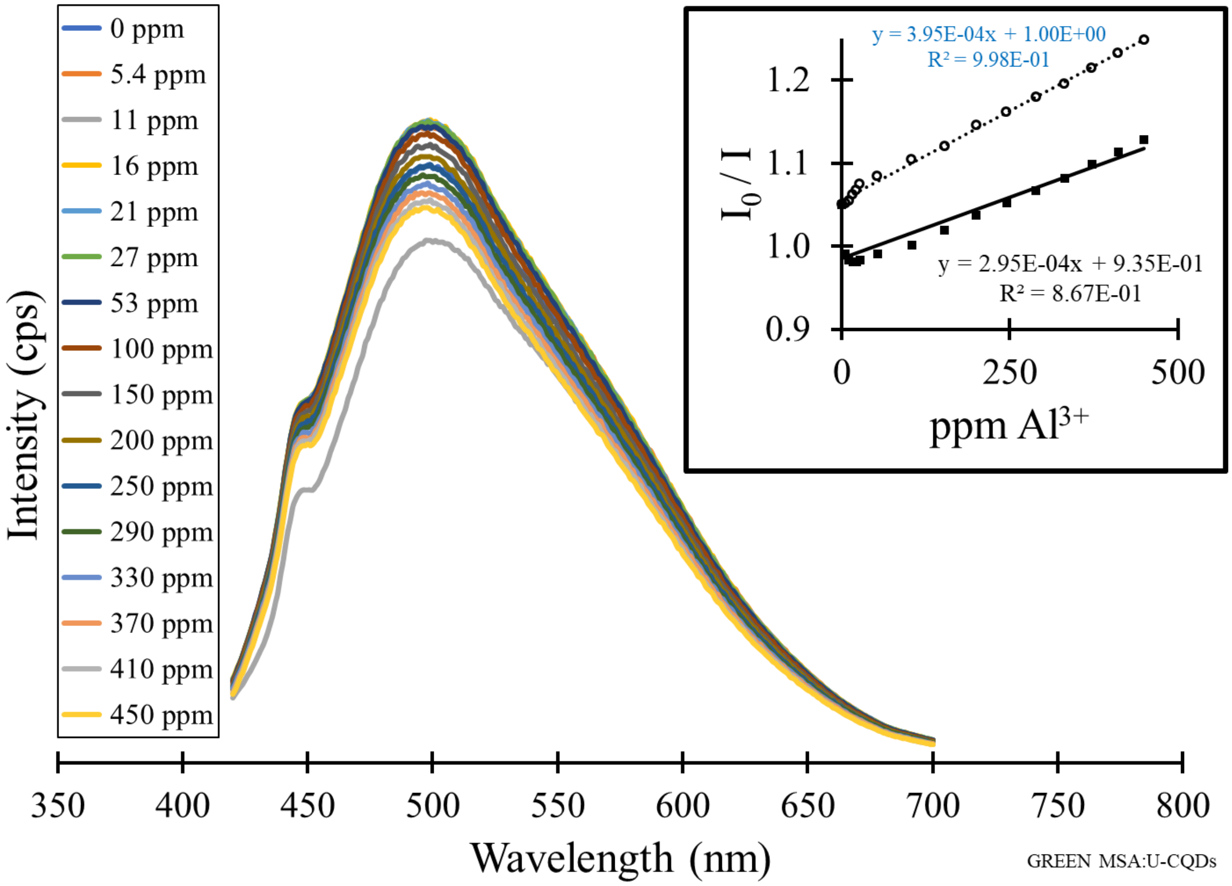

Figure S46. Steady-state fluorescence spectra (λ_{EXC} = 350 nm) of green MSA-CQDs with added Al^{3+}. Inset is the corresponding Stern-Volmer plot (■ = CQD interaction with Al^{3+}, ○ = control experiment with the same volumes of water added as metal ion solution.

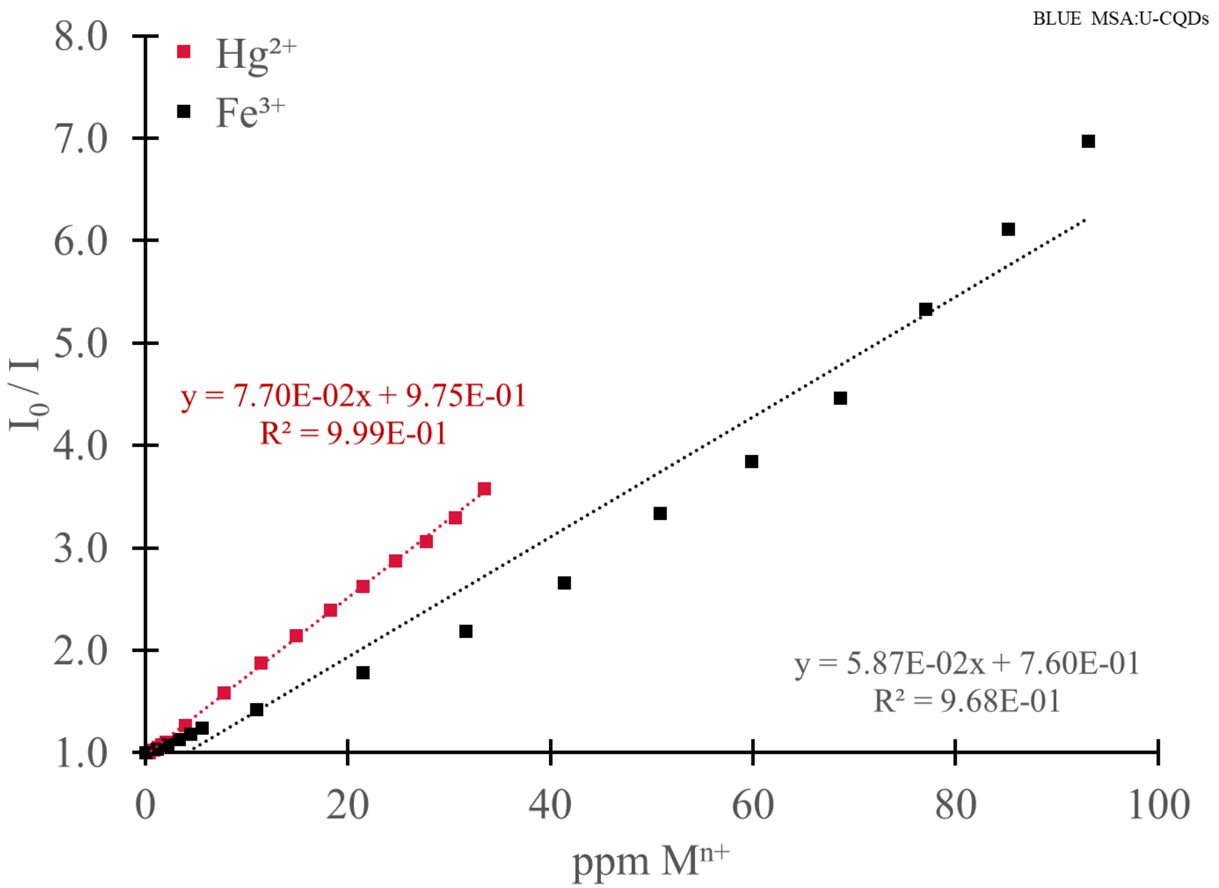

Figure S47. Stern-Volmer plots of blue MSA-CQDs with added Hg^{2+} and Fe^{3+}.

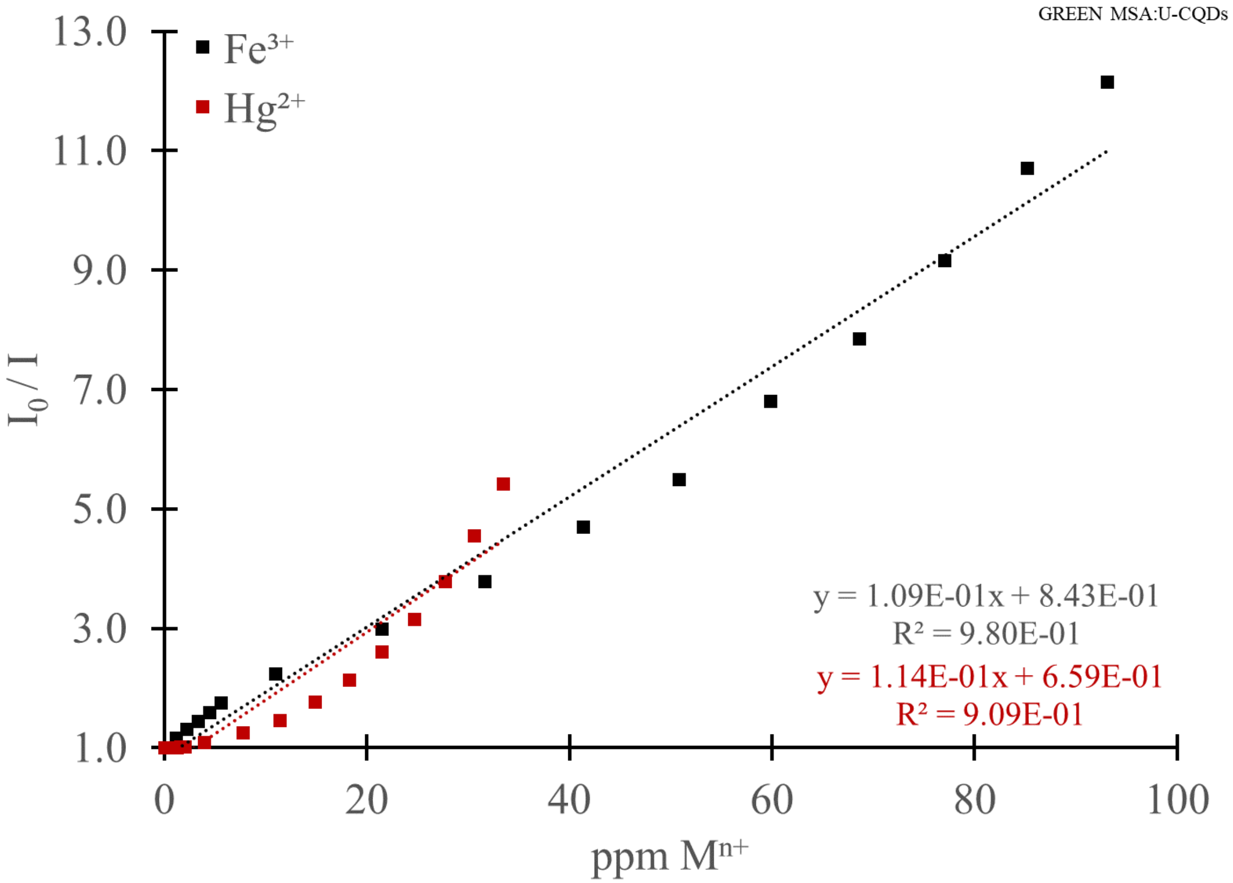

Figure S48. Stern-Volmer plots of green MSA-CQDs with added Fe^{3+} and Hg^{2+}.
